# International evaluation of the SEIZUre Risk in Encephalitis (SEIZURE) score for predicting acute seizure risk

**DOI:** 10.1136/bmjopen-2025-099451

**Published:** 2025-12-07

**Authors:** Thomas Hughes, Arun Venkatesan, Claire Hetherington, Franklyn Nkongho Egbe, M Netravathi, Kiran T Thakur, Betul Baykan, Tan Hui Jan, Susana Arias, Jesús García-de Soto, Jamil Kahwagi, Alberto Vogrig, Salvatore Versace, Ralph Habis, Swathi Sowmitran, Khalil S Husari, John Probasco, Rodrigo Hasbun, Paris Bean, Ashley Heck, Gökçen R GözübatıkÇelik, Dilek Ataklı, Fusun Mayda Domac, Vitor Ferreira, Sofia Calado, Thuppanattumadam Ananthasubramanian Sangeeth, Sylviane Defres, Marina Romozzi, Raffaele Iorio, Umberto Pensato, Maria Pleshkevich, Claude Steriade, Athena Sharifi-Razavi, Nasim Tabrizi, Jussi Sipila, Carla Y Kim, Alexandra Diaz-Ariza, Poorvikha Satish, Vinutha Gowda, Chandrakanta Gowda, Seong-il Oh, Luis del Carpio-Orantes, Mariasofia Cotelli, Luís Ferreira, Maria Kovalchuk, Anna Goncharova, Tom Solomon, Andrea Winkler, Alla Guekht, Greta K Wood, On Behalf of The Global NeuroResearch Coalition, Benedict D Michael

**Affiliations:** 1 Clinical Infection, Microbiology, and Immunology, Institute of Infection, Veterinary and Ecological Sciences, University of Liverpool, Liverpool, UK; 2 Department of Neurology, John Hopkins University School of Medicine, Baltimore, Maryland, USA; 3 Department of Neurology, NIMHANS, Bangalore, Karnataka, India; 4 Neurology, Columbia University Medical Center, New York, New York, USA; 5 Department of Neurology, EMAR Medical Center, Istanbul, Turkey; 6 Department of Medicine, National University of Malaysia, Bangi, Selangor, Malaysia; 7 Neurology, Hospital Clínico Universitario, Santiago de Compostela, A Coruña, Spain; 8 Neurology, Hospital Universitario Lucus Augusti, Lugo, Spain; 9 University Hospital Fann, Dakar, Senegal; 10 Department of Medicine, University of Udine Azienda Sanitaria Universitaria Friuli Centrale, Udine, Italy; 11 Neurology, Johns Hopkins University, Baltimore, Maryland, USA; 12 Department of Neurology, The University of Texas at Austin, Austin, Texas, USA; 13 University of Health Sciences, Bakirkoy Mazhar Osman Mental Health and Neurological Diseases Training and Research Hospital, Istanbul, Turkey; 14 University of health sciences, Erenkoy Mental Health and Neurological Diseases Training and Research Hospital, Istanbul, Turkey; 15 Neurology Department, Hospital de Egas Moniz Serviço de Neurologia, Lisboa, Portugal; 16 Clinical Infection Microbiology and Immunology, University of Liverpool Institute of Infection and Global Health, Liverpool, UK; 17 Tropical and Infectious Diseases Unit, Royal Liverpool and Broadgreen University Hospitals NHS Trust, Liverpool, UK; 18 Dipartimento di Neuroscienze, Fondazione Policlinico Universitario Agostino Gemelli IRCCS, Roma, Lazio, Italy; 19 Dipartimento di Neuroscienze, Università Cattolica del Sacro Cuore, Milano, Lombardia, Italy; 20 IRCCS Humanitas Research Hospital, Rozzano, Italy; 21 Department of Biomedical Sciences, Humanitas University, Milan, Italy; 22 Department of Neurology, NYU Langone Medical Center, New York, New York, USA; 23 Department of Psychology, Suffolk University, Boston, Massachusetts, USA; 24 NYU Langone Medical Center, New York, New York, USA; 25 Department of Neurology, Mazandaran University of Medical Sciences, Sari, Iran, Islamic Republic of; 26 Cclinical Neurosciences, University of Turku, Turku, Varsinais-Suomi, Finland; 27 Department of Neurology, North Karelia Central Hospital, Joensuu, Finland; 28 Department of Neurology, St John’s Medical College Hospital, Bangalore, Karnataka, India; 29 Department of Neurology, Busan Paik Hospital, Inje University College of Medicine, Busan, Korea (the Republic of); 30 Department of Neurology, Kyung Hee University Hospital, Dongdaemun-gu, Seoul, Korea (the Republic of); 31 Department of Internal Medicine, Instituto Mexicano del Seguro Social, Ciudad de Mexico, Mexico; 32 Neurology Unit, ASST della Valcamonica, Breno, Lombardia, Italy; 33 Centro Hospitalar Universitário de Santo António, Porto, Portugal; 34 Buyanov City Hospital, Moscow, Russian Federation; 35 Pirogov Russian National Research Medical University, Moskva, Russian Federation; 36 Institute of Infection and Global Health, University of Liverpool, Liverpool, UK; 37 Department of Neurology, Technical University of Munich Hospital Rechts der Isar Neuro-Head Center, Munchen, Bayern, Germany; 38 Department of Community Medicine and Global Health, University of Oslo Department of Health and Society, Oslo, Norway; 39 Department of Global Health and Social Medicine, Harvard Medical School, Boston, Massachusetts, USA; 40 University of Liverpool Department of Clinical Infection Microbiology and Immunology, Liverpool, UK

**Keywords:** Epilepsy, Neurology, Neuropathology

## Abstract

**Abstract:**

**Objective:**

Encephalitis is brain parenchyma inflammation, frequently resulting in seizures which worsens outcomes. Early anti-seizure medication could improve outcomes but requires identifying patients at greatest risk of acute seizures. The SEIZURE (SEIZUre Risk in Encephalitis) score was developed in UK cohorts to stratify patients by acute seizure risk. A ‘basic score’ used Glasgow Coma Scale (GCS), fever and age; the ‘advanced score’ added aetiology. This study aimed to evaluate the score internationally to determine its global applicability.

**Design:**

Patients were retrospectively analysed regionally, and by country, in this international evaluation study. Univariate analysis was conducted between patients who did and did not have inpatient seizures, followed by multivariable logistic regression, hierarchical clustering and analysis of the area under the receiver operating curves (AUROC) with 95% CIs.

**Participants and setting:**

2032 patients across 13 countries were identified, among whom 1324 were included in SEIZURE score calculations and 970 were included in regression modelling. The involved countries comprised 19 organisations spanning all WHO regions.

**Outcome measures:**

The primary outcome was measuring inpatient seizure rates.

**Results:**

Autoantibody-associated encephalitis, low GCS and presenting with a seizure were frequently associated with inpatient seizures; fever showed no association. Globally, the score had limited discriminatory ability (basic AUROC 0.58 (95% CI 0.55 to 0.62), advanced AUROC 0.63 (95% CI 0.60 to 0.66)). The scoring system performed acceptably in western Europe, excluding Spain, with the best performance in Portugal (basic AUROC 0.82 (95% CI 0.69 to 0.94), advanced AUROC 0.83 (95% CI 0.72 to 0.95)).

**Conclusions:**

The SEIZURE score performed best in several countries in Western Europe but performed poorly elsewhere, partly due to differing and unknown aetiologies. In most regions, the score did not reach a threshold to be clinically useful. The Western European results could aid in designing clinical trials assessing primary anti-seizure prophylaxis in encephalitis following further prospective trials. Beyond Western Europe, there is a need for tailored, localised scoring systems and future large-scale prospective studies with optimised aetiological testing to accurately identify high-risk patients.

**STRENGTHS AND LIMITATIONS OF THIS STUDY**

- The study consisted of one of the largest sample sizes of any encephalitis study with patient cohorts from every WHO region.
- Data collection was standardised to facilitate accurate comparisons and was centrally screened for accuracy prior to being included in analysis.
- Varying facilities between countries prevented inter-regional analysis of certain variables, while limited sample sizes in certain regions and the small number of paediatric cases reduced how representative the data is for certain populations.

## Introduction

 Encephalitis is inflammation of the brain parenchyma, typically due to infection or autoantibody-driven processes. Patients with encephalitis often develop acute seizures which are associated with increased morbidity and worse long-term outcomes, including epilepsy.^1 2^ Prophylactic anti-seizure medicines (ASMs) are not routinely used due to insufficient safety and efficacy evidence.^3^ It remains unclear whether the poorer outcomes are due to seizures acting as a proxy marker of disease severity or secondary damaging effects including cerebral hypoxia, hypoglycaemia or excitotoxic injury.^3^ However, identifying patients with encephalitis at greatest risk of acute seizures would facilitate risk stratification, to identify patients requiring high-level care including electroencephalogram (EEG) monitoring to identify subtle seizures, and clinical trials to determine whether primary ASM prophylaxis in high-risk patients will improve long-term outcomes, and consequently whether seizures independently worsen outcomes or simply indicate disease severity. The SEIZURE (SEIZUre Risk in Encephalitis) score was developed in two UK cohorts to stratify patients with encephalitis by seizure risk.^4^ Two scores were developed: a ‘basic score’ using the Glasgow Coma Scale (GCS), fever and age, and an ‘advanced score’ including aetiology. Both scores showed good discrimination to determine acute seizure risk in patients with encephalitis.^4^ Prior research has established age and GCS as risk factors for seizures of any cause and also specifically in patients with encephalitis.^25^,^7^ The SEIZURE score was the outcome of the first study to combine this prior knowledge into an applicable, validated, risk stratifying tool for UK patients with encephalitis. The utility of this score for patients with encephalitis globally is unknown. The primary aim of this study was to assess this scoring system globally and identify regions in which it can be used to identify high-risk patients, and areas that require tailored scoring systems. We hypothesise that the discriminatory ability of the scoring systems will vary, with poorer performances in countries with variations in aetiology and healthcare systems that are distinctly different from the initial UK cohorts.^38^,^10^ Secondary aims included identifying features associated with increased seizure risk in patients with encephalitis, comparing aetiologies of encephalitis between regions and analysis of the outcomes between different regions.

## Methods

### Data collection

Data collection was standardised using a case report form. Prespecified options in the case record form were used to maximise the integrity and consistency of data collection, based on the original SEIZURE score publication, with a free-text option for additional detail. Without access to the raw patient notes, it was not possible to audit the quality of clinical care. Responses were centrally screened to verify data collection accuracy and any omissions, with clear errors or inconsistencies highlighted to the study centre to recheck their data. Study sites were large tertiary centres and identified via established networks, including The Global Neuro Research Coalition, the World Federation of Neurology and Encephalitis International.^11^,^13^ Anonymised raw data were collected retrospectively by local neurologists from pre-existing datasets or via medical records using discharge coding and case-by-case review based on the WHO Acute Encephalitis Syndrome definition.^14^ Case ascertainment methods were not standardised, study centres were encouraged to provide their most recent 50 cases for consistency, and to capture current practice in diagnostics for autoimmune encephalitis. Pre-collected cases from prior research were also included to maximise the sample size. The data were centrally collated into a global group, following local ethical approval and data-sharing agreements where applicable, or centres could send deidentified pre-analysed data by established a priori methodology.^4^

**Figure 1:**
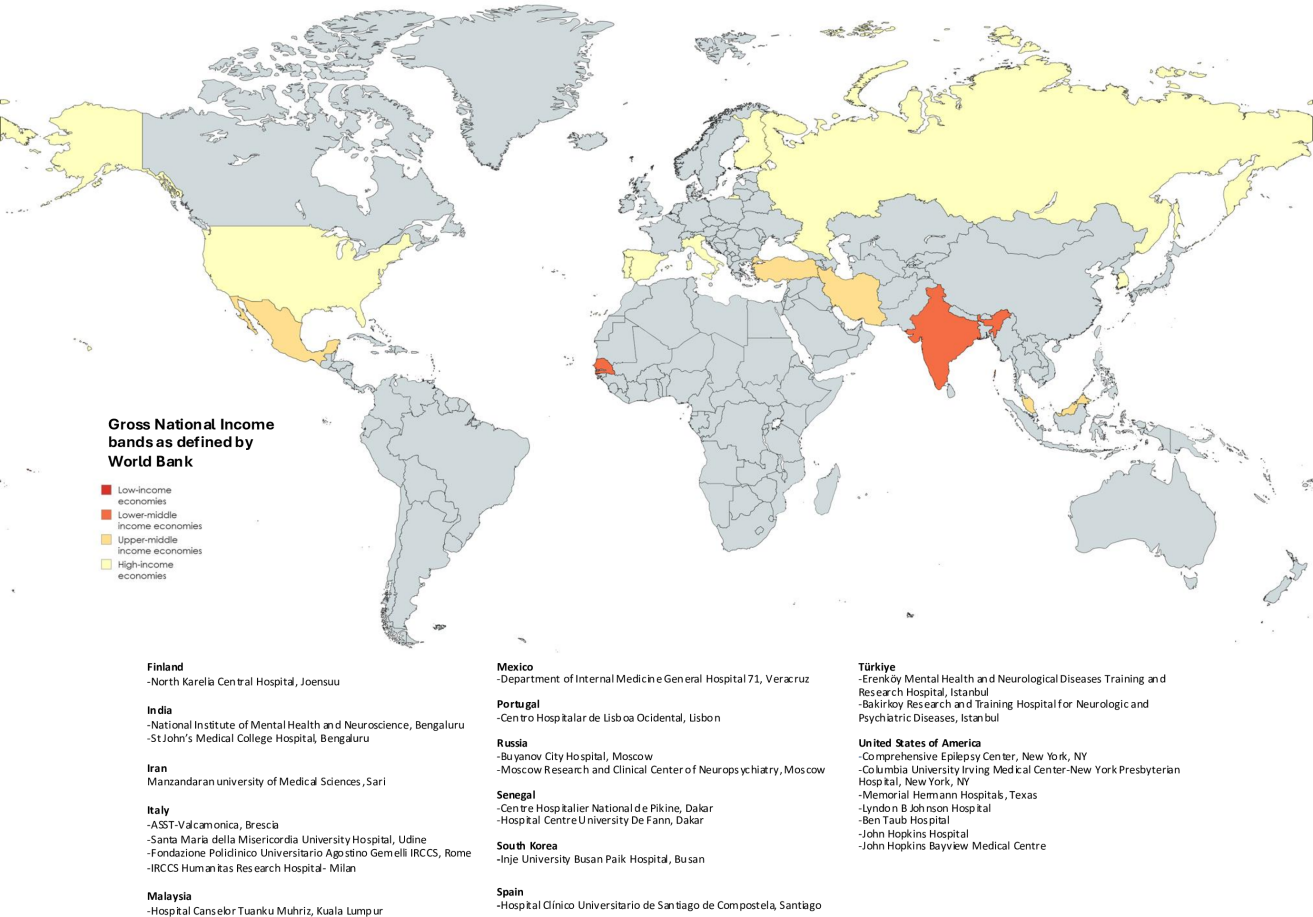
A world map detailing the countries involved in the study, separated into Gross National Income bands as per The World Bank. Specific hospitals and centres are noted below the map, divided by country.

Mandatory data included age, GCS, temperature on admission, presenting complaint of fever, aetiology and inpatient seizure status. Data collection additionally included demographics, symptomology and duration prior to admission, investigations and Glasgow Outcome Scale (GOS) score. Seizures were categorised as a ‘presenting symptom’, defined as seizures occurring in the community, or ‘inpatient seizures’, defined as witnessed seizures occurring following admission and categorised in accordance with the International League Against Epilepsy (ILAE) terminology.^15^ Electrographic seizures were included under ‘inpatient seizures’ in centres with continuous EEG monitoring. Status epilepticus (SE), as defined by the ILAE, was included within ‘inpatient seizures’ and also analysed separately. SE was only recorded for inpatient seizures due to potential inaccuracies with community diagnoses and in line with the primary aim of the study, which was to evaluate the utility of the score in predicting those at risk of seizures during admission, and therefore potentially amenable to prophylactic therapy. SE subtypes were included, but the sample size was inadequate for subgroup analysis. Admission dates ranged from 2010 to 2023 and data collection took place retrospectively over a 14-month period (February 2023 to April 2024), including 19 organisations from 13 countries across all WHO regions (figure 1).

The primary outcome was inpatient seizure. Age was categorised as required for the SEIZURE score.^4^ GCS was inputted as an ordinal variable for scoring and categorical variable (normal, 15; mild impairment, 13–14; moderate impairment, 9–12; severe impairment, 3–8) for univariate analysis. For multivariable analysis, it was treated as a continuous variable.

Aetiology was categorised identically to Wood *et al* using the same diagnostic criteria.^4^ Fever was defined as ≥38°C or a history of fever. Immunosuppressed categories included metabolic, drug-induced, congenital, pregnancy, HIV and malignancy. EEG analysis categories were simplified as per Wood *et al* to normal, focal abnormalities or consistent with encephalitis; MRI was either normal or abnormal.^4^ A high lactate was defined as ≥4 mmol/L, low sodium as ≤120 mmol/L, and a low cerebrospinal fluid (CSF):serum glucose ratio was defined as <0.5. A poor outcome was defined as a GOS score of <5 and a good recovery as GOS=5 at 6 months post-discharge.

### Statistical analysis

All data were collated into a global population for SEIZURE score calculations, including groups that provided pre-calculated SEIZURE scores. Where raw data were available, a global dataset was created for further analysis. Data were also separated into WHO regions and individual countries where possible. Univariate analysis was conducted using Prism-10 (V.10.2.2). Categorical variables were analysed using χ^2^ or Fisher’s exact test, parametric continuous variables were analysed using Student’s t-test, and non-parametric data using Mann-Whitney U test. ORs were reported with 95% CIs, and standard two-sided p<0.05 criteria was used for determining statistical significance. For univariate analysis, when calculating OR for variables with multiple discrete categories, including age and aetiology, the reference value was defined as the combined sum of the remaining categories. The exceptions for this were GCS, which consistently used 15 as a reference score, and the SEIZURE score categories. The SEIZURE score categories were defined using the sum of the two lowest-scoring categories as a reference range, consistent with the original Wood *et al* study. The categories were kept separate to allow for visualisation of the distribution of scores among the groups. Sensitivities, specificities and receiver operating curves (ROC) were calculated, reported as area under the receiver operating curve (AUROC) with 95% CI, alongside the positive predictive value (PPV) of discrete scores. AUROC of <0.65 was considered poor, 0.65–0.80 acceptable and >0.80 excellent. Inter-regional analysis used χ^2^ for categorical analysis and one-way analysis of variance for comparing means.

Outcomes were assessed by comparing GOS distribution based on seizure status and a dichotomous approach for poor outcomes comparing GOS=5 against GOS <5; both using χ^2^. Patients were divided into those who did not have an inpatient seizure, those who had an inpatient seizure without SE and those who developed SE.

Bonferroni correction was applied to univariate analysis to limit type I errors, and p values were adjusted with a cap of p>0.99.

R-studio (V.2024.04.0+735) was used for multivariable binary logistic regression on all complete data from the core global dataset. Variables were selected based on univariate analysis OR and evidence from Wood *et al*. The reference categories for the OR for age and aetiology were identical to those used in the Wood *et al* study and are marked as OR 1.00 for clarification. Variables not readily available in all regions, such as EEG, were excluded.^4^ R-studio was used for creating heatmaps of group-associated variables among patients who had inpatient seizures and hierarchical agglomerative cluster analysis. This was performed using the average linkage method and Euclidean distance as the similarity measure. The optimal number of clusters was determined by visual inspection of the dendrogram and assessment with the silhouette plot. The rationale was to compare the analyses done in the original paper and facilitate an exploratory analysis with flexibility, ease of interpretability and the ability to reveal natural groupings.

## Results

**Figure 2:**
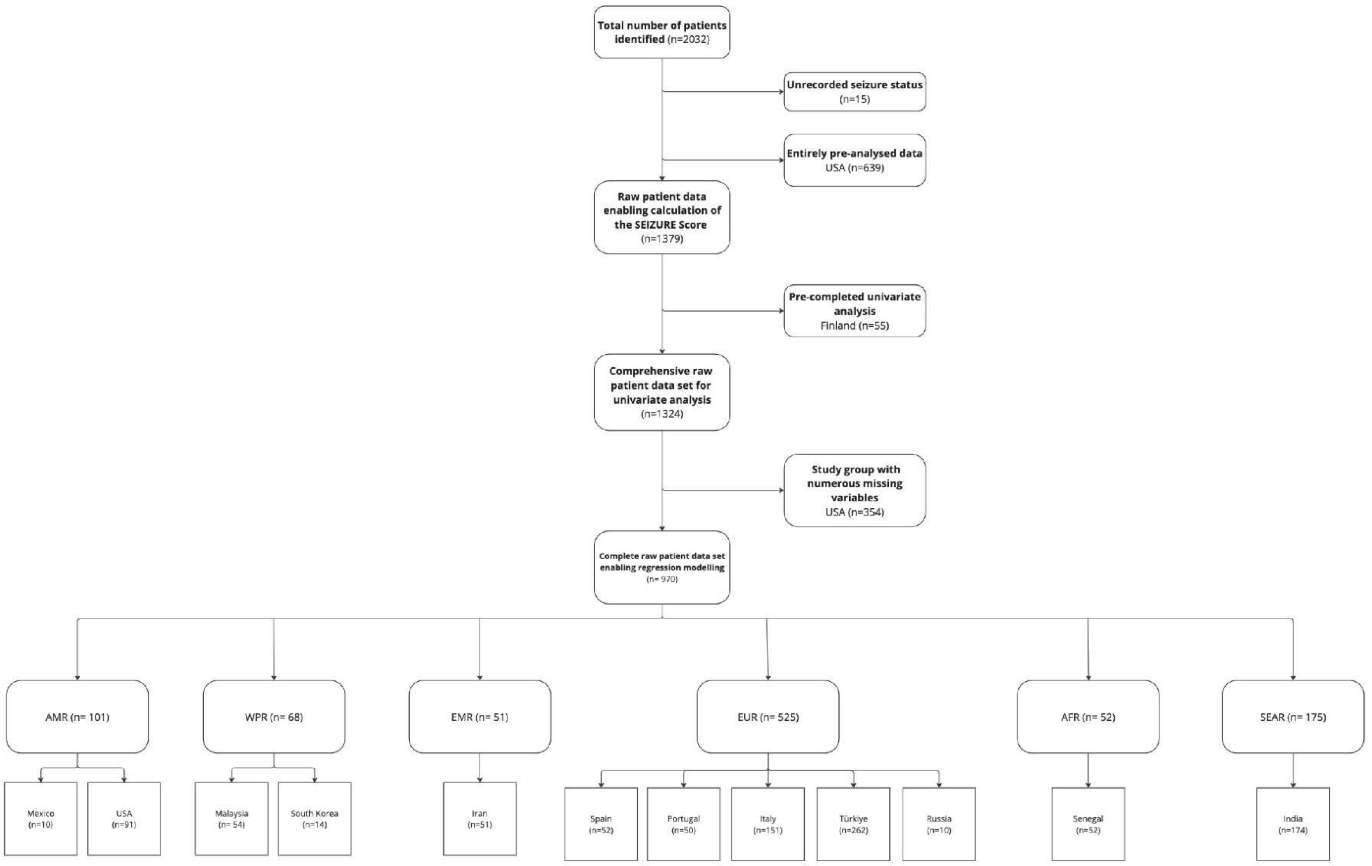
A flow chart detailing the origins of the global patient study group. AFR, African Region; AMR, Regions of the Americas; EMR, Eastern Mediterranean Region; EUR, Europe Region; SEAR, South-East Asia Region; USA, United States of America; WPR, Western Pacific Region.

A total of 2032 patients were identified, of whom 1379 had sufficient raw data to calculate the SEIZURE score, 639 patients were pre-analysed due to ethical approval reasons and 15 were excluded due to missing required data. Within this cohort, univariate analysis was conducted on 1324 patients with comprehensive data and regression modelling was completed on 970 patients with complete raw data (figure 2).

Patients were mostly adults, with fever, seizure and behavioural changes being common presenting complaints. There was a wide range of aetiologies, apart from Africa (AFR), where herpes simplex virus (HSV) (23/52, 44 %) and ‘other’ infections (21/52, 40%) predominated (table 1). Eastern Mediterranean Region (EMR) was mainly HSV infection (13/51, 25.5%), immune-related (11/51, 22%) and unknown causes (13/51, 26%), while South-East Asian Region (SEAR) had high proportions of autoantibody-associated (41/175, 23%) and bacterial encephalitis (38/175, 22%). Western Pacific Region (WPR) was predominantly ‘other’ infections (23/68, 33.8%) and unknown causes (20/68, 29%), while Europe (EUR) and the Americas (AMR) were mostly ‘other’ infections (240/525, 46%) and unknown causes (215/455, 47%), respectively.

**Table 1:** A summary of patient demographics, presentation and investigations separated by WHO region

Variable	Africa (n=52)	Eastern Mediterranean (n=51)	South-East Asia (n=175)	Western Pacific (n=68)	Europe (n=525)	Americas (n=455)
**Demographics**						
Age, years, median (IQR)	43 (28–51.5)	55 (42.5–64.5)	35 (20.5–50)	41 (28–55.5)	55.5 (37–70)	44 (25–63)
Age, years, n (%)						
≤5	0 (0.0)	0 (0.0)	6 (3.4)	0 (0.0)	3 (0.6)	19 (4.2)
>5 to ≤18	0 (0.0)	1 (2)	34 (19.4)	3 (4.4)	10 (1.9)	50 (11.0)
>18 to ≤ 0	22 (42.3)	9 (17.6)	70 (40.0)	30 (44.1)	101 (19.2)	147 (32.3)
>40 to ≤60	19 (36.5)	21 (41.2)	51 (29.1)	19 (27.9)	164 (31.2)	107 (23.5)
>60	11 (21.2)	20 (39.2)	15 (8.6)	16 (23.5)	252 (48.0)	134 (29.5)
Sex, n (%)						
Male	29 (55.8)	18 (35.3)	82 (46.9)	42 (61.8)	301 (57.3)	173 (38.0)
**Aetiology, n (%)**						
Autoantibody-associated	0 (0)	4 (7.8)	41 (23.4)	1 (1.5)	57 (10.9)	118 (25.9)
HSV	23 (44.2)	13 (25.5)	26 (14.9)	0 (0.0)	46 (8.8)	20 (4.4)
VZV	4 (7.7)	3 (5.9)	0 (0.0)	4 (5.9)	19 (3.6)	9 (2.0)
Bacterial	0 (0.0)	2 (3.9)	38 (21.7)	3 (4.4)	36 (6.9)	13 (2.9)
ADEM/immune	0 (0.0)	11 (21.6)	14 (8.0)	7 (10.3)	72 (13.7)	38 (8.4)
Mycobacterium TB	4 (7.7)	0 (0.0)	27 (15.4)	10 (14.7)	3 (0.6)	3 (0.7)
Infection (other)	21 (40.4)	5 (9.8)	21 (12.0)	23 (33.8)	240 (45.7)	38 (8.4)
Unknown	0 (0)	13 (25.5)	8 (4.6)	20 (29.4)	53 (9.9)	215 (47.3)
Immunosuppressed, n (%)	16 (30.8)	12 (23.5)	18 (10.3)	24 (35.3)	61 (11.6)	8 (1.8)
HIV positive, n (%)	9 (17.3)	0 (0.0)	18 (10.3)	6 (8.8)	3 (0.6)	2 (0.4)
**Symptoms**						
Median symptom duration prior to admission, days (IQR, range)	–	4.5 (3–10.75, 1–60)	12 (5–30, 1–365)	3 (2–9.25, 1–60)	2 (1–7, 1–4362)	44.5 (10–266.25, 2–10 024)
GCS at presentation, n/total						
≤8	0/52	3/51	27/175	8/68	83/525	66/455
9–12	5/52	6/51	67/175	8/68	98/525	84/455
13–14	12/52	20/51	45/175	22/68	151/525	101/455
15 (ref)	35/52	22/51	36/175	28/68	195/525	204/455
Temperature over 38°C on admission, n/total	43/52	16/51	23/113	5/67	74/499	57/423
**Presenting symptoms, n (%)**						
Fever	43 (84.3)	25 (49.0)	96 (55.1)	42 (61.8)	189 (36.0)	190 (41.8)
Headache	30 (57.7)	24 (47.1)	109 (62.2)	25 (36.8)	188 (35.8)	36 (35.6)
Lethargy	0 (0.0)	22 (44.0)	96 (54.9)	23 (33.8)	251 (47.8)	20 (19.8)
Seizure	24 (46.2)	20 (40.8)	56 (32.0)	33 (48.5)	194 (38.1)	76 (75.2)
Behavioural change	28 (53.8)	31 (64.6)	145 (82.9)	29 (42.6)	359 (69.0)	50 (49.5)
Focal neurology	29 (55.8)	12 (23.5)	46 (26.2)	15 (22.1)	153 (29.5)	19 (19.0)
Irritability	0 (0.0)	5 (9.8)	56 (32.0)	9 (13.2)	200 (38.9)	23 (22.8)
Neck stiffness	18 (34.6)	6 (11.8)	80 (45.7)	13 (19.1)	146 (28.9)	13 (12.9)
Photophobia	18 (34.6)	3 (5.9)	47 (26.9)	–	45 (11.5)	10 (10.1)
Gastrointestinal	5 (9.6)	6 (11.8)	16 (9.1)	14 (20.9)	47 (9.1)	25 (25.0)
Respiratory	2 (3.8)	3 (5.9)	28 (16.0)	10 (14.7)	40 (7.7)	5 (5.0)
**Diagnostic tests**						
First MRI, n/total						
Abnormal	21/26	36/48	133/169	45/68	223/500	72/100
First EEG, n/total						
Focal abnormality	–	2/27	8/119	9/59	154/466	61/97
Consistent with encephalitis	–	21/27	85/119	36/59	126/466	21/97
Low CSF:serum glucose ratio, n/total	15/51	22/39	66/140	29/61	158/493	24/73
CSF:serum glucose ratio, mean	0.61 (0.27)	0.50 (0.19)	0.53 (0.23)	0.63 (0.24)	0.55 (0.19)	0.58 (0.15)
Low initial serum sodium, n/total	0/52	0/46	5/175	3/68	4/465	0/99
Initial serum sodium, mmol/L, mean	134.1 (4.98)	136.2 (4.67)	135.5 (6.25)	136.4 (6.26)	138.7 (4.74)	137.9 (8.40)
High initial serum lactate, n/total	–	–	25/79	1/18	10/292	12/60
Initial serum lactate, mmol/L, mean	–	–	3.62 (2.14)	2.37 (0.80)	1.56 (2.76)	2.69 (1.33)
Taking anti-seizure medication prior to admission, n/total	0/52	–	5/175	2/55	8/252	22/101

Hyphens indicate missing data.

ADEM, acute disseminating encephalomyelitis; CSF, cerebrospinal fluid; EEG, electroencephalogram; GCS, Glasgow Coma Scale; HSV, herpes simplex virus; TB, tuberculosis; VZV, varicella zoster virus.

Patients that had seizures had significant variation between regions for most variables (online supplemental tables S1 and S2). In particular, significant differences in seizure risk were seen for GCS and autoantibody-associated, HSV and unknown aetiology categories.

Overall, 439 out of 1324 (33.2%) of the patients in the comprehensive raw global dataset had at least one inpatient seizure (table 2). Significant differences were seen in aetiology and the proportion with a GCS ≤8 between patients who did and did not have an inpatient seizure. Autoantibody-associated encephalitis had the highest seizure risk (OR 3.77 (95% CI 2.79 to 5.09)). Data on 13 antibody subtypes were available for 12 out of 221 patients with autoantibody-associated encephalitis. Leucine-rich glioma-inactivated 1, gamma‐aminobutyric‐acid A receptor, Ri, and glutamic acid decarboxylase antibodies all demonstrated significantly increased seizure risks (online supplemental table S3). Presenting with a seizure and having EEG abnormalities was significantly more frequent in those who had an inpatient seizure, while fever, headache, respiratory symptoms and neck stiffness were significantly less common. The variables most significantly related to inpatient seizure risk on multivariable logistic regression (online supplemental table S4) were GCS (OR 0.84 (95% CI 0.78 to 0.91) for each additional point) and seizure being the presenting complaint (OR 49.90 (95% CI 31.68 to 81.02)).

**Table 2:** A summary of the core global dataset separated into patients that did and did not have seizures with univariate analysis and multivariable binary logistic regression of selected variables, additionally stratified by region

Variable	All (n=1324)	Seizure (n=439)	No seizure (n=885)	PPV	NPV	OR (95% CI)	P value (adjusted)	Multivariable binary logistic regression odds ratios (95% CI) (p value)
**Demographics**								
Age, years, median (IQR)	49 (30–66)	50 (29–66)	49 (32–65)				0.39 (>0.99)	
Age, years, n (%)								
≤5	28 (2.1)	7 (1.6)	21 (2.4)	25.0	66.7	0.67 (0.28 to 1.58)	0.43 (>0.99)	0.72 (0.090 to 5.67)
>5 to ≤18	97 (7.3)	37 (8.4)	60 (6.8)	38.1	67.2	1.27 (0.83 to 1.94)		3.98 (1.51 to 10.82)**
>18 to ≤ 40	379 (28.5)	124 (28.2)	252 (28.5)	33.0	66.8	0.99 (0.77 to 1.27)		0.85 (0.49 to 1.48)
>40 to ≤ 60	380 (28.5)	115 (26.2)	263 (29.7)	30.4	65.8	0.84 (0.65 to 1.09)		0.86 (0.51 to 1.45)
>60	448 (33.7)	156 (35.5)	290 (32.8)	35.0	67.8	1.13 (0.89 to 1.44)		1.00 (–)
Sex, n (%)								
Male	644 (48.4)	188 (42.8)	454 (51.3)	29.3	63.2	0.71 (0.56 to 0.90)	**0.0035 (0.11)	0.81 (0.53 to 1.24)
**Aetiology, n (%)**								
Autoantibody-associated	**221 (16.6)**	**129 (29.4)**	**88 (9.9)**	**59.4**	**72.0**	**3.77 (2.79 to 5.09)**	***<0.0001 (**)	1.60 (0.40 to 6.90)
HSV	**129 (9.7)**	**50 (11.4)**	**78 (8.8)**	**39.1**	**67.5**	**1.33 (0.91 to 1.94)**		3.50 (0.86 to 15.37)
VZV	**39 (2.9)**	**6 (1.4)**	**33 (3.7)**	**15.4**	**66.3**	**0.36 (0.15 to 0.86)**		1.00 (–)
Bacterial	**92 (6.9)**	**11 (2.5)**	**81 (9.2)**	**12.0**	**65.3**	**0.26 (0.13 to 0.48)**		2.68 (0.57 to 13.22)
ADEM/immune	**139 (10.4)**	**54 (12.3)**	**84 (9.5)**	**39.1**	**67.5**	**1.34 (0.93 to 1.92)**		1.93 (0.49 to 8.30)
Mycobacterium TB	**47 (3.5)**	**10 (2.3)**	**37 (4.2)**	**21.3**	**66.4**	**0.53 (0.26 to 1.08)**		1.52 (0.28 to 8.50)
Infection (other)	**351 (26.4)**	**96 (21.9)**	**255 (28.8)**	**27.4**	**64.7**	**0.69 (0.53 to 0.90)**		1.73 (0.47 to 6.91)
Unknown	**309 (23.2)**	**81 (18.5)**	**227 (25.6)**	**26.3**	**64.8**	**0.66 (0.49 to 0.87)**		1.28 (0.31 to 5.69)
Immunosuppressed, n (%)	139 (10.4)	50 (11.4)	89 (10.1)	36.0	67.2	1.15 (0.80 to 1.66)	0.46 (>0.99)	
HIV positive, n (%)	38 (2.9)	7 (1.6)	31 (3.5)	18.4	66.4	0.45 (0.19 to 1.02)	0.055 (>0.99)	
**Symptoms**								
Median symptom duration, days (IQR, range)	5 (2–17, 1–10 024)	2 (1.5–17.5, 1–10 024)	2 (2–15, 1–4988)				**0.0069 (0.16)	
GCS at presentation, n/total								Per point 0.84 (0.78 to 0.91) (***)
≤8	**188/1331**	**83/439**	**104/885**	**44.4**	**68.7**	**1.87 (1.33 to 2.63)**	***0.0004 (***)	
9–12	268/1331	89/439	178/885	33.3	66.9	1.17 (0.85 to 1.61)	0.32 (>0.99)	
13–14	351/1331	112/439	239/885	31.9	66.4	1.1 (0.82 to 1.47)	0.52 (>0.99)	
15 (ref)	520/1331	155/439	364/885	29.9	64.7	1.00 (–)	1.00 (1.00)	
Temperature over 38°C on admission, n/total	227/1156	72/386	155/770	31.7	66.3	0.91 (0.67 to 1.24)	0.50 (>0.99)	0.88 (0.49 to 1.57)
**Presenting symptoms, n (%)**								
Fever	**584 (44.2)**	**159 (36.2)**	**423 (50.0)**	**27.3**	**62.1**	**0.61 (0.49 to 0.78)**	***<0.0001 (***)	0.87 (0.52 to 1.44)
Headache	**411 (42.9)**	**117 (33.7)**	**292 (47.8)**	**28.6**	**58.1**	**0.56 (0.42 to 0.73)**	***<0·0001 (***)	
Lethargy	411 (44.8)	152 (45.6)	258 (44.2)	37.1	64.3	1.06 (0.81 to 1.39)	0.68 (>0.99)	
Seizure	**400 (42.0)**	**306 (88.7)**	**87 (14.3)**	**77.9**	**93**	**46.90 (31.33 to 70.19)**	***<0·0001 (***)	49.90 (31.68 to 81.02) (***)
Behavioural change	641 (66.6)	216 (61.7)	423 (69.1)	33.8	58.5	0.72 (0.55 to 0.95)	*0.023 (0.64)	
Focal neurology	275 (28.5)	96 (27.3)	177 (28.9)	35.2	63	0.92 (0.69 to 1.23)	0.60 (>0.99)	
Irritability	293 (32.3)	91 (28.1)	200 (34.3)	31.3	62.2	0.75 (0.56 to 1.01)	0.063 (>0.99)	
Neck stiffness	**277 (29.1)**	**78 (22.5)**	**199 (32.9)**	**28.2**	**60.2**	**0.59 (0.44 to 0.80)**	***<0·0001 (*)	
Photophobia	113 (16.9)	31 (14.7)	82 (18.0)	27.4	67.5	0.79 (0.50 to 1.23)	0.32 (>0.99)	
Gastrointestinal	114 (11.9)	42 (11.9)	72 (11.9)	36.8	63.3	1.00 (0.67 to 1.50)	>0.99 (>0.99)	
Respiratory	**87 (9.0)**	**17 (4.8)**	**70 (11.5)**	**19.5**	**61.5**	**0.39 (0.22 to 0.67)**	***0.0004 (*)	
**Diagnostic tests**								
First MRI, n/total								
Abnormal	529/909	202/334	323/575	38.5	65.6	1.19 (0.91 to 1.57)	0.21 (>0.99)	
First EEG, n/total								
Focal abnormality	**232/767**	**175/313**	**55/454**	**76.1**	**74.3**	**32.83 (19.28 to 55.90)**	***<0·0001 (***)	
Consistent with encephalitis	**290/767**	**116/313**	**172/454**	**40.3**	**58.9**	**6.96 (4.23 to 11.44)**	***<0·0001 (***)	
Low CSF:serum glucose ratio, n/total	**303/858**	**84/306**	**218/552**	**27.8**	**60.1**	**0.58 (0.43 to 0.79)**	***0·0004 (***)	
CSF:serum glucose ratio, mean (SD)	**0.56 (0.19)**	**0.58 (0.16)**	**0.54 (0.20)**	**–**	**–**	**–**	**0.0014 (*)	
Low initial serum sodium, n/total	11/906	4/324	7/582	36.4	64.2	1.03 (0.30 to 3.53)	>0.99 (>0.99)	
Initial serum sodium, mmol/L, mean (SD)	137.6 (6.15)	137.8 (5.95)	137.5 (6.28)				0.34 (>0.99)	
High initial serum lactate, n/total	48/451	21/141	27/310	43.8	70.2	1.83 (1.00 to 3.37)	0·0069 (>0.99)	
Initial serum lactate, mmol/L, mean (SD)	2.11 (1.90)	2.48 (2.40)	1.95 (1.61)				**<0.01 (0.19)	
Taking anti-seizure meds on admission, n/total	**40/682**	**26/266**	**14/416**	**65.0**	**62.6**	**3.11 (1.59 to 6.07)**	***<0.001 (*)	1.02 (0.48 to 2.20)

*p<0.05; **p<0.01; ***p<0.001. Bold font highlights significant differences after Bonferroni correction, adjusted p values or level of significance are shown in parentheses. χ2 and Fisher’s exact tests were used for univariate categorical variable analysis and Student’s t-test was used for testing of continuous variables. ORs in multivariable binary logistic regression that were not significant do not have the associated p values included.

GCS was treated as an categorical variable for univariate analysis and a continuous variable for multivariable analysis.

ADEM, acute disseminated encephalomyelitis; CSF, cerebrospinal fluid; EEG, electroencephalogram; GCS, Glasgow Coma Scale; HSV, herpes simplex virus; NPV, negative predictive value; PPV, positive predictive value; TB, tuberculosis; VZV, varicella zoster virus.

**Figure 3:**
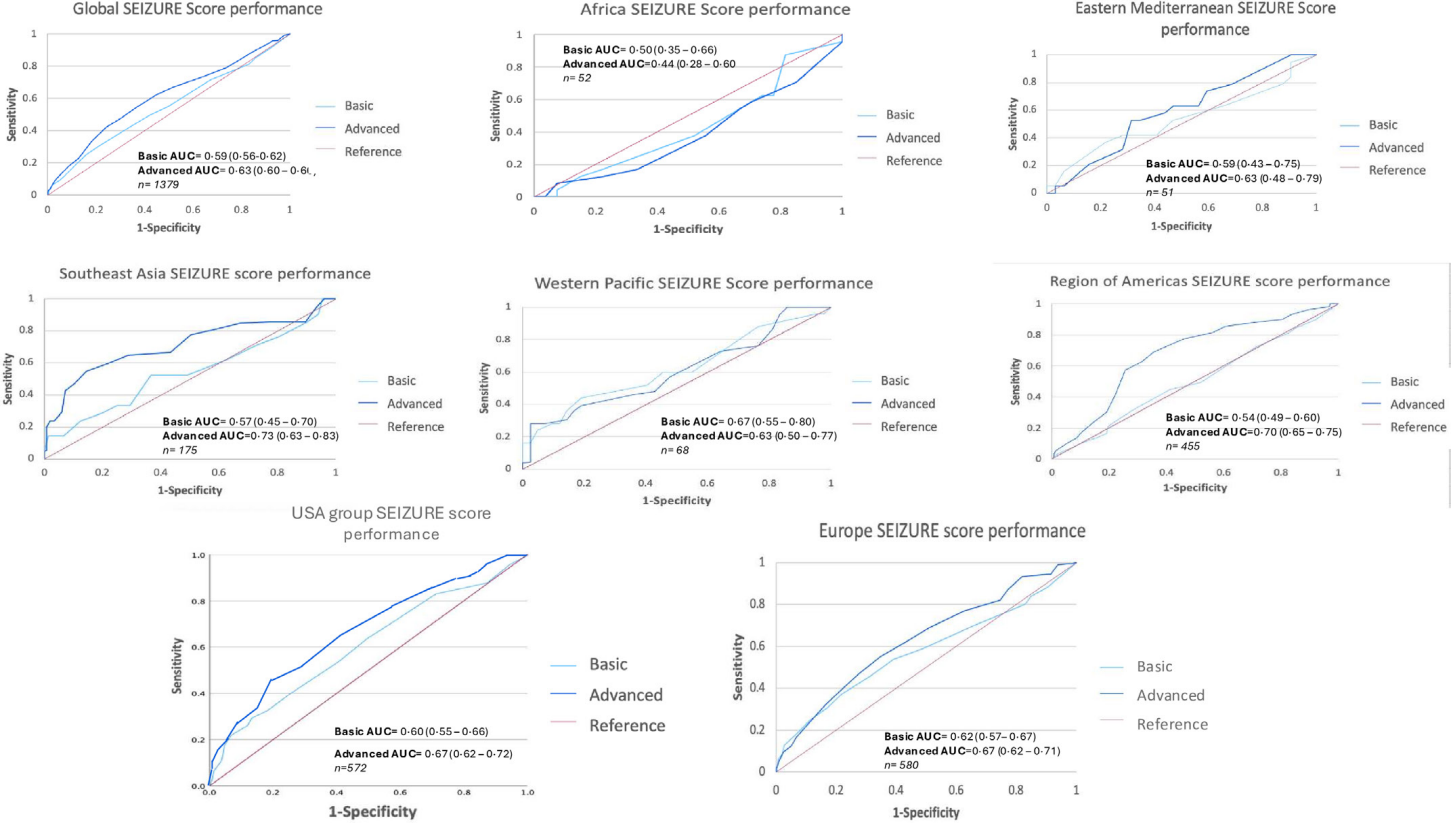
Receiver operating curves (ROC) for the performance of the SEIZURE (SEIZUre Risk in Encephalitis) score for the global study group and all WHO regions with area under the curve calculated and sample sizes documented. AUROC, area under the receiver operating curve; USA, United States of America.

Combining the cohorts overall, the scoring system performed poorly with a basic score AUROC of 0.58 (95% CI 0.55 to 0.62) and an advanced score AUROC of 0.63 (95% CI 0.60 to 0.66). The advanced score performed better in AMR and SEAR (AUROC 0.70 (95% CI 0.65 to 0.75) and 0.73 (95%CI 0.63 to 0.83), respectively) (figure 3). Additionally, the greater the basic and advanced SEIZURE score, the higher the rates of inpatient seizures in the global cohort (OR 2.07 (95% CI 1.37 to 3.08) and OR 2.72 (95% CI 2.03 to 3.60), respectively), although this was predominantly due to European and USA cohorts (online supplemental table S5). A PPV of over 0.50 was observed in the basic score for Africa (scores 9–11), Eastern Mediterranean (9–11) and the highest scoring category in Western Pacific, USA group and Europe. In the advanced scoring system, a PPV >0.50 was also calculated in Eastern Mediterranean (scores 14–16) and the highest scoring categories of Western Pacific, Region of the Americas, USA group and Europe. After Bonferroni correction in these groups, statistical significance was observed only in Europe, USA group and Region of the Americas.

### Africa

All patients were adults and 24 out of 52 (46%) had a seizure. Every patient presenting with a seizure had a further inpatient seizure (online supplemental table S6). Aetiology was significantly different prior to adjustment. EEG and serum lactate were not conducted.

SE occurred in 8 out of 24 patients, mostly due to HSV-1 (4/8, 50%). All patients had an abnormal MRI and 6 out of 8 (75%) died.

The SEIZURE score in AFR demonstrated poor discriminatory ability with an AUROC of 0.50 (95% CI 0.35 to 0.66) and 0.44 (95% CI 0.28 to 0.60) for basic and advanced scores, respectively. No significant differences were seen in discrete scoring categories.

### Eastern Mediterranean

Within EMR, 19 out of 51 (37%) patients had a seizure with no significant differences in demographics (online supplemental table S7). HSV encephalitis was associated with a high seizure risk (OR 3.93 (95% CI 1.05 to 14.69)) and patients presenting with a seizure had a significantly increased risk of a subsequent inpatient seizure (OR 54.00 (95% CI 8.87 to 328.82)).

SE occurred in 5 out of 51 patients (10%), mostly those with unknown aetiology. All patients with SE had an abnormal MRI and an EEG consistent with encephalitis, of whom 4 out of 5 (80%) presented with a seizure, and 2 out of 5 (40%) died.

Within the EMR cohort, there was poor discriminatory ability of the basic SEIZURE score (AUROC 0.59 (95% CI 0.43 to 0.75)), which slightly improved for the advanced score (0.63 (95% CI 0.48 to 0.79)).

### South-East Asia

Of the SEAR cohort, 21 out of 175 (12%) had a seizure and age was associated with significant differences in inpatient seizure risk (online supplemental table S8). Autoantibody-associated encephalitis carried the highest seizure risk of aetiologies (OR 5.75 (95% CI 2.21 to 14.92)). Significant differences in inpatient seizure risk were seen in patients presenting with a seizure (OR 9.12 (95% CI 3.14 to 26.50), p<0.01) and, prior to adjustment, patients with GCS ≤8 (OR 5.85 (95% CI 1.23 to 29.67)).

Seven patients developed SE and all survived. All but one had autoantibody-associated encephalitis, abnormal MRIs and EEGs consistent with encephalitis, and presented with a seizure.

The basic score had a poor AUROC of 0.57 (95% CI 0.45 to 0.70) and adding aetiology improved the AUROC to 0.73 (95% CI 0.63 to 0.83).

### Western Pacific

There were no significant differences in demographics, GCS, investigations or aetiology between those with and without inpatient seizures (online supplemental table S9). All the inpatient seizure group presented with a seizure, while gastrointestinal and respiratory complaints were negatively associated with seizure risk.

Of this cohort, 3 out of 25 (12%) developed SE, all with ‘other’ infections or unknown aetiology, and all patients survived.

The SEIZURE score showed acceptable discrimination in WPR. The AUROC for the basic and advanced scores was 0.67 (95% CI 0.55 to 0.80) and 0.63 (95% CI 0.50 to 0.77), respectively. Prior to adjustment, a significant increase in seizures was seen in patients scoring over 12 in the basic score (OR 6.00 (95% CI 1.14 to 31.29)).

### Europe

The overall EUR group consisted of 580 patients, of whom raw data were available for 525 (online supplemental table S10). Finland sent pre-calculated scores for 55 patients to incorporate into EUR. The online supplemental file contains individual country data.

Of those with raw data available, 201 out of 525 (38%) had a seizure and autoantibody-associated and HSV encephalitis demonstrated increased seizure risk (OR 2.39 (95% CI 1.35 to 4.23) and 2.38 (95% CI 1.28 to 4.43)), respectively, while bacterial (OR 0.37 (95%CI 0.16 to 0.85)) and ‘other’ infections (OR 0.59 (95% CI 0.41 to 0.85)) were associated with reduced risk. The clinical factors most strongly associated with inpatient seizure risk were presenting with a seizure (OR 169.75 (95% CI 82.24 to 338.03)) and GCS ≤8 (OR 3.18 (95% CI 1.84 to 5.46)). EEG changes and high lactate measurements were also associated with inpatient seizures.

SE occurred in 58 out of 503 (12%), frequently due to autoantibody-associated or other immunological aetiologies, and 3 out of 58 (5%) died. Fever was present in 20 out of 58 (34%) and 41 out of 58 (71%) presented with a seizure. In addition, 37 out of 58 (65%) had an abnormal MRI and 48 out of 50 (96%) had an abnormal EEG.

The basic score performed poorly in Europe with an AUROC of 0.62 (95% CI 0.57 to 0.67) and an improved, acceptable, advanced score AUROC of 0.67 (95% CI 0.62 to 0.71). The highest scoring categories were associated with significantly increased seizure rates.

ROC for individual EUR countries is available in online supplemental tables S11–16, figure S1. Excluding Spain, the SEIZURE score performed acceptably within western Europe. The highest discrimination was in Portugal, with an excellent basic score AUROC of 0.82 (95% CI 0.69 to 0.94) and 0.83 (95% CI 0.72 to 0.95) for the advanced score. In Spain, the basic score AUROC was 0.62 (95%CI 0.44 to 0.79) and the advanced score was 0.54 (95% CI 0.36 to 0.73).

### The Americas

The AMR cohort included 1094 patients, of whom 639 were pre-analysed as ‘USA group’ (online supplemental table S17). The AMR ROC and tables refer to the remaining 455, including partial data from 354 patients (online supplemental table S18).

The USA group had a high proportion of unknown aetiology (236/620, 38%) with significant differences in inpatient seizure risk seen in known aetiologies. The highest risk of an inpatient seizure was presenting with a seizure (OR 11.03 (95% CI 7.38 to 16.37)).

In AMR, 152 out of 455 (33%) had an inpatient seizure with a significantly lower risk in males (OR 0.20 (95% CI 0.14 to 0.30)). A significant difference was seen between aetiologies, with the highest risk in those with an autoantibody cause (OR 2.98 (95% CI 1.93 to 4.62)) while fever was significantly associated with reduced seizure risk (OR 0.46 (95% CI 0.30 to 0.69)).

SE occurred in 36 out of 99 (36%) patients with complete data in AMR and 3 out of 36 (9%) patients died. Within this group, 21 out of 36 (58%) had autoantibody-associated encephalitis and 13 out of 36 (36%) other immune encephalitides. Overall, 31 out of 99 (86%) presented with a seizure, while abnormal MRI and EEG were seen in 25 out of 36 (69%) and 33 out of 36 (92%) of cases, respectively.

The SEIZURE score had variable discriminatory ability with a poor basic AUROC of 0.60 (95% CI 0.55 to 0.66) and 0.54 (95% CI 0.49–0.60) for USA group and AMR, respectively. The advanced score AUROC improved to 0.67 (95% CI 0.61–0.72) and 0.70 (95% CI 0.65–0.75), respectively. Importantly, the higher the SEIZURE score, the greater the seizure risk in both groups.

### Hierarchical cluster analysis

**Figure 4:**
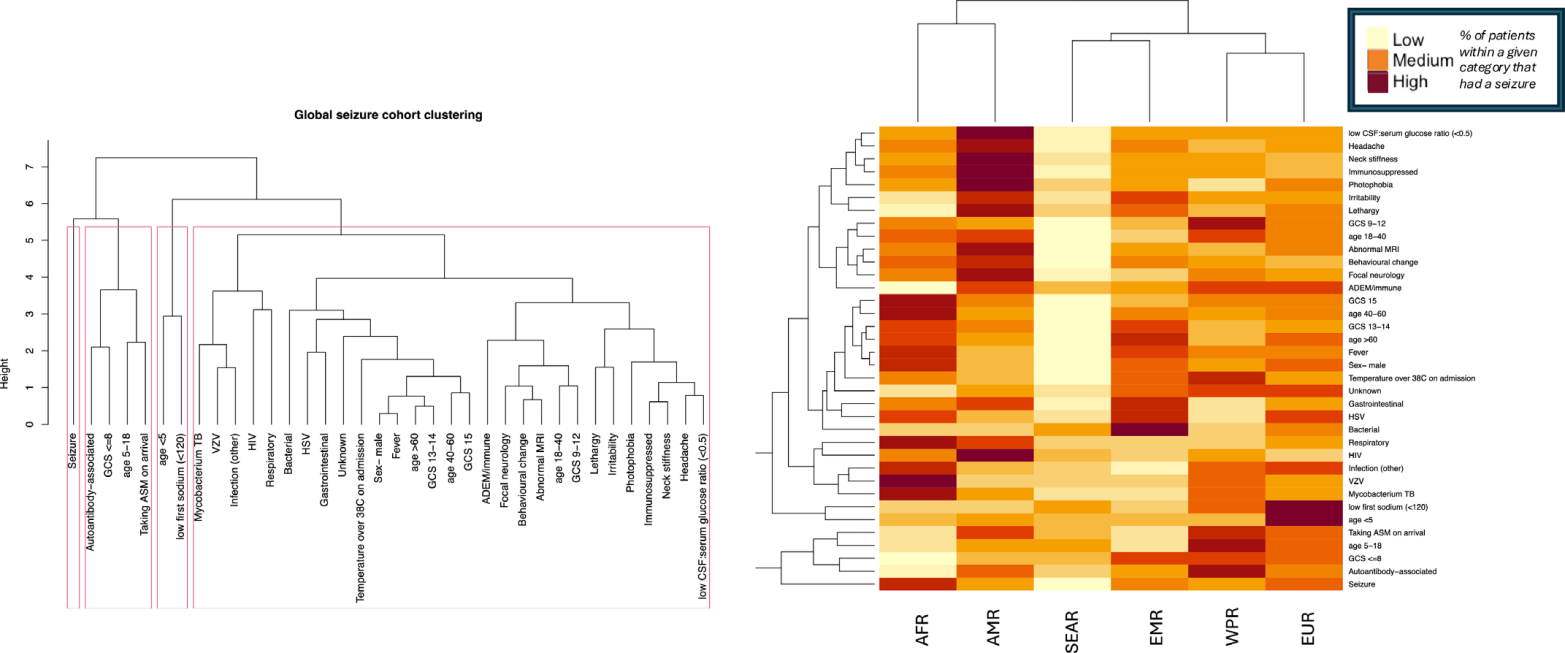
A diagram of complete hierarchical cluster analysis and the associated heatmap demonstrating associated variables among patients that had seizures in the global cohort. Red boxes delineate the variables into four appropriate clusters. ADEM, acute disseminated encephalomyelitis; AFR, Region of Africa; AMR, Region of the Americas; ASM, anti-seizure medication; CSF, cerebrospinal fluid; EMR, Eastern Mediterranean region; EUR, European region; GCS, Glasgow Coma Scale; HSV, herpes simplex virus; SEAR, South-East Asian region; TB, *Mycobacterium tuberculosis*; VZV, varicella zoster virus (VZV); WPR, Western Pacific region.

Hierarchical clustering assessed for associated variables among patients that had inpatient seizures (figure 4). The first two clusters are markedly separate from most other variables and contain high-risk variables including presenting with a seizure, autoantibody-associated aetiology, GCS ≤8, age 5–18, and taking ASM prior to admission. The heatmap demonstrates the percentage of patients that had an inpatient seizure, demonstrating wide heterogeneity between regions.

Separate EUR analysis (online supplemental figure S2) showed a similar cluster including GCS ≤8, presenting with a seizure, and focal EEG abnormalities. The third cluster contained low seizure-risk variables including VZV and GCS 15.

### Outcomes

**Figure 5:**
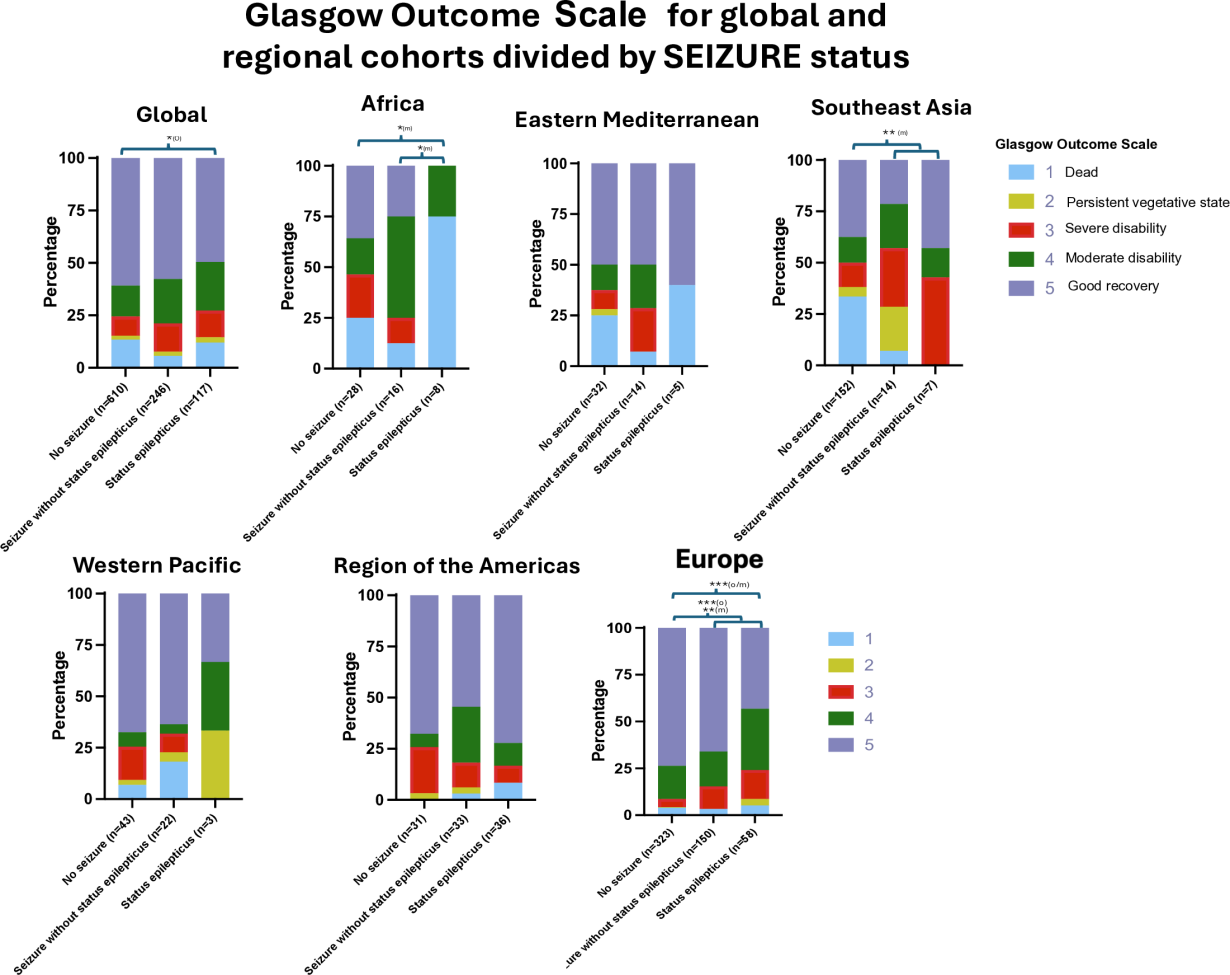
Bar charts of the global and regional study groups showing the distribution of Glasgow Outcome Scale scores when separated dependent on seizure status (no inpatient seizure, inpatient seizure without status epilepticus, status epilepticus). (**o**) signifies a significant difference in the proportion of poor outcomes; (**m**) signifies significantly different distributions of Glasgow Outcome Scale scores between groups. *p<0.05; **p<0.01; ***p<0.001.

Outcomes varied between regions (figure 5). Globally, individuals with SE were significantly more likely to have poor outcomes than patients who did not have seizures (OR 1.8 (95% CI 1.07 to 2.34), p=0·03). The EUR cohort demonstrated significant differences in poor outcomes in patients who developed SE as compared with those who patients did not have inpatient seizures (OR 3.70 (95% CI 2.09 to 6.64)). GOS distributions between patients who did and did not have seizures differed significantly in SEAR, without a significant difference in proportion with a poor outcome. In the AFR cohort, GOS distributions differed significantly between patients that developed SE and patients that had no seizure or a seizure without SE No differences in GOS were seen in the AMR, EMR and WPR cohorts.

## Discussion

Encephalitis carries a high seizure risk associated with worse outcomes, as shown in our analysis.^1^ Our study used the SEIZURE score, consisting of age, GCS, fever and aetiology, to assess its potential for seizure prediction in encephalitis patients internationally. The score showed acceptable discrimination in western Europe, excluding Spain, and identified high-risk patients in AMR. However, even in these areas, the results suggest that it has a limited clinical utility in its current format. The poorer performance elsewhere was likely partly due to underpowered cohorts, particularly the proportion with a SEIZURE score >12, and differing epidemiology, confirming that many regions require localised scoring systems. This study highlighted the large proportion of unknown encephalitis cases and heterogeneity of seizure risk for given aetiologies.

Hierarchical analysis identified high-risk variables including prior seizures, autoantibody-associated aetiology and low GCS, many of which were significant after logistic regression. These findings align with prior literature and aspects were reflected in groups with good SEIZURE score discrimination.^216^,^18^ Finland demonstrated limited improvement with aetiology, likely due to few autoantibody-associated patients having seizures. This is despite high seizure rates among patients with HSV encephalitis, a documented epileptogenic aetiology.^19^ Poor performance in Spain was likely influenced by a low number of normal GCS patients, reducing analytical power.

The ability to analyse regional heterogeneity was limited by wide inter-regional variations in seizure rates, even within the same encephalitis aetiology. This could explain the poor performance of the SEIZURE score globally. Variance may stem from symptom duration, time until management, availability of neurology expertise and EEG access, particularly for subtle motor or non-convulsive seizures.^20^ The necessity for timely treatment is well documented, particularly for HSV and *Mycobacterium tuberculosis* encephalitis, which both had marked inter-regional variation.^21^,^23^ To better analyse regional heterogeneity, future studies should specify whether inpatient seizures were diagnosed clinically or via EEG, detailing durations of EEG recording in regions with readily-available monitoring. Additionally, ASM use differed between centres, something shown in evident international studies.^19 24^ Some centres prescribed ASM prophylactically at the clinician’s discretion for patients presenting with seizures in the community. This may explain regions where seizure risk was unchanged irrespective of GCS, but the timepoints of ASM prescribing are unknown, preventing subanalysis.

Second, a single SEIZURE score for unknown or ‘other’ infections is not applicable globally. Regional encephalitis aetiology varies with rarer ‘other’ infections in the UK being common elsewhere, such as Japanese encephalitis virus in WPR.^25^ This may be partly reflected in diagnostic testing capacity, yet variance in the proportion of encephalitis of unknown aetiology differed widely between countries despite similar diagnostics.^26^ Furthermore, seizure risk differs within the unknown category, with Glaser *et al* and other studies describing clinical subsets.^27 28^ This is reflected among our Portugal and Spain cohorts, with seizure rates for ‘other’ infections of 28.6% and 0%, respectively.

Our study did not associate fever with increased seizure risk, likely due to the higher proportion of bacterial (meningo)encephalitis cases. The negative association between seizure risk and neurological signs/symptoms including neck stiffness and headache in some of our groups suggests predominantly meningeal, as opposed to parenchymal, involvement which carries a lower seizure risk.^29 30^ Fever, including subjective or measured fevers, was associated with a significantly lower seizure risk globally. This suggests fever may indicate the underlying aetiology which confounds the results, as opposed to independently increasing seizure risk. In the original UK study, fever may have been linked to the high HSV prevalence, while in this study, it may indicate lower seizure-risk viruses, such as VZV. Additionally, autoantibody-associated encephalitis can manifest as seizures with apyrexia, which may further explain this negative association.^9^ Indeed, a fever may not be detrimental; a meta-analysis found moderate fever was associated with improved outcomes for some infectious diseases.^31^ Fever is an established seizure risk factor in children. The relatively low proportion of children, compared with Wood *et al,* may have underpowered our study.^7^ This would explain why age was often not a significant risk factor despite prior literature.^2 5^ Nonetheless, our findings suggest that the link between seizure risk and fever or bacterial encephalitis needs further evaluation.

Our results highlight the global treatment gap for encephalitis and seizures, with particularly poor outcomes in AFR and SEAR, where the disease burden is likely highest. These regions had fewer EEG data, which limits the diagnosis of subtle seizures. However, in our global cohort, EEG abnormalities carried a higher seizure risk than a high SEIZURE score, highlighting the need to improve global EEG access.^5^ The WHO recently identified encephalitis as a public health priority, stating the need for further research in low- and middle-income countries and for a One Health approach.^32^ The WHO Intersectoral Global Action Plan on Epilepsy and Other Neurological Disorders provides a framework for further action.

This study represents global collaboration within the neurology community, with one of the largest sample sizes of any encephalitis study. The case report form provided ensured standardisation with Wood *et al* and minimised inter-centre variability. Retrospective data collection facilitated maximising the sample size, but may have introduced misclassification bias, particularly for aetiology. This was mitigated by minimising the amount of ‘mandatory’ variables to ensure the data to calculate the SEIZURE score were accurate. Additionally, this reduced the number of participants excluded from the study due to key missing data. While this negated the need for multiple imputation analysis, this may be beneficial in future large-scale studies with higher proportions of participants with clinically important missing data. The study involved countries from every WHO region and thus available resources were highly varied. Furthermore, timely access to qualified personnel for interpretation of MRI and EEGs may have contributed to the regional differences. All centres had access to similar standard diagnostic tools, yet some centres did not routinely run specialised tests. This variance, and the inclusion of patients from a decade ago prior to rigorous antibody testing in some settings, may have inflated the unknown proportions.^33^ To maintain statistical power, we ensured each region contained at least 50 patients. However, only having one or two countries in certain regions, with minimal children, impacts the ability to draw comprehensive conclusions.

Despite many of the countries in Western Europe performing better than other regions, the poor performance in Spain warrants caution prior to widespread use and in many areas, including the Americas, the discrimination ability only just reached ‘acceptable’. Furthermore, when creating scoring categories, globally and across many of the regions, the scores had a low PPV of <0.50, suggesting limited reliability in predicting the risk of an inpatient seizure. Although the PPV was often >0.50 when tested on high scoring patients in Europe and the USA, the general trend of lower PPVs globally suggests the score may be better used as a screening tool to highlight patients who may benefit from closer monitoring as opposed to a confirmatory test of seizure risk. While the overall cohort number was large, the inclusion of a single centre or country is unlikely to be representative of the entire region. Therefore, some of the regional analysis was likely underpowered and could be evaluated with larger sample sizes prior to reliably concluding the lack of discriminatory ability. Future research should focus on prospective trials and refining the scoring system within Western Europe. Bespoke scoring systems should be created for other regions with larger regional sample sizes and standardised case ascertainment methodology. The scoring allocation for fever and bacterial encephalitis should be reassessed alongside the single score for unknown infections, and aetiological categories should be region-specific. Additional variables could be included, particularly presenting with a seizure and EEG abnormalities for regions with reliable access, both of which carried a higher risk of having an inpatient seizure than patients in the highest category of the SEIZURE score in the global cohort. Conversely, negative predictors could be included, such as respiratory symptoms. Ultimately, regions that can demonstrate a localised scoring system with good discriminatory ability in prospective trials should aim to conduct randomised trials of ASM prophylaxis in patients with encephalitis deemed at high risk of seizures.

## Conclusion

The SEIZURE score performed variably across international settings, with the best performance observed in several Western European countries. However, in most regions and countries, the scoring system fell below the threshold typically considered clinically useful. Even in areas where the performance was acceptable, prospective validation trials are still needed. Other regions should develop local scoring systems with larger cohorts to improve the discriminatory ability. This will facilitate future trials on the utility of ASM prophylaxis and screening for high-risk patients to target further investigations.

## Supplementary material

10.1136/bmjopen-2025-099451 online supplemental file 1

## Data Availability

Data are available upon reasonable request.
