# Supplementary material for: International evaluation of the SEIZUre Risk in Encephalitis (SEIZURE) score for predicting acute seizure risk
Source: BMJ Open. 2025 Dec 7;15(12):e099451. doi: 10.1136/bmjopen-2025-099451 (PMC12699598; doi:10.1136/bmjopen-2025-099451)
Supplement: online supplemental file 1 [file bmjopen-15-12-s001.docx]

**Supplementary data**

Contents

Page:

1. Contents

2-3 Table S1: Analysis between WHO regions of patients that had seizures

4-5 Table S2: Analysis between European countries of patients that had seizures

6. Table S3: A summary table of the seizure risk for all autoantibody subtypes identified in the global cohort

7-8 Table S4: Multivariate analysis

9-10 Table S5: A summary of the basic and advanced SEIZURE score performances when separated into discrete categories

11-13 Table S6: Africa cohort

14-16 Table S7: Eastern Mediterranean cohort

17-19 Table S8: South-East Asian cohort

20-22 Table S9:Western Pacific cohort

23-25 Table S10: Europe cohort

26. Figure S1: European Receiver Operating Curves

27-29 Table S11: Italy cohort

30-32 Table S12: Spain cohort

33-35 Table S13: Portugal cohort

36-38 Table S14: Türkiye cohort

39-42 Table S15: Finland cohort

43-45 Table S16: Russia cohort

46. Table S17: USA study group

47-49 Table S18: Americas cohort

50. Figure S2: European cluster analysis with heat map

| **Variable** | **Eastern Mediterranean**  **(n=19)** | **Africa (n=24)** | **South-East Asia (n=21)** | **Western Pacific (n=25)** | **Region of the Americas (n=152)** | **Europe (n=201)** | **p-value** | **Adjusted p-value** |
| --- | --- | --- | --- | --- | --- | --- | --- | --- |
| **Demographics** |  |  |  |  |  |  |  |  |
| Age, years, mean (SD) | 58·89 (14·07) | 46·38 (18·40) | 21·04 (16·80) | 39·28 (19·68) | 41·80 (21·87) | 55·99 (19·47) | ***** <0·0001** | ****<0·0025** |
| Age, years, patients that had a seizure (%)  ≤5 | - | - | 0 (0) | - | 4 (21) | 3 (100) | 0·023 | 0·58 |
| <5 to ≤18 | 0 (0) | - | 12 (35) | 3 (100) | 17 (34) | 6 (60) | 0·066 | >0·99 |
| <18 to ≤40 | 2 (22) | 8 (36) | 7 (10) | 13 (43) | 62 (42) | 33( 33) | *****<0·0001** | ****<0·0025** |
| <40 to ≤60 | 7 (33) | 11 (58) | 1 (2) | 5 (26) | 31 (29) | 61 (37) | ***** <0·0001** | ****<0·0025** |
| >60 | 10 (50) | 5 (46) | 1 (7) | 4 (25) | 38 (28) | 98 (36) | * 0·023 | 0·58 |
| Sex, years, patients that had a seizure (%)  Male | 7 (39) | 13 (45) | 7 (9) | 13 (31) | 41 (24) | 109 (36) | ***** <0·0001** | ****<0·0025** |
| **Observations on arrival,** patients that had a seizure (%) | |  |  |  |  |  |  |  |
| Temperature over 38°C | 8 (50) | 18 (42) | 0 (0) | 3 (60) | 16 (28) | 27 (32) | ** 0·0038 | 0·095 |
| GCS ≤8 | 2 (67) | - | 7 (26) | 5 (63) | 21 (32) | 48 (58) | ** 0·0027 | 0·068 |
| 9-12 | 2 (33) | 2 (40) | 8 (12) | 5 (63) | 31 (37) | 41 (42) | ***** 0·0008** | ****0·002** |
| 13-14 | 10 (50) | 6 (50) | 4 (9) | 6 (27) | 36 (36) | 50 (33) | ** 0·0050 | 0·13 |
| 15 | 5 (23) | 16 (46) | 2 (6) | 9 (30) | 64 (31) | 59 (30) | **0·0040 | 0·1 |
| **Aetiology,** patients that had a seizure (%) | |  |  |  |  |  |  |  |
| Antibody-associated | 2 (50) | - | 12 (29) | 1 (100) | 83 (70) | 31 (54) | ***** <0·0001** | ****<0·0025** |
| HSV | 8 (62) | 12 (52) | 1 (4) | - | 3 (15) | 26 (57) | ***** <0·0001** | ****<0·0025** |
| VZV | 0 (0) | 2 (50) | - | 1 (25) | 0 (0) | 3 (16) | 0·18 | >0·99 |
| ADEM/immune | 3 (27) | - | 3 (21) | 3 (43) | 17 (45) | 31 (43) | 0·48 | >0·99 |
| *M.tuberculosis* | - | 3 (75) | 0 (0) | 5 (50) | 1 (33) | 1 (33) | ***** <0·0001** | ****<0·0025** |
| Bacterial | 1 (50) | - | 3 (8) | 0 (0) | 0 (0) | 7 (19) | 0·091 | >0·99 |
| Other | - | 7 (33) | 2 (10) | 6 (26) | 5 (13) | 76 (32) | * 0·036 | 0·90 |
| Unknown | 5 (39) | - | 0 (0) | 9 (45) | 43 (20) | 24 (45) | ***** <0·0001** | ****<0·0025** |
| **Inpatient tests,** patients that had a seizure (%) |  |  |  |  |  |  |  |  |
| MRI abnormal | 15 (42) | 9 (42) | 15 (11) | 16 (36) | 50 (69) | 99 (4) | ***** <0·0001** | ****<0·0025** |
| EEG focal abnormality | 2 (100) | - | 2 (25) | 5 (56) | 47 (77) | 121 (79) | **** 0·0059** | 0·15 |
| EEG consistent with encephalitis | 14 (67) | - | 11 (13) | 16 (44) | 15 (71) | 60 (48) | ***** <0·0001** | ****<0·0025** |
| Low CSF:serum glucose | 7 (32) | 4 (27) | 2 (3) | 5 (29) | 18 (75) | 48 (30) | ***** <0·0001** | ****<0·0025** |
| High lactate | - | - | 1 (4) | 0 (0) | 12 (100) | 8 (80) | ***** <0·0001** | ****<0·0025** |

*Table S1-* A table detailing the percentage of patients that had a seizure within each variable for each WHO region with univariate analysis.

Statistical analysis completed using analysis of one-way variance and χ^2^ or Fisher’s exact tests (significance levels determined as * p<0·05; ** p<0·01; *** p<0·001)

Bold font highlight significant differences after Bonferroni correction, shown as adjusted p-values.

For variables with no patients, regardless of seizure status, (-) was used in place of 0.

*Abbreviations: acute disseminating encephalomyelitis (ADEM), cerebrospinal fluid (CSF), electroencephalogram (EEG), Glasgow Coma Scale (GCS), Herpes simplex virus (HSV),magnetic resonance imaging (MRI), Mycobacterium tuberculosis (M.tuberculosis), standard deviation (SD), Varicella zoster virus (VZV)*

| **Variable** | **Italy** | **Portugal** | **Spain** | **Türkiye** | **p-value** | **Adjusted p-value** |
| --- | --- | --- | --- | --- | --- | --- |
| **Demographics**  Age, years, mean (SD) | 56·1 (19·92) | 50·1 (18·85) | 61·4 (15·73) | 57·9 (18·30) | 0·87 | >0·99 |
| Age, years, patients that had a seizure (%)  <5 | 3 (100·0) | - | - | - | >0·99 | >0·99 |
| 5-18 | 2 (50·0) | 1 (50·0) | 1 (100·0) | 0 (0·0) | >0·99 | >0·99 |
| 18-40 | 10 (45·5) | 3 (50·0) | 2(25·0) | 16 (28·1) | 0·38 | >0·99 |
| 40-60 | 22 (50·0) | 6 (37·5) | 1 (9·1) | 31 (34·4) | 0·065 | >0·99 |
| >60 | 46 (60·5) | 8 (20·8) | 8 (25·0) | 39 (34·2) | *****<0·0001** | ****<0·0025** |
| Sex, patients that had a seizure (%)  Male | 39 (50·0) | 6 (26·1) | 7 (22·6) | 57 (34·8) | *0·015 | 0·38 |
| **Observations on arrival, patients that had a seizure (%)** | |  |  |  |  |  |
| Temperature >38°C | 12 (69·2) | 4 (33·3) | 3 (17·6) | 8 (28·6) | 0·23 | >0·99 |
| GCS <8 | 27 (93·1) | 2 (100·0) | 2 (66·7) | 17 (35·4) | ***** <0·0001** | ****<0·0025** |
| 9-12 | 11 (73·3) | 3 (60·0) | 3 (25·0) | 24 (37·5) | *0·032 | 0·80 |
| 13-14 | 13 (68·4) | 5 (23·8) | 5 (15·6) | 26 (35·6) | ** 0·0035 | 0·088 |
| 15 | 32 (37·2) | 2 (11·8) | 2 (40·0) | 19 (24·7) | 0·09 | >0·99 |
| **Aetiology,** **patients that had a seizure (%)** | |  |  |  |  |  |
| Antibody-associated | 22 (75·9) | - | - | 8 (38·1) | ***** <0·0001** | ****<0·0025** |
| HSV | 13 (100·0) | 1 (20·0) | 5 (71·4) | 7 (36·8) | ***** 0·00010** | **** 0·0025** |
| VZV | 1 (20·0) | 0 (0·0) | 1 (12·5) | 1 (25·0) | >0·99 | >0·99 |
| ADEM | 18 (56·3) | 6 (37·5) | 2 (66·7) | 4 (26·7) | 0·20 | >0·99 |
| *M.tuberculosis* | 0 (0·0) | 0 (0·0) | 1 (100·0) | - | 0·33 | >0·99 |
| Bacterial | 3 (17·6) | 1 (50·0) | 3 (18·8) | 0 (0·0) | 0·69 | >0·99 |
| Other | 8 (29·6) | 2 (28·6) | 0 (0·0) | 65 (32·8) | 0·33 | >0·99 |
| Unknown | 18 (69·2) | 5 (33·3) | 0 (0·0) | 1 (25·0) | ** 0·0022 | 0·055 |
| **Inpatient tests, patients that had a seizure (%)** | |  |  |  |  |  |
| MRI abnormal | 64 (62·1) | 10 (33·3) | 6 (19·4) | 18 (35·3) | ***** <0·0001** | ****<0·0025** |
| EEG focal abnormality | 34 (81·0) | 5 (41·7) | 4 (66·7) | 77 (84·6) | ** 0·0079 | 0·20 |
| EEG consistent with encephalitis | 40 (62·5) | 7 (28·0) | 5 (31·3) | 8 (53·3) | ** 0·0088 | 0·22 |
| Low CSF:serum glucose | 9 (30·0) | 2 (22·2) | 6 (22·2) | 30 (33·0) | 0·75 | >0·99 |
| High lactate | 4 (100·0) | 2 (100·0) | 2 (50·0) | - | 0·29 | >0·99 |

*Table S2-* A table detailing the percentage of patients that had seizures within a given variable for each European country with univariate analysis. Hyphen indicates no patients in that variable irrespective of seizure status.

Bold font highlight significant differences after Bonferroni correction for multiple analyses(* p<0·05; ** p<0·01; *** p<0·001)

*Abbreviations: acute disseminating encephalomyelitis (ADEM), cerebrospinal fluid (CSF), electroencephalogram (EEG), Glasgow Coma Scale (GCS), Herpes simplex virus (HSV),magnetic resonance imaging (MRI), Mycobacterium tuberculosis (m.tuberculosis), standard deviation (SD), Varicella zoster virus (VZV)*

| *Antibody subtype* | *Total* | *Seizure* | *No Seizure* |  | *PPV* | *NPV* | *OR (95%CI)* | *p-value* |
| --- | --- | --- | --- | --- | --- | --- | --- | --- |
| **LGI1** | **31** | **19** | **12** |  | **0.61** | **0.68** | **3.29 (1.57 to 6.67)** | **** 0.0015** |
| **Ri** | **13** | **10** | **3** |  | **0.77** | **0.67** | **6.85 (2.03 to 23.31)** | **** 0.0015** |
| Ma | 4 | 2 | 2 |  | 0.50 | 0.67 | 2.02 (0.32 to 12.93) | 0.60 |
| Hu | 6 | 4 | 2 |  | 0.67 | 0.67 | 4.06 (0.94 to 21.39) | 0.10 |
| **GABA-A receptor** | **5** | **5** | **0** |  | **1.00** | **1.00** | **-** | **** 0.0039** |
| GABA-B receptor | 3 | 2 | 1 |  | 0.67 | 0.67 | 4.05 (0.47 to 58.69) | 0.26 |
| Yo | 1 | 1 | 0 |  | 1.00 | 1.00 | - | 0.33 |
| GFAP | 6 | 4 | 2 |  | 0.67 | 0.67 | 4.06 (0.94 to 21.39) | 0.10 |
| **GAD** | **24** | **15** | **9** |  | **0.63** | **0.67** | **3.44 (1.58 to 8.19)** | **** 0.0036** |
| NMDA receptor | 20 | 6 | 14 |  | 0.30 | 0.67 | 0.86 (0.34 to 2.26) | >0.99 |
| CASPR2 | 7 | 1 | 6 |  | 0.14 | 0.67 | 0.33 (0.03 to 2.02) | 0.44 |
| TPO | 1 | 1 | 0 |  | 1.00 | 1.00 | - | 0.33 |
| IgLon5 | 2 | 0 | 2 |  | 0.00 | 0.67 | - | >0.99 |

Table S3: A summary table of the seizure risk for all autoantibody subtypes identified in the global cohort.

Bold font highlights significant differences; * p<0.05; ** p<0.01; *** p<0.001.

Fisher’s exact test was for statistical analysis .

*Abbreviations: confidence interval (CI), contactin-associated protein-like 2 (CASPR2), gamma aminobutyric acid (GABA), glial fibrillary acidic protein (GFAP), glutamic acid decarboxylase (GAD), immunoglobulin-like cell adhesion molecule 5 (IgLon5), leucine-rich glioma-inactivated 1 (LGI1), negative predictive value (NPV), N-methyl-D-aspartate (NMDA), odds ratio (OR) positive predictive value (PPV), thyroid peroxidase (TPO).*

| *Multivariate regression model results* | | |
| --- | --- | --- |
| ***Term*** | ***Odds ratio (95%CI)*** | ***p-value*** |
| (Intercept) | 1.10 (0.15 to 8.19) | 0.927 |
| *Region*  EMR | 0.56 (0.14 to 2.13) | 0.40 |
| **SEAR** | **0.03 (0.01 to 0.10)** | *****<0.001** |
| WPR | 0.34 (0.10 to 1.17) | 0.09 |
| AMR | 1.07 (0.32 to 3.58) | 0.92 |
| EUR | 0.59 (0.22 to 1.62) | 0.31 |
| AFR | 1.00 | - |
| *Sex*  Male | 0.81 (0.53 to 1.24) | 0.33 |
| Female | 1.00 | - |
| *Aetiology*  HSV | 3.50 (0.86 to 15.37) | 0.09 |
| Antibody-associated | 1.60 (0.40 to 6.90) | 0.52 |
| Bacterial | 2.68 (0.57 to 13.22) | 0.22 |
| ADEM/immune | 1.93 (0.49 to 8.30) | 0.36 |
| Mycobacterium tuberculosis | 1.52 (0.28 to 8.50) | 0.63 |
| Infection (other) | 1.73 (0.47 to 6.91) | 0.42 |
| Unknown | 1.28 (0.31 to 5.69) | 0.74 |
| VZV | 1.00 | - |
| *Age*  ≤5 | 0.72 (0.09 to 5.67) | 0.75 |
| **>5 to ≤18** | **3.98 (1.51 to 10.82)** | ****<0.01** |
| >18 to ≤40 | 0.85 (0.49 to 1.48) | 0.57 |
| >40 to ≤60 | 0.86 (0.51 to 1.45) | 0.58 |
| >60 | 1.00 | - |
| *Observations on arrival*  **GCS (per point)** | **0.84 (0.78 to 0.91)** | *****<0.001** |
| Temperature ≥38°C | 0.88 (0.49 to 1.57) | 0.66 |
| *Presenting complaint*  Fever | 0.87 (0.52 to 1.44) | 0.59 |
| **Seizure** | **49.90 (31.68 to 81.02)** | *****<0.001** |
| *Taking long term anti-seizure medication* | 1.02 (0.48 to 2.20) | 0.96 |

*Table S4:* A table detailing multivariate binary logistic regression analysis of variables found to be significant in univariate analysis and from the original UK SEIZURE score analysis by Wood *et al.* The dependent variable was inpatient seizure status.

Bold font highlight significant differences (* p<0.05; ** p<0.01; *** p<0.001).

*Abbreviations: acute disseminated encephalomyelitis (ADEM), African region (AFR), region of the Americas (AMR), confidence intervals (CI), Eastern Mediterranean region (EMR), European region (EUR),* Glasgow Coma Scale (GCS), *Herpes simplex virus (HSV), South-East Asia region (SEAR), Varicella zoster virus (VZV), Western Pacific region (WPR).*

| **Region**  (patients that had inpatient seizures/total**)**  *Global (439/1324)* | *Basic scoring system* | | | | | *Advanced scoring system* | | | | | |
| --- | --- | --- | --- | --- | --- | --- | --- | --- | --- | --- | --- |
|  | Score category | Patients that had inpatient seizures/total | PPV | OR | p-value *(adjusted p-value)* |  | Score category | Patients that had inpatient seizures/total | PPV | OR | p-value *(adjusted p-value)* |
|  | 0-2 (REF) | 82/229 | - | - | - |  | 0-4 (REF) | 18/59 | - | - | - |
|  | 3-6 (REF) | 175/615 | - | - | - |  | 5-11 (REF) | 129/514 | - | - | - |
|  | 7-8 | 71/224 | 52·6 | 1·06 (0·77 to 1·46) | 0·74 (>0·99) |  | 12-13 | 54/191 | 28·1 | 1·14 (0·79 to 1·66) | 0·51 (>0·99) |
|  | 9-11 | **62/149** | **41·6** | **1·63 (1·14 to 2·33)** | *** 0·010 (0·030)** |  | 14-16 | **90/247** | **35·5** | **1·66 (1·20 to 2·28)** | ****0·0025 (0·0075)** |
|  | 12+ | **49/107** | **45·8** | **1·93 (1·30 to 2·91)** | **** 0·0020 (0·0060)** |  | 17+ | **148/467** | **47·2** | **2·60 (1·94 to 3·47)** | *****<0·0001 (<0·0003)** |
| *Africa (24/52)* | 0-2 (REF) | 1/1 | - | - | - |  | 0-4 (REF) | 1/1 | - | - | - |
|  | 3-6 (REF) | 14/28 | - | - | - |  | 5-11 (REF) | 10/19 | - | - | - |
|  | 7-8 | 6/16 | 37·5 | 0·56 (0·17 to 1·80) | 0·53 (>0·99) |  | 12-13 | 9/19 | 47·4 | 0·74 (0·20 to 2·52) | 0·75 (>0·99) |
|  | 9-11 | 3/5 | 60·0 | 1·40 (0·25 to 8·71) | >0·99 (>0·99) |  | 14-16 | 4/12 | 33·3 | 0·40 (0·11 to 1·68) | 0·29 (0·87 |
|  | 12+ | 0/2 | 0·0 | - | 0·48 (>0·99) |  | 17+ | 0/1 | 0·0 | - | 0·48 (>0·99) |
| *Eastern Mediterranean (19/51)* | 0-2 (REF) | 4/8 | - | - | - |  | 0-4 (REF) | 0/3 | - | - | - |
|  | 3-6 (REF) | 7/26 | - | - | - |  | 5-11 (REF) | 8/23 | - | - | - |
|  | 7-8 | 5/12 | 41·7 | 1·49 (0·40 to 5·59) | 0·73 (>0·99) |  | 12-13 | 1/5 | 20·0 | 0·56 (0·04 to 4·78) | >0·99 (>0·99) |
|  | 9-11 | 2/4 | 50·0 | 2·09 (0·29 to 14·40) | 0·59 (>0·99) |  | 14-16 | 7/13 | 53·9 | 2·63 (0·60 to 9·95) | 0·19 (0·57) |
|  | 12+ | 1/1 | 100 | - | 0·34 (>0·99) |  | 17+ | 3/7 | 42·9 | 1·69 (0·35 to 7·78) | 0·66 (>0·99) |
| *Southeast Asia (21/175)* | 0-2 (REF) | 2/11 | - | - | - |  | 0-4 (REF) | 0/0 | - | - | - |
|  | 3-6 (REF) | 6/56 | - | - | - |  | 5-11 (REF) | 3/25 | - | - | - |
|  | 7-8 | 2/41 | 4·9 | 0·37 (0·08 to 1·69) | 0·31 (0·93) |  | 12-13 | 1/30 | 3·3 | 0·24 (0·02 to 1·76) | 0·31 (0·93) |
|  | 9-11 | 5/31 | 16·1 | 1·39 (0·46 to 4·19) | 0·75 (>0·99) |  | 14-16 | 3/40 | 7·5 | 0·57 (0·12 to 2·63) | 0·66 (>0·99) |
|  | 12+ | 6/36 | 16·7 | 1·45 (0·44 to 4·28) | 0·56 (>0·99) |  | 17+ | 14/80 | 17·5 | 1·49 (0·41 to 5·22) | 0·76 (>0·99) |
| *Western Pacific (25/68)* | 0-2 (REF) | 1/3 | - | - | - |  | 0-4 (REF) | 0/3 | - | - | - |
|  | 3-6 (REF) | 11/33 | - | - | - |  | 5-11 (REF) | 13/32 | - | - | - |
|  | 7-8 | 4/16 | 25·0 | 1·57 (0·45 to 5·15) | 0·75 (>0·99) |  | 12-13 | 1/5 | 20·0 | 0·42 (0·03 to 3·20) | 0·64 (>0·99) |
|  | 9-11 | 3/7 | 42·9 | 0·70 (0·17 to 3·14) | 0·69 (>0·99) |  | 14-16 | 4/10 | 28·6 | 0·68 (0·20 to 2·46) | 0·74 (>0·99) |
|  | 12+ | 6/8 | 75 | 6·00 (1·14 to 31·29) | *0·048 (0·14) |  | 17+ | 7/13 | 53·9 | 1·97 (0·52 to 6·41) | 0·34 (>0·99) |
| *Region of the Americas (152/455)* | 0-2 (REF) | 28/80 | - | - | - |  | 0-4 (REF) | 3/12 | - | - | - |
|  | 3-6 (REF) | 56/183 | - | - | - |  | 5-11 (REF) | 24/135 | - | - | - |
|  | 7-8 | 29/87 | 34·2 | 1·10 (0·66 to 1·83) | 0·79 (>0·99) |  | 12-13 | 16/60 | 26·7 | 1·62 (0·80 to 3·24) | 0·19 (0·57) |
|  | 9-11 | 18/40 | 45·0 | 1·74 (0·88 to 3·34) | 0·11 (0·33) |  | 14-16 | 30/93 | 32·2 | 2·12 (1·14 to 3·77) | 0·019 (0·057) |
|  | 12+ | 21/67 | 31·3 | 0·97 (0·55 to 1·73) | >0·99 (>0·99) |  | 17+ | **79/155** | **51·0** | **4·62 (2·70 to 7·90)** | *****<0·0001 (<0·0003)** |
| *USA group (293/573 basic; 187/572 advanced)* | 0-2 (Ref) | 22/70 | 31·4 | - | - |  | 0-4 (REF) | 0/16 | 0 | - | - |
|  | 3-6 (Ref) | 64/245 | 26·1 | - | - |  | 5-11 (REF) | 41/186 | 22 | - | - |
|  | 7-8 | 40/127 | 31·5 | 1·22 (0·78 to 1·92) | 0·38 (>0·99) |  | 12-13 | 24/90 | 26·7 | 1·44 (0·81 to 2·57) | 0·22 (0·66) |
|  | 9-11 | 20/64 | 31·3 | 1·21 (0·68 to 2·17) | 0·52 (>0·99) |  | 14-16 | 37/118 | 31·4 | 1·81 (1·08 to 3·03) | 0·030 (0·090) |
|  | 12 + | **41/67** | **61·2** | **4·20 (2·42 to 7·28)** | *****<0·0001 (<0·0003)** |  | 17 + | **85/162** | **52·5** | **4·36 (2·75 to 6·92)** | *****<0·0001 (<0·0003)** |
| \| *Europe (208/576)* \| \| --- \| | 0-2 (REF) | 40/104 | - | - | - |  | 0-4 (REF) | 12/34 | - | - | - |
|  | 3-6 (REF) | 75/262 | - | - | - |  | 5-11 (REF) | 66/158 | - | - | - |
|  | 7-8 | 31/84 | 36·9 | 1·28 (0·77 to 2·09) | 0·37 (>0·99) |  | 12-13 | 27/87 | 31·0 | 1·04 (0·61 to 1·74) | 0·89 (>0·99) |
|  | 9-11 | 37/91 | 40·7 | 1·50 (0·92 to 2·36) | 0·11 (0·33) |  | 14-16 | 43/109 | 39·5 | 1·50 (0·95 to 2·42) | 0·090 (0·27) |
|  | 12+ | **25/35** | **71·4** | **5·46 (2·56 to 11·13)** | *****<0·0001 (<0·0003)** |  | 17+ | **60/122** | **49·2** | **2·23 (1·45 to 3·46)** | *****<0·0001 (<0·0003)** |

Table S5- A summary of the basic and advanced SEIZURE score performances when separated into discrete categories for all study groups including the pre-analysed USA study group, ‘USA group’.

The two lowest categories are combined and used as a reference baseline.

Bold fonts denote significant differences (* p<0·05; ** p<0·01; *** p<0·001) after Bonferroni correction.

Abbreviations: odds ratios (OR), positive predictive value (PPV), Reference baseline score (REF), United States of America (USA).

| **Variable** | | | **All (n=52)** | | **Seizure (n=24)** | | | | **No seizure (n=28)** | | | | **PPV** | | | | **NPV** | | | | | | **OR (95% CI)** | | | **p-value** | | | | **Adjusted p-value** | | | |  |  |  |  |  |  |  |  |  |  |  |  |  |  |
| --- | --- | --- | --- | --- | --- | --- | --- | --- | --- | --- | --- | --- | --- | --- | --- | --- | --- | --- | --- | --- | --- | --- | --- | --- | --- | --- | --- | --- | --- | --- | --- | --- | --- | --- | --- | --- | --- | --- | --- | --- | --- | --- | --- | --- | --- | --- | --- |
| **Demographic**  Age, years, median (IQR) | | | 43 (28-51·5) | | 45 (34-56·25) | | | | 40·5 (27·5-47) | | |  | | | | |  | | | | | | |  | | 0·40 | | | | >0·99 | | | |  |  |  |  |  |  |  |  |  |  |  |  |  |  |
| Age, years, n (%)  ≤ 5 | | | 0 (0·0) | | 0 (0·0) | | | | 0 (0·0) | | | 0·0 | | | | | 53·8 | | | | | - | | | | 0·43 | | | | >0·99 | | | |  |  |  |  |  |  |  |  |  |  |  |  |  |  |
| >5 to ≤ 18 | | | 0 (0·0) | | 0 (0·0) | | | | 0 (0·0) | | | 0·0 | | | | | 53·8 | | | | | - | | | |  | | | |  | | | |  |  |  |  |  |  |  |  |  |  |  |  |  |  |
| >18 to ≤ 40 | | | 22 (42·3) | | 8 (33·3) | | | | 14 (50) | | | 36·4 | | | | | 46·7 | | | | | 0·50 (0·16 to 1·54) | | | |  | | | |  | | | |  |  |  |  |  |  |  |  |  |  |  |  |  |  |
| >40 to ≤ 60 | | | 19 (36·5) | | 11 (45·8) | | | | 8 (28·6) | | | 57·9 | | | | | 60·6 | | | | | 2·12 (0·67 to 6·66) | | | |  | | | |  | | | |  |  |  |  |  |  |  |  |  |  |  |  |  |  |
| >60 | | | 11 (21·2) | | 5 (20·8) | | | | 6 (21·4) | | | 45·5 | | | | | 53·7 | | | | | 0·96 (0·25 to 3·67) | | | |  | | | |  | | | |  |  |  |  |  |  |  |  |  |  |  |  |  |  |
| Sex, n (%)  Male | | | 29 (55·8) | | 13 (54·2) | | | | 16 (57·1) | | | 44·8 | | | | | 52·2 | | | | | 0·89 (0·30 to 2·66) | | | | >0·99 | | | | >0·99 | | | |  |  |  |  |  |  |  |  |  |  |  |  |  |  |
| **Aetiology, n (%)**  Autoantibody-associated | | | 0 (0·0) | | 0 (0·0) | | | | 0 (0·0) | | | - | | | | | 53·8 | | | | | - | | | | * 0·010 | | | | 0·22 | | | |  |  |  |  |  |  |  |  |  |  |  |  |  |  |
| HSV | | | 23 (44·2) | | 12 (50·0.) | | | | 11 (39·3) | | | 52·2 | | | | | 58·6 | | | | | 1·55 (0·51 to 4·65) | | | |  | | | |  | | | |  |  |  |  |  |  |  |  |  |  |  |  |  |  |
| VZV | | | 4 (7·7) | | 2 (8·3) | | | | 2 (7·1) | | | 50·0 | | | | | 54·2 | | | | | 1·82 (0·15 to 9·09) | | | |  | | | |  |  |  |  |  |  |  |  |  |  |  |  |  |  |  |  |  |  |
| Bacterial | | | 0 (0·0) | | 0 (0·0) | | | | 0 (0·0) | | | - | | | | | 53·8 | | | | | - | | | |  | | | |  |  |  |  |  |  |  |  |  |  |  |  |  |  |  |  |  |  |
| ADEM/immune | | | 0 (0·0) | | 0 (0·0) | | | | 0 (0·0) | | | - | | | | | 53·8 | | | | | - | | | |  | | | |  |  |  |  |  |  |  |  |  |  |  |  |  |  |  |  |  |  |
| Mycobacterium TB | | | 4 (7·7) | | 3 (12·5) | | | | 1 (3·6) | | | 75·0 | | | | | 56·3 | | | | | 3·86 (0·37 to 39·80) | | | |  | | | |  | | | | | | |  | | | | | | | | | |  |
| Infection (other) | | | 21 (40·4) | | 7 (29·2) | | | | 14 (50·0) | | | 33·3 | | | | | 45·2 | | | | | 0·41 (0·13 to 1·30) | | | |  | | | |  | | | | | | |  |  |  |  |  |  |  |  |  |  |  |
| Unknown | | | 0 (0·0) | | 0 (0·0) | | | | 0 (0·0) | | | - | | | | | 53·8 | | | | | - | | | |  | | | |  | | | | | | |  |  |  |  |  |  |  |  |  |  |  |
| Immunosuppressed, n (%) | | | 16 (30·8) | | 8 (33·3) | | | | 8 (28·6) | | | 50·0 | | | | | 55·6 | | | | | 1·25 (0·38 to 4·06) | | | | 0·77 | | | | >0·99 | | | | | | |  |  |  |  |  |  |  |  |  |  |  |
| HIV positive, n (%) | | | 9 (17·3) | | 3 (12·5) | | | | 6 (21·4) | | | 33·3 | | | | | 51·2 | | | | | 0·52 (0·12 to 2·37) | | | | 0·48 | | | | >0·99 | | | | | | |  |  |  |  |  |  |  |  |  |  |  |
|  | | |  | |  | | | |  | | |  | | | | |  | | | | |  | | | |  | | | |  | | | | | | |  |  |  |  |  |  |  |  |  |  |  |
| **Symptoms**  Median symptom duration prior to admission, days (IQR, range) | | | - | | - | | | | - | | |  | | | | |  | | | | |  | | | | - | | | | - | | | | | | |  |  |  |  |  |  |  |  |  |  |  |
| GCS at presentation, n/total | | |  | |  | | | |  | | |  | | | | |  | | | | |  | | | |  | | | |  | | | | | | |  |  |  |  |  |  |  |  |  |  |  |
| ≤ 8 | | | 0/52 | | 0/24 | | | | 0/28 | | | - | | | | | 53·8 | | | | | - | | | | - | | | | - | | | | | | |  | | | |  | | |  |  |  |  |
| 9-12. | | | 5/52 | | 2/24 | | | | 3/28 | | | 40·0 | | | | | 53·2 | | | | | 0·79 (0·12 to 4·29) | | | | >0·99 | | | | >0·99 | | | | | | |  |  |  |  |  |  |  |  |  |  |  |
| 13-14 | | | 12/52 | | 6/24 | | | | 6/28 | | | 50·0 | | | | | 55 | | | | | 1·19 (0·35 to 3·98) | | | | >0·99 | | | | >0·99 | | | | | | |  |  |  |  |  |  |  |  |  |  |  |
| 15 (ref) | | | 35/52 | | 16/24 | | | | 19/28 | | | 45·7 | | | | | 52·9 | | | | | - | | | | - | | | | - | | | | | | |  |  |  |  |  |  |  |  |  |  |  |
| Temperature over 38°C on admission, n/total | | | 43/52 | | 18/24 | | | | 25/28 | | | 41·9 | | | | | 33·3 | | | | | 0·36 (0·08 to 1·63) | | | | 0·27 | | | | >0·99 | | | | | | |  | | | |  | | |  |  |  |  |
| **Presenting symptoms, n (%)**  Fever | | | 43 (84·3) | | 19 (79·2) | | | | 24 (88·9) | | | 44·2 | | | | | 37·5 | | | | | 0·48 (0·10 to 2·24) | | | | 0·45 | | | | >0·99 | | | | | | |  | | | |  | | |  |  |  |  |
| Headache | | | 30 (57·7) | | 13 (54·2) | | | | 17 (60·7) | | | 43·3 | | | | | 50 | | | | | 0·76 (0·25 to 2·30) | | | | 0·78 | | | | >0·99 | | | | | | |  |  |  |  |  |  |  |  |  |  |  |
| Lethargy | | | 0 (0·0) | | 0 (0·0) | | | | 0 (0·0) | | | - | | | | | - | | | | | - | | | | - | | | | - | | | | | | |  |  |  |  |  |  |  |  |  |  |  |
| Seizure | | | **24 (46·2)** | | **24 (100)** | | | | **0 (0·0)** | | | **100**·0 | | | | | **-** | | | | | **-** | | | | ***** <0·0001** | | | | ****<0·0022** | | | | | | |  |  |  |  |  |  |  |  |  |  |  |
| Behavioural change | | | 28 (53·8) | | 14 (58·3) | | | | 14 (50·0) | | | 50·0 | | | | | 58·3 | | | | | 1·40 (0·47 to 4·20) | | | | 0·59 | | | | >0·99 | | | | | | |  |  |  |  |  |  |  |  |  |  |  |
| Focal neurology | | | 29 (55·8) | | 13 (54·2) | | | | 16 (57·1) | | | 44·8 | | | | | 52·2 | | | | | 0·89 (0·30 to 2·66) | | | | >0·99 | | | | >0·99 | | | | | | |  |  |  |  |  |  |  |  |  |  |  |
| Irritability | | | 0 (0) | | 0 (0) | | | | 0 (0) | | | - | | | | | - | | | | | - | | | | - | | | |  | | | | | | |  |  |  |  |  |  |  |  |  |  |  |
| Neck stiffness | | | 18 (34·6) | | 7 (29·2) | | | | 11 (39·3) | | | 38·9 | | | | | 50 | | | | | 0·64 (0·20 to 2·03) | | | | 0·56 | | | | >0·99 | | | | | | |  |  |  |  |  |  |  |  |  |  |  |
| Photophobia | | | 18 (34·6) | | 7 (29·2) | | | | 11 (39·3) | | | 38·9 | | | | | 50 | | | | | 0·64 (0·20 to 2·03) | | | | 0·56 | | | | >0·99 | | | | | | |  |  |  |  |  |  |  |  |  |  |  |
| Gastrointestinal | | | 5 (9·6) | | 2 (8·3) | | | | 3 (10·7) | | | 40·0 | | | | | 53·2 | | | | | 0·76 (0·12 to 4·96) | | | | >0·99 | | | | >0·99 | | | | | | |  |  |  |  |  |  |  |  |  |  |  |
| Respiratory | | | 2 (3·8) | | 2 (8·3) | | | | 0 (0) | | | 100·0 | | | | | 56 | | | | | - | | | | 0·21 | | | |  | | | | | | |  |  |  |  |  |  |  |  |  |  |  |
| **Diagnostic tests** | | |  | |  | | | |  | | |  | | | | |  | | | | |  | | | |  | | | |  | | | | | | |  |  |  |  |  |  |  |  |  |  |  |
| First MRI, n/total | | |  | |  | | | |  | | |  | | | | |  | | | | |  | | | |  | | | |  | | | | | | |  |  |  |  |  |  |  |  |  |  |  |
| Abnormal | | | 21/26 | | 9/12 | | | | 12/14 | | | 42·9 | | | | | 40 | | | | | 0·50 (0·07 to 3·65) | | | | 0·63 | | | | >0·99 | | | | | | |  |  |  |  |  |  |  |  |  |  |  |
| First EEG, n/total | | |  | |  | | | |  | | |  | | | | |  | | | | |  | | | |  | | | |  | | | | | | |  |  |  |  |  |  |  |  |  |  |  |
| Focal abnormality | | | - | | - | | | | - | | | - | | | | | - | | | | | - | | | | - | | | | - | | | | | | |  |  |  |  |  |  |  |  |  |  |  |
| Consistent with encephalitis | | | - | | - | | | | - | | | - | | | | | - | | | | | - | | | | - | | | | - | | | | | | |  |  |  |  |  |  |  |  |  |  |  |
| Low CSF:serum glucose ratio, n/total | | | 15/51 | | 4/23 | | | | 11/28 | | | 26·7 | | | | | 47·2 | | | | | 0·33 (0·09 to 1·22) | | | | 0·13 | | | | >0·99 | | | | | | |  |  |  |  |  |  |  |  |  |  |  |
| CSF:serum glucose ratio, mean (SD) | | | 0·611 (0·27) | | 0·632 | | | | 0·594 | | |  | | | | |  | | | | |  | | | | 0·66 | | | | >0·99 | | | | | | |  |  |  |  |  |  |  |  |  |  |  |
| Low initial serum sodium, n/total | | | 0/52 | | 0/24 | | | | 0/28 | | |  | | | | |  | | | | |  | | | | - | | | | - | | | | | | |  |  |  |  |  |  |  |  |  |  |  |
| Initial serum sodium, mmol/L, mean (SD) | | | 134·1 (4·98) | | 134·1 (5·93) | | | | 134·1 (4·12) | | | - | | | | | - | | | | | - | | | | 0·97 | | | | >0·99 | | | | | | |  |  |  |  |  |  |  |  |  |  |  |
| High initial serum lactate, n/total | | | - | | - | | | | - | | |  | | | | |  | | | | |  | | | | - | | | | - | | | | | | |  |  |  |  |  |  |  |  |  |  |  |
| Initial serum lactate, mmol/L, mean (SD) | | | - | | - | | | | - | | | - | | | | | - | | | | | - | | | | - | | | | - | | | | | | |  |  |  |  |  |  |  |  |  |  |  |
| Taking anti-seizure medication prior to admission, n/total | | | 0/52 | | 0/24 | | | | 0/28 | | | - | | | | | - | | | | | - | | | | - | | | | - | | | | | | |  |  |  |  |  |  |  |  |  |  |  |
| *Table S6:* A summary of the African region study group separated into patients that did and did not have a seizure with univariate analysis. Analysis completed using student’s t-test or fisher’s exact test.  Bold font highlight significant differences (* p<0·05; ** p<0·01; *** p<0·001) after Bonferroni correction for multiple analyses, shown as adjusted p-value.  *Abbreviations: acute disseminating encephalomyelitis (ADEM), cerebrospinal fluid (CSF), electroencephalogram (EEG), Glasgow Coma Scale (GCS), Herpes simplex virus (HSV), interquartile range (IQR), negative predictive value (NPV), magnetic resonance imaging (MRI), odds ratios (OR), positive predictive value (PPV), standard deviation (SD), tuberculosis (TB), Varicella zoster virus (VZV).* | | |  | |  | | | |  | | |  | | | | |  | | | | |  | | | |  | | | |  | | | | | | |  |  |  |  |  |  |  |  |  |  |  |
| **Variable** | | **All (n=51)** | | | | | **Seizure (n=19)** | | | **No seizure (n=32)** | | | | | | **PPV** | | | | **NPV** | | | | **OR (95% CI)** | | | | **p-value** | | | | **Adjusted p-value** | | | | |  |  |  |  |  |  |  |  |  |  |  |
| Age, years, median (IQR) | | 55 (42·5-64·5) | | | | | 63 (51-65·5) | | | 51 (40·75-62·25) | | | | | |  | | | |  | | | |  | | | | 0·11 | | | | >0·99 | | | | |  |  |  |  |  |  |  |  |  |  |  |
| Age, years, n (%)  ≤ 5 | | 0 (0·0) | | | | | 0 (0·0) | | | 0 (0·0) | | | | | | 0·0 | | | | 62·7 | | | | - | | | | 0·40 | | | | >0·99 | | | | |  |  |  |  |  |  |  |  |  |  |  |
| >5 to ≤ 18 | | 1 (2·0) | | | | | 0 (0·0) | | | 1 (3·1) | | | | | | 0·0 | | | | 62·0 | | | | - | | | |  | | | |  | | | | |  |  |  |  |  |  |  |  |  |  |  |
| >18 to ≤ 40 | | 9 (17·6) | | | | | 2 (10·5) | | | 7 (21·9) | | | | | | 22·2 | | | | 59·5 | | | | 0·42(0·08 to 2·27) | | | |  | | | |  | | | | |  |  |  |  |  |  |  |  |  |  |  |
| >40 to ≤ 60 | | 21 (41·2) | | | | | 7 (36·8) | | | 14 (43·8) | | | | | | 33·3 | | | | 60·0 | | | | 0·75 (0·23 to 2·40) | | | |  | | | |  | | | | |  |  |  |  |  |  |  |  |  |  |  |
| >60 | | 20 (39·2) | | | | | 10 (52·6) | | | 10 (31·3) | | | | | | 50·0 | | | | 71·0 | | | | 2·44 (0·76 to 7·88) | | | |  | | | |  | | | | |  |  |  |  |  |  |  |  |  |  |  |
| Sex, n (%)  Male | | 18 (35·3) | | | | | 7 (36·8) | | | 11 (34·4) | | | | | | 38·9 | | | | 50·0 | | | | 0·64 (0·18 to 2·20) | | | | >0·99 | | | | >0·99 | | | | |  |  |  |  |  |  |  |  |  |  |  |
| **Aetiology, n (%)**  Autoantibody-associated | | 4 (7·8) | | | | | 2 (10·5) | | | 2 (6·3) | | | | | | 50·0 | | | | 63·8 | | | | 1·76 (0·23 to 13·7) | | | | 0·17 | | | | >0·99 | | | | |  |  |  |  |  |  |  |  |  |  |  |
| HSV | | 13 (25·5) | | | | | 8 (42·1) | | | 5 (15·6) | | | | | | 61·5 | | | | 71·1 | | | | 3·93 (1·05 to 14·69) | | | |  | | | |  | | | | |  |  |  |  |  |  |  |  |  |  |  |
| VZV | | 3 (5·9) | | | | | 0 (0·0) | | | 3 (9·4) | | | | | | 0·0 | | | | 60·4 | | | | - | | | |  | | | |  |  |  |  |  |  |  |  |  |  |  |  |  |  |  |  |
| Bacterial | | 2 (3·9) | | | | | 1 (5·3) | | | 1 (3·1) | | | | | | 50·0 | | | | 63·3 | | | | 1·72 (0·10 to 29·24) | | | |  | | | |  |  |  |  |  |  |  |  |  |  |  |  |  |  |  |  |
| ADEM/immune | | 11 (21·6) | | | | | 3 (15·8) | | | 8(25·0) | | | | | | 27·3 | | | | 60·0 | | | | 0·56 (0·13 to 2·45) | | | |  | | | |  |  |  |  |  |  |  |  |  |  |  |  |  |  |  |  |
| Mycobacterium TB | | 0 (0·0) | | | | | 0 (0·0) | | | 0 (0·0) | | | | | | - | | | | - | | | | - | | | |  | | | |  | | | | | |  | | | | | | | | | |
| Infection (other) | | 5 (9·8) | | | | | 0 (0·0) | | | 5 (15·6) | | | | | | 0 | | | | 58·7 | | | | - | | | |  | | | |  | | | | | |  |  |  |  |  |  |  |  |  |  |
| Unknown | | 13 (25·5) | | | | | 5 (26·3) | | | 8 (25·0) | | | | | | 38·5 | | | | 63·2 | | | | 1·07 (0·30 to 3·92) | | | |  | | | |  | | | | | |  |  |  |  |  |  |  |  |  |  |
| Immunosuppressed, n (%) | | 12 (23·5) | | | | | 5 (26·3) | | | 7 (21·9) | | | | | | 41·7 | | | | 64·1 | | | | 1·28 (0·34 to 4·78) | | | | 0·74 | | | | >0·99 | | | | | |  |  |  |  |  |  |  |  |  |  |
| HIV positive, n (%) | | 0 (0·0) | | | | | 0 (0·0) | | | 0 (0·0) | | | | | | - | | | | - | | | | - | | | | - | | | |  | | | | | |  |  |  |  |  |  |  |  |  |  |
|  | |  | | | | |  | | |  | | | | | |  | | | |  | | | |  | | | |  | | | |  | | | | | |  |  |  |  |  |  |  |  |  |  |
| **Symptoms** | |  | | | | |  | | |  | | | | | |  | | | |  | | | |  | | | |  | | | |  | | | | | |  |  |  |  |  |  |  |  |  |  |
| Median symptom duration prior to admission, days (IQR, range) | | 4·5 (3-10·75, 1-60) | | | | | 4 (2·5-9, 1-30) | | | 6 (1-12, 1-60) | | | | | |  | | | |  | | | |  | | | | 0·32 | | | | >0·99 | | | | | |  |  |  |  |  |  |  |  |  |  |
| GCS at presentation, n/total | |  | | | | |  | | |  | | | | | |  | | | |  | | | |  | | | |  | | | |  | | | | | |  | | | |  | | |  |  |  |
| ≤ 8 | | 3/51 | | | | | 2/19 | | | 1/32 | | | | | | 66·7 | | | | 64·6 | | | | 6·80 (0·62 to 102·9) | | | | 0·18 | | | | >0·99 | | | | | |  |  |  |  |  |  |  |  |  |  |
| 9-12. | | 6/51 | | | | | 2/19 | | | 4/32 | | | | | | 33·3 | | | | 62·2 | | | | 1·70 (0·26 to 11·58) | | | | 0·62 | | | | >0·99 | | | | | |  |  |  |  |  |  |  |  |  |  |
| 13-14 | | 20/51 | | | | | 10/19 | | | 10/32 | | | | | | 50·0 | | | | 71·0 | | | | 3·40 (0·94 to 12·57) | | | | 0·11 | | | | >0·99 | | | | | |  |  |  |  |  |  |  |  |  |  |
| 15 (ref) | | 22/51 | | | | | 5/19 | | | 17/32 | | | | | | 22·7 | | | | 51·7 | | | |  | | | |  | | | |  | | | | | |  | | | |  | | |  |  |  |
| Temperature over 38°C on admission, n/total | | 16/51 | | | | | 8/19 | | | 8/32 | | | | | | 50·0 | | | | 68·6 | | | | 2·18 (0·65 to 7·33) | | | | 0·23 | | | | >0·99 | | | | | |  | | | |  | | |  |  |  |
| **Presenting symptoms, n (%)**  Fever | | 25 (49·0) | | | | | 10 (52·6) | | | 15 (46·9) | | | | | | 40·0 | | | | 65·4 | | | | 1·26 (0·40 to 3·93) | | | | 0·78 | | | | >0·99 | | | | | |  |  |  |  |  |  |  |  |  |  |
| Headache | | 24 (47·1) | | | | | 9 (47·4) | | | 15 (46·9) | | | | | | 37·5 | | | | 63·0 | | | | 1·02 (0·33 to 3·18) | | | | >0·99 | | | | >0·99 | | | | | |  |  |  |  |  |  |  |  |  |  |
| Lethargy | | 22 (44·0) | | | | | 11 (57·9) | | | 11 (35·4) | | | | | | 50·0 | | | | 71·4 | | | | 2·50 (0·78 to 8·06) | | | | 0·15 | | | | >0·99 | | | | | |  |  |  |  |  |  |  |  |  |  |
| Seizure | | **20 (40·8)** | | | | | **16 (88·9)** | | | **4 (12·9)** | | | | | | **80·0** | | | | **93·1** | | | | **54·00 (8·87 to 328·82)** | | | | *****<0·0001** | | | | ****<0·0028** | | | | | |  |  |  |  |  |  |  |  |  |  |
| Behavioural change | | 31 (64·6) | | | | | 14 (82·4) | | | 17 (54·8) | | | | | | 45·2 | | | | 82·4 | | | | 3·84 (0·92 to 16·12) | | | | 0·068 | | | | >0·99 | | | | | |  |  |  |  |  |  |  |  |  |  |
| Focal neurology | | 12 (23·5) | | | | | 3 (15·8) | | | 9 (28·1) | | | | | | 25·0 | | | | 59·0 | | | | 0·48 (0·11 to 2·05) | | | | 0·50 | | | | >0·99 | | | | | |  |  |  |  |  |  |  |  |  |  |
| Irritability | | 5 (9·8) | | | | | 3 (15·8) | | | 2 (6·3) | | | | | | 60·0 | | | | 65·2 | | | | 2·81 (0·43 to 18·60) | | | | 0·35 | | | | >0·99 | | | | | |  |  |  |  |  |  |  |  |  |  |
| Neck stiffness | | 6 (11·8) | | | | | 2 (10·5) | | | 4 (12·5) | | | | | | 33·3 | | | | 62·2 | | | | 0·82 (0·14 to 4·99) | | | | >0·99 | | | | >0·99 | | | | | |  |  |  |  |  |  |  |  |  |  |
| Photophobia | | 3 (5·9) | | | | | 1 (5·3) | | | 2 (6·3) | | | | | | 33·3 | | | | 62·5 | | | | 0·83 (0·07 to 9·86) | | | | >0·99 | | | | >0·99 | | | | | |  |  |  |  |  |  |  |  |  |  |
| Gastrointestinal | | 6 (11·8) | | | | | 4 (21·1) | | | 2 (6·3) | | | | | | 66·7 | | | | 66·7 | | | | 4·00 (0·66 to 24·37) | | | | 0·18 | | | | >0·99 | | | | | |  |  |  |  |  |  |  |  |  |  |
| Respiratory | | 3 (6·0) | | | | | 0 (0·0) | | | 3 (9·4) | | | | | | 0·0 | | | | 61·7 | | | | - | | | | 0·54 | | | | >0·99 | | | | | |  |  |  |  |  |  |  |  |  |  |
| **Diagnostic tests** | |  | | | | |  | | |  | | | | | |  | | | |  | | | |  | | | |  | | | |  | | | | | |  |  |  |  |  |  |  |  |  |  |
| First MRI, n/total | |  | | | | |  | | |  | | | | | |  | | | |  | | | |  | | | |  | | | |  | | | | | |  |  |  |  |  |  |  |  |  |  |
| Abnormal | | 36/48 | | | | | 15/19 | | | 21/29 | | | | | | 41·7 | | | | 66·7 | | | | 1·43 (0·36 to 5·63) | | | | 0·74 | | | | >0·99 | | | | | |  |  |  |  |  |  |  |  |  |  |
| First EEG, n/total | |  | | | | |  | | |  | | | | | |  | | | |  | | | |  | | | |  | | | |  | | | | | |  |  |  |  |  |  |  |  |  |  |
| Focal abnormality | | 2/27 | | | | | 2/16 | | | 0/11 | | | | | | 100·0 | | | | 44.-0 | | | | - | | | | 0·50 | | | | >0·99 | | | | | |  |  |  |  |  |  |  |  |  |  |
| Consistent with encephalitis | | 21/27 | | | | | 14/16 | | | 7/11 | | | | | | 66·7 | | | | 66·7 | | | | 4·00 (0·58 to 27·41) | | | | 0·19 | | | | >0·99 | | | | | |  |  |  |  |  |  |  |  |  |  |
| Low CSF:serum glucose ratio, n/total | | 22/39 | | | | | 7/16 | | | 15/23 | | | | | | 31·8 | | | | 47·1 | | | | 0·41 (0·11 to 1·53) | | | | 0·21 | | | | >0·99 | | | | | |  |  |  |  |  |  |  |  |  |  |
| CSF:serum glucose ratio, mean (SD) | | 0·50 (0·19) | | | | | 0·56 (0·27) | | | 0·46 (0·26) | | | | | | **-** | | | | **-** | | | | **-** | | | | 0·063 | | | | >0·99 | | | | | |  |  |  |  |  |  |  |  |  |  |
| Low initial serum sodium, n/total | | 0/46 | | | | | 0/16 | | | 0/30 | | | | | | - | | | | - | | | | - | | | | - | | | |  | | | | | |  |  |  |  |  |  |  |  |  |  |
| Initial serum sodium, mmol/L, mean (SD) | | 136·2 (4·67) | | | | | 135·6 (3·08) | | | 136·5 (5·35) | | | | | |  | | | |  | | | |  | | | | 0·59 | | | | >0·99 | | | | | |  |  |  |  |  |  |  |  |  |  |
| High initial serum lactate, n/total | | - | | | | | - | | | - | | | | | | - | | | | - | | | | - | | | | - | | | |  | | | | | |  |  |  |  |  |  |  |  |  |  |
| Initial serum lactate, mmol/L, mean (SD) | | - | | | | | - | | | - | | | | | |  | | | |  | | | |  | | | | - | | | |  | | | | | |  |  |  |  |  |  |  |  |  |  |
| Taking anti-seizure medication prior to admission , n/total | | 1/51 | | | | | 0/19 | | | 1/32 | | | | | | 0·0 | | | | 62·0 | | | | - | | | | >0·99 | | | | >0·99 | | | | | |  |  |  |  |  |  |  |  |  |  |
|  | |  | | | | |  | | |  | | | | | |  | | | |  | | | |  | | | |  | | | |  | | | | | |  |  |  |  |  |  |  |  |  |  |
| *Table S7:* A summary of the Eastern Mediterranean region study group separated into patients that did and did not have a seizure with univariate analysis.  Analysis completed using student’s t-test or fisher’s exact test.  Bold font highlight significant differences (* p<0·05; ** p<0·01; *** p<0·001) after Bonferroni correction for multiple analyses, shown as adjusted p-value.  *Abbreviations: acute disseminating encephalomyelitis (ADEM), cerebrospinal fluid (CSF), electroencephalogram (EEG), Glasgow Coma Scale (GCS), Herpes simplex virus (HSV), interquartile range (IQR), magnetic resonance imaging (MRI), negative predictive value (NPV), odds ratio (OR), positive predictive value (PPV), standard deviation (SD), tuberculosis (TB), Varicella zoster virus (VZV).* | |  | | | | |  | | |  | | | | | |  | | | |  | | | |  | | | |  | | | |  | | | | | |  |  |  |  |  |  |  |  |  |  |
| **Variable** | **All (n=175)** | | | | | **Seizure (n=21)** | | | | **No seizure (n=154)** | | | | | **PPV** | | | **NPV** | | | **OR (95% CI)** | | | | | | **p-value** | | | | **Adjusted p-value** | | | | |  |  |  |  |  |  |  |  |  |  |  |  |
| **Demographic**  Age, years, median (IQR) | **35 (20·5-50)** | | | | | **14 (13-24)** | | | | **37·5 (23·25-50)** | | | | |  | | |  | | |  | | | | | | *****0·00014** | | | | ****0·0045** | | | | |  |  |  |  |  |  |  |  |  |  |  |  |
| Age, years, n (%)  ≤ 5 | **6 (3·4)** | | | | | **0 (0)** | | | | **6 (3·9)** | | | | | **0·0** | | | **87·6** | | | **-** | | | | | | *****0·00030** | | | | ****0·0096** | | | | |  |  |  |  |  |  |  |  |  |  |  |  |
| >5 to ≤ 18 | **34 (19·4)** | | | | | **12 (57·1)** | | | | **22 (14·3)** | | | | | **35·3** | | | **93·6** | | | **8·00 (3·02 to 21·21)** | | | | | |  | | | |  | | | | |  |  |  |  |  |  |  |  |  |  |  |  |
| >18 to ≤ 40 | **70 (40·0)** | | | | | **7 (33·3)** | | | | **63 (40·9)** | | | | | **10·0** | | | **86·7** | | | **0·72 (0·28 to 1·89)** | | | | | |  | | | |  | | | | |  |  |  |  |  |  |  |  |  |  |  |  |
| >40 to ≤ 60 | **51 (29·1)** | | | | | **1 (4·8)** | | | | **50 (32·5)** | | | | | **2·0** | | | **83·9** | | | **0·10 (0·01 to 0·80)** | | | | | |  | | | |  | | | | |  |  |  |  |  |  |  |  |  |  |  |  |
| >60 | **15 (8·6)** | | | | | **1 (4·8)** | | | | **14 (9·1)** | | | | | **6·7** | | | **87·5** | | | **0·50 (0·06 to 4·01)** | | | | | |  | | | |  | | | | |  |  |  |  |  |  |  |  |  |  |  |  |
| Sex, n (%)  Male | 82 (46·9) | | | | | 7 (33·3) | | | | 75 (48·7) | | | | | 8·5 | | | 84·9 | | | 0·53 (0·20 to 1·38) | | | | | | 0·25 | | | | >0·99 | | | | |  |  |  |  |  |  |  |  |  |  |  |  |
| **Aetiology, n (%)**  Autoantibody-associated | 41 (23·4) | | | | | 12 (57·1) | | | | 29 (18·8) | | | | | 29·3 | | | 93·3 | | | 5·75 (2·21 to 14·92) | | | | | | **0·0035 | | | | 0·11 | | | | |  |  |  |  |  |  |  |  |  |  |  |  |
| HSV | 26 (14·9) | | | | | 1 (4·8) | | | | 25 (16·2) | | | | | 3·8 | | | 86·6 | | | 0·26 (0·03 to 2·01) | | | | | |  | | | |  | | | | |  |  |  |  |  |  |  |  |  |  |  |  |
| VZV | 0 (0) | | | | | 0 (0) | | | | 0 (0) | | | | | - | | | 88·0 | | | - | | | | | |  | | | |  |  |  |  |  |  |  |  |  |  |  |  |  |  |  |  |  |
| Bacterial | 38 (21·7) | | | | | 3 (14·3) | | | | 35 (22·7) | | | | | 7·9 | | | 86·9 | | | 0·57 (0·16 to 2·04) | | | | | |  | | | |  |  |  |  |  |  |  |  |  |  |  |  |  |  |  |  |  |
| ADEM/immune | 14 (8·0) | | | | | 3 (14·3) | | | | 11 (7·1) | | | | | 21·4 | | | 88·8 | | | 2·17 (0·55 to 8·51) | | | | | |  | | | |  |  |  |  |  |  |  |  |  |  |  |  |  |  |  |  |  |
| Mycobacterium TB | 27 (15·4) | | | | | 0 (0) | | | | 27 (17·5) | | | | | 0·0 | | | 85·8 | | | - | | | | | |  | | | |  | | | | | | | |  | | | | | | | |  |
| Infection (other) | 21 (12·0) | | | | | 2 (9·5) | | | | 19 (12·3) | | | | | 9·5 | | | 87·7 | | | 0·75 (0·16 to 2·04) | | | | | |  | | | |  | | | | | | | |  |  |  |  |  |  |  |  |  |
| Unknown | 8 (4·6) | | | | | 0 (0) | | | | 8 (5·2) | | | | | 0·0 | | | 87·4 | | | - | | | | | |  | | | |  | | | | | | | |  |  |  |  |  |  |  |  |  |
| Immunosuppressed, n (%) | 18 (10·3) | | | | | 1 (4·8) | | | | 17 (11·0) | | | | | 5·6 | | | 87·3 | | | 0·40 (0·05 to 3·20) | | | | | | 0·70 | | | | >0·99 | | | | | | | |  |  |  |  |  |  |  |  |  |
| HIV positive, n (%) | 18 (10·3) | | | | | 1 (4·8) | | | | 17 (11·0) | | | | | 5·6 | | | 87·3 | | | 0·40 (0·05 to 3·20) | | | | | | 0·70 | | | | >0·99 | | | | | | | |  |  |  |  |  |  |  |  |  |
|  |  | | | | |  | | | |  | | | | |  | | |  | | |  | | | | | |  | | | |  | | | | | | | |  |  |  |  |  |  |  |  |  |
| **Symptoms**  Median symptom duration, days (IQR, range) | 12 (5-30, 1-365) | | | | | 16 (7-40, 2-330) | | | | 10 (1-30, 1-365) | | | | |  | | |  | | |  | | | | | | 0·66 | | | | >0·99 | | | | | | | |  |  |  |  |  |  |  |  |  |
| GCS at presentation, n/total |  | | | | |  | | | |  | | | | |  | | |  | | |  | | | | | |  | | | |  | | | | | | | |  | |  | | |  |  |  |  |
| ≤ 8 | 27/175 | | | | | 7/21 | | | | 20/154 | | | | | 25·9 | | | 90·5 | | | 5·85 (1·23-29·67) | | | | | | *0·031 | | | | 0·96 | | | | | | | |  |  |  |  |  |  |  |  |  |
| 9-12. | 67/175 | | | | | 8/21 | | | | 59/154 | | | | | 11·9 | | | 88·0 | | | 2·31 (0·50 to 11·21) | | | | | | 0·49 | | | | >0·99 | | | | | | | |  |  |  |  |  |  |  |  |  |
| 13-14 | 45/175 | | | | | 4/21 | | | | 41/154 | | | | | 8·9 | | | 86·9 | | | 1·66 (0·37 to 9·08) | | | | | | 0·69 | | | | >0·99 | | | | | | | |  |  |  |  |  |  |  |  |  |
| 15 (ref) | 36/175 | | | | | 2/21 | | | | 34/154 | | | | | 5·6 | | | 86·3 | | | - | | | | | | - | | | | - | | | | | | | |  | |  | | |  |  |  |  |
| Temperature over 38°C on admission, n/total | 23/113 | | | | | 0/11 | | | | 23/102 | | | | | 0·0 | | | 87·8 | | | - | | | | | | 0·11 | | | | >0·99 | | | | | | | |  | |  | | |  |  |  |  |
| **Presenting symptoms, n (%)**  Fever | 96/174 | | | | | 10/21 | | | | 86/153 | | | | | 10·4 | | | 85·9 | | | 0·71 (0·28 to 1·77) | | | | | | 0·49 | | | | >0·99 | | | | | | | |  |  |  |  |  |  |  |  |  |
| Headache | 109/175 | | | | | 7/21 | | | | 102/153 | | | | | 6·4 | | | 78·8 | | | 0·25 (0·10 to 0·67) | | | | | | **0·0069 | | | | 0·22 | | | | | | | |  |  |  |  |  |  |  |  |  |
| Lethargy | 96/175 | | | | | 16/21 | | | | 80/153 | | | | | 16·7 | | | 93·7 | | | 2·96 (1·03 to 8·48) | | | | | | 0·059 | | | | >0·99 | | | | | | | |  |  |  |  |  |  |  |  |  |
| Seizure | **56/175** | | | | | **16/21** | | | | **40/153** | | | | | **28·6** | | | **95·8** | | | **9·12 (3·14 to 26·50)** | | | | | | *****<0·0001** | | | | ****<0·01** | | | | | | | |  |  |  |  |  |  |  |  |  |
| Behavioural change | 145/175 | | | | | 20/21 | | | | 125/153 | | | | | 13·8 | | | 96·7 | | | 4·64 (0·60 to 35·99) | | | | | | 0·13 | | | | >0·99 | | | | | | | |  |  |  |  |  |  |  |  |  |
| Focal neurology | 46/175 | | | | | 5/21 | | | | 41/153 | | | | | 10·9 | | | 87·6 | | | 0·86 (0·30 to 2·50) | | | | | | >0·99 | | | | >0·99 | | | | | | | |  |  |  |  |  |  |  |  |  |
| Irritability | 56/175 | | | | | 3/21 | | | | 53/153 | | | | | 5·4 | | | 84·9 | | | 0·32 (0·09 to 1·13) | | | | | | 0·081 | | | | >0·99 | | | | | | | |  |  |  |  |  |  |  |  |  |
| Neck stiffness | 80/175 | | | | | 6/21 | | | | 74/153 | | | | | 7·5 | | | 84·2 | | | 0·43 (0·16 to 1·17) | | | | | | 0·11 | | | | >0·99 | | | | | | | |  |  |  |  |  |  |  |  |  |
| Photophobia | 47/175 | | | | | 3/21 | | | | 44/153 | | | | | 6·4 | | | 85·9 | | | 0·42 (0·12 to 1·49) | | | | | | 0·20 | | | | >0·99 | | | | | | | |  |  |  |  |  |  |  |  |  |
| Gastrointestinal | 16/175 | | | | | 1/21 | | | | 15/153 | | | | | 6·3 | | | 87·4 | | | 0·46 (0·06 to 3·70) | | | | | | 0·70 | | | | >0·99 | | | | | | | |  |  |  |  |  |  |  |  |  |
| Respiratory | 28/175 | | | | | 0/21 | | | | 28/153 | | | | | 0·0 | | | 85·7 | | | - | | | | | | *0·028 | | | | 0·96 | | | | | | | |  |  |  |  |  |  |  |  |  |
| **Diagnostic tests** |  | | | | |  | | | |  | | | | |  | | |  | | |  | | | | | |  | | | |  | | | | | | | |  |  |  |  |  |  |  |  |  |
| First MRI, n/total |  | | | | |  | | | |  | | | | |  | | |  | | |  | | | | | |  | | | |  | | | | | | | |  |  |  |  |  |  |  |  |  |
| Abnormal | 133/169 | | | | | 15/21 | | | | 118/148 | | | | | 11·3 | | | 83·3 | | | 0·64 (0·23 to 1·78) | | | | | | 0·40 | | | | >0·99 | | | | | | | |  |  |  |  |  |  |  |  |  |
| First EEG, n/total |  | | | | |  | | | |  | | | | |  | | |  | | |  | | | | | |  | | | |  | | | | | | | |  |  |  |  |  |  |  |  |  |
| Focal abnormality | 8/119 | | | | | 2/16 | | | | 6/103 | | | | | 25·0 | | | 87·4 | | | 2·56 (0·37 to 14·50) | | | | | | 0·57 | | | | >0·99 | | | | | | | |  |  |  |  |  |  |  |  |  |
| Consistent with encephalitis | 85/119 | | | | | 11/16 | | | | 74/103 | | | | | 12·9 | | | 85·3 | | | 1·14 (0·29 to 4·08) | | | | | | >0·99 | | | | >0·99 | | | | | | | |  |  |  |  |  |  |  |  |  |
| Low CSF:serum glucose ratio, n/total | 66/140 | | | | | 2/11 | | | | 64/129 | | | | | 3·0 | | | 87·8 | | | 0·23 (0·05 to 1·09) | | | | | | 0·060 | | | | >0·99 | | | | | | | |  |  |  |  |  |  |  |  |  |
| CSF:serum glucose ratio, mean | 0·53 (0·23) | | | | | 0·59 (0·22) | | | | 0·52 (0·24) | | | | | **-** | | | **-** | | | **-** | | | | | | 0·34 | | | | >0·99 | | | | | | | |  |  |  |  |  |  |  |  |  |
| Low initial serum sodium, n/total | 5/175 | | | | | 1/21 | | | | 4/154 | | | | | 20·0 | | | 88·2 | | | 1·88 (0·20 to 17·62) | | | | | | 0·47 | | | | >0·99 | | | | | | | |  |  |  |  |  |  |  |  |  |
| Initial serum sodium, mmol/L, mean (SD) | 135·5 (6·25) | | | | | 137·8 (5·53) | | | | 135·2 (6·30) | | | | | - | | | - | | | - | | | | | | 0·083 | | | | >0·99 | | | | | | | |  |  |  |  |  |  |  |  |  |
| High initial serum lactate, n/total | 25/79 | | | | | 1/9 | | | | 24/70 | | | | | 4·0 | | | 85·2 | | | 0·24 (0·03 to 2·03) | | | | | | 0·26 | | | | >0·99 | | | | | | | |  |  |  |  |  |  |  |  |  |
| Initial serum lactate, mmol/L, mean (SD) | 3·62 (2·13) | | | | | 2·51 (1·20) | | | | 3·76 (2·40) | | | | | **-** | | | **-** | | | **-** | | | | | | 0·10 | | | | >0·99 | | | | | | | |  |  |  |  |  |  |  |  |  |
| Taking anti-seizure meds on admission, n/total | 5/175 | | | | | 1/21 | | | | 4/154 | | | | | 20·0 | | | 88·2 | | | 1·88 (0·20 to 17·62) | | | | | | 0·48 | | | | >0.99 | | | | | | | |  |  |  |  |  |  |  |  |  |
| *Table S8:* A summary of the South-East Asian region study group separated in patients that did and did not have a seizure with univariate analysis.  Analysis completed using student’s t-test or fisher’s exact test.  Bold font highlight significant differences (* p<0·05; ** p<0·01; *** p<0·001) after Bonferroni correction for multiple analyses, shown as adjusted p-value.  *Abbreviations: acute disseminating encephalomyelitis (ADEM), cerebrospinal fluid (CSF), electroencephalogram (EEG), Glasgow Coma Scale (GCS), Herpes simplex virus (HSV), interquartile range (IQR) magnetic resonance imaging (MRI), negative predictive value (NPV), odds ratio (OR), positive predictive value (PPV),standard deviation (SD), tuberculosis (TB), Varicella zoster virus (VZV).* |  | | | | |  | | | |  | | | | |  | | |  | | |  | | | | | |  | | | |  | | | | | | | |  |  |  |  |  |  |  |  |  |
| **Variable** | | **All (n=68)** | | **Seizure (n=25)** | | | | **No seizure (n=43)** | | | **PPV** | | | **NPV** | | | | | **OR (95% CI)** | | | | | | **p-value** | | | | **Adjusted p-value** | | | | | |  |  |  |  |  |  |  |  |  |  |  |  |  |
| Age, years, median (IQR) | | 41 (28-55·5) | | 34 (24-50) | | | | 45 (32-62·5) | | |  | | |  | | | | |  | | | | | | 0·12 | | | | >0·99 | | | | | |  |  |  |  |  |  |  |  |  |  |  |  |  |
| Age, years, n (%)  ≤ 5 | | 0 (0) | | 0 (0) | | | | 0 (0) | | | - | | | 63·2 | | | | | **-** | | | | | | 0·067 | | | | >0·99 | | | | | |  |  |  |  |  |  |  |  |  |  |  |  |  |
| >5 to ≤ 18 | | 3 (4·4) | | 3 (12) | | | | 0 (0) | | | 100 | | | 66·2 | | | | | - | | | | | |  | | | |  | | | | | |  |  |  |  |  |  |  |  |  |  |  |  |  |
| >18 to ≤ 40 | | 30 (44·1) | | 13 (52) | | | | 17 (39·5) | | | 43·3 | | | 68·4 | | | | | 1·66 (0·61 to 4·48) | | | | | |  | | | |  | | | | | |  |  |  |  |  |  |  |  |  |  |  |  |  |
| >40 to ≤ 60 | | 19 (27·9) | | 5 (20) | | | | 14 (32·6) | | | 26·3 | | | 59·2 | | | | | 0·52 (0·16 to 1·67) | | | | | |  | | | |  | | | | | |  |  |  |  |  |  |  |  |  |  |  |  |  |
| >60 | | 16 (23·5) | | 4 (16) | | | | 12 (27·9) | | | 25 | | | 59·6 | | | | | 0·49 (0·14 to 1·73) | | | | | |  | | | |  | | | | | |  |  |  |  |  |  |  |  |  |  |  |  |  |
| Sex, n (%)  Male | | 42 (61·8) | | 13 (52) | | | | 29 (67·4) | | | 31 | | | 53·8 | | | | | 0·52 (0·19 to 1·44) | | | | | | 0·30 | | | | >0·99 | | | | | |  |  |  |  |  |  |  |  |  |  |  |  |  |
| **Aetiology, n (%)**  Autoantibody-associated | | 1 (1·5) | | 1 (4) | | | | 0 (0) | | | 100 | | | 64·2 | | | | | - | | | | | | 0·43 | | | | >0·99 | | | | | |  |  |  |  |  |  |  |  |  |  |  |  |  |
| HSV | | 0 (0) | | 0 (0) | | | | 0 (0) | | | - | | | 63·2 | | | | | - | | | | | |  | | | |  | | | | | |  |  |  |  |  |  |  |  |  |  |  |  |  |
| VZV | | 4 (5·9) | | 1 (4) | | | | 3 (7) | | | 25 | | | 62·5 | | | | | 0·56 (0·05 to 5·65) | | | | | |  | | | |  |  |  |  |  |  |  |  |  |  |  |  |  |  |  |  |  |  |  |
| Bacterial | | 3(4·4) | | 0 (0) | | | | 3 (7) | | | 0 | | | 61·5 | | | | | 0·57 (0·16 to 2·04) | | | | | |  | | | |  |  |  |  |  |  |  |  |  |  |  |  |  |  |  |  |  |  |  |
| ADEM/immune | | 7 (10·3) | | 3 (12) | | | | 4 (9·3) | | | 42·9 | | | 63·9 | | | | | 1·33 (0·27 to 6·49) | | | | | |  | | | |  |  |  |  |  |  |  |  |  |  |  |  |  |  |  |  |  |  |  |
| Mycobacterium TB | | 10 (14·7) | | 5 (20) | | | | 5 (11·6) | | | 50 | | | 65·5 | | | | | 1·90 (0·49 to 7·35) | | | | | |  | | | |  | | | |  | | | | | | | | | | | | |  |  |
| Infection (other) | | 23 (33·8) | | 6 (24) | | | | 17 (39·5) | | | 26·1 | | | 57·8 | | | | | 0·75 (0·16 to 2·04) | | | | | |  | | | |  | | | |  |  |  |  |  |  |  |  |  |  |  |  |  |  |  |
| Unknown | | 20 (29·4) | | 9 (36) | | | | 11 (25·6) | | | 45 | | | 66·7 | | | | | **-** | | | | | |  | | | |  | | | |  |  |  |  |  |  |  |  |  |  |  |  |  |  |  |
| Immunosuppressed, n (%) | | 24 (35·3) | | 9 (36) | | | | 15 (34·9) | | | 37·5 | | | 63·6 | | | | | 0·40 (0·05 to 3·20) | | | | | | 0·70 | | | | >0·99 | | | |  |  |  |  |  |  |  |  |  |  |  |  |  |  |  |
| HIV positive, n (%) | | 6 (8·8) | | 1 (4) | | | | 5 (11·6) | | | 16·7 | | | 61·3 | | | | | 0·40 (0·05 to 3·20) | | | | | | 0·70 | | | | >0·99 | | | |  |  |  |  |  |  |  |  |  |  |  |  |  |  |  |
|  | |  | |  | | | |  | | |  | | |  | | | | |  | | | | | |  | | | |  | | | |  |  |  |  |  |  |  |  |  |  |  |  |  |  |  |
|  | |  | |  | | | |  | | |  | | |  | | | | |  | | | | | |  | | | |  | | | |  |  |  |  |  |  |  |  |  |  |  |  |  |  |  |
| **Symptoms**  Median symptom duration prior to admission, days (IQR, range) | | 3 (2-9·25, 1-60) | | 3 (1-14, 1-42) | | | | 2 (2-5, 1-60) | | |  | | |  | | | | |  | | | | | | 0·74 | | | | >0·99 | | | |  |  |  |  |  |  |  |  |  |  |  |  |  |  |  |
| GCS at presentation, n/total | |  | |  | | | |  | | |  | | |  | | | | |  | | | | | |  | | | |  | | | |  | | | | | | |  | | |  |  |  |  |  |
| ≤ 8 | | 8/68 | | 5/25 | | | | 3/43 | | | 62·5 | | | 66·7 | | | | | 3·89 (0·71 to 16·54) | | | | | | 0·12 | | | | >0·99 | | | |  |  |  |  |  |  |  |  |  |  |  |  |  |  |  |
| 9-12. | | 8/68 | | 5/25 | | | | 3/43 | | | 62·5 | | | 66·7 | | | | | 3·89 (0·71 to 16·54) | | | | | | 0·12 | | | | >0·99 | | | |  |  |  |  |  |  |  |  |  |  |  |  |  |  |  |
| 13-14 | | 22/68 | | 6/25 | | | | 16/43 | | | 27·3 | | | 58·7 | | | | | 0·86 (0·26 to 2·72) | | | | | | >0·99 | | | | >0·99 | | | |  |  |  |  |  |  |  |  |  |  |  |  |  |  |  |
| 15 (ref) | | 28/68 | | 9/25 | | | | 21/43 | | | 30 | | | 57·9 | | | | | - | | | | | | - | | | | - | | | |  | | | | | | |  | | |  |  |  |  |  |
| Temperature over 38°C on admission, n/total | | 5/67 | | 3/24 | | | | 2/43 | | | 60 | | | 66·1 | | | | | 2·93 (0·45 to 18·90) | | | | | | 0·34 | | | | >0·99 | | | |  | | | | | | |  | | |  |  |  |  |  |
| **Presenting symptoms, n (%)**  Fever | | 42 (61·8) | | 14 (56·0) | | | | 28 (65·1) | | | 33·3 | | | 57·7 | | | | | 0·68 (0·25 to 1·87) | | | | | | 0·60 | | | | >0·99 | | | |  |  |  |  |  |  |  |  |  |  |  |  |  |  |  |
| Headache | | 25 (36·8) | | 6 (24·0) | | | | 19 (44·2) | | | 24 | | | 55·8 | | | | | 0·40 (0·13 to 1·20) | | | | | | 0·12 | | | | >0·99 | | | |  |  |  |  |  |  |  |  |  |  |  |  |  |  |  |
| Lethargy | | 23 (33·8) | | 6 (24·0) | | | | 17 (39·5) | | | 26·1 | | | 57·8 | | | | | 2·96 (1·03 to 8·48) | | | | | | 0·06 | | | | >0·99 | | | |  |  |  |  |  |  |  |  |  |  |  |  |  |  |  |
| Seizure | | **33 (48·5)** | | **25 (100·0)** | | | | **8 (18·6)** | | | **75·8** | | | **100** | | | | | **-** | | | | | | ***** <0·0001** | | | | ****0·0031** | | | |  |  |  |  |  |  |  |  |  |  |  |  |  |  |  |
| Behavioural change | | 29 (42·6) | | 11 (44·0) | | | | 18 (41·9) | | | 37·9 | | | 64·1 | | | | | 1·09 (0·40 to 2·95) | | | | | | *0·05 | | | | >0·99 | | | |  |  |  |  |  |  |  |  |  |  |  |  |  |  |  |
| Focal neurology | | 15 (22·1) | | 6 (24·0) | | | | 9 (20·9) | | | 40 | | | 64·2 | | | | | 1·19 (0·37 to 3·87) | | | | | | 0·77 | | | | >0·99 | | | |  |  |  |  |  |  |  |  |  |  |  |  |  |  |  |
| Irritability | | 9 (13·2) | | 3 (12·0) | | | | 6 (14·0) | | | 33·3 | | | 62·7 | | | | | 0·84 (0·19 to 3·70) | | | | | | >0·99 | | | | >0·99 | | | |  |  |  |  |  |  |  |  |  |  |  |  |  |  |  |
| Neck stiffness | | 13 (19·1) | | 5 (20·0) | | | | 8 (18·6) | | | 38·5 | | | 63·6 | | | | | 1·09 (0·31 to 3·80) | | | | | | >0·99 | | | | >0·99 | | | |  |  |  |  |  |  |  |  |  |  |  |  |  |  |  |
| Photophobia | | - | | - | | | | - | | | - | | | - | | | | | - | | | | | | - | | | |  | | | |  |  |  |  |  |  |  |  |  |  |  |  |  |  |  |
| Gastrointestinal | | 14 (20·9) | | 2 (8·0) | | | | 12 (28·6) | | | 14·3 | | | 56·6 | | | | | 0·22 (0·04 to 0·91) | | | | | | 0·063 | | | | >0·99 | | | |  |  |  |  |  |  |  |  |  |  |  |  |  |  |  |
| Respiratory | | 10 (14·7) | | 0 (0·0) | | | | 10 (23·3) | | | 0 | | | 56·9 | | | | | - | | | | | | *0·01 | | | | 0·32 | | | |  |  |  |  |  |  |  |  |  |  |  |  |  |  |  |
| **Diagnostic tests** | |  | |  | | | |  | | |  | | |  | | | | |  | | | | | |  | | | |  | | | |  |  |  |  |  |  |  |  |  |  |  |  |  |  |  |
| First MRI, n/total | |  | |  | | | |  | | |  | | |  | | | | |  | | | | | |  | | | |  | | | |  |  |  |  |  |  |  |  |  |  |  |  |  |  |  |
| Abnormal | | 45/68 | | 16/25 | | | | 29·43 | | | 35·6 | | | 60·9 | | | | | 0·86 (0·30 to 4·42) | | | | | | 0·80 | | | | >0·99 | | | |  |  |  |  |  |  |  |  |  |  |  |  |  |  |  |
| First EEG, n/total | |  | |  | | | |  | | |  | | |  | | | | |  | | | | | |  | | | |  | | | |  |  |  |  |  |  |  |  |  |  |  |  |  |  |  |
| Focal abnormality | | 9/59 | | 5/25 | | | | 4/34 | | | 55·6 | | | 60 | | | | | 3·31 (0·58 to 18·85) | | | | | | 0·38 | | | | >0·99 | | | |  |  |  |  |  |  |  |  |  |  |  |  |  |  |  |
| Consistent with encephalitis | | 36/59 | | 16/25 | | | | 20/34 | | | 44·4 | | | 60·9 | | | | | 2·00 (0·57 to 6·56) | | | | | | 0·35 | | | | >0·99 | | | |  |  |  |  |  |  |  |  |  |  |  |  |  |  |  |
| Low CSF:serum glucose ratio, n/total | | 29/61 | | 11/22 | | | | 18/39 | | | 37·9 | | | 64·1 | | | | | 0·66 (0·20 to 2·21) | | | | | | 0·57 | | | | >0·99 | | | |  |  |  |  |  |  |  |  |  |  |  |  |  |  |  |
| CSF:serum glucose ratio, mean | | 0·63 (0·24) | | 0·68 (0·20) | | | | 0·61 (0·26) | | | **-** | | | **-** | | | | | **-** | | | | | | 0·29 | | | | >0·99 | | | |  |  |  |  |  |  |  |  |  |  |  |  |  |  |  |
| Low initial serum sodium, n/total | | 3/68 | | 1/25 | | | | 2/43 | | | 33·3 | | | 63·1 | | | | | 0·85 (0·07 to 9·93) | | | | | | >0·99 | | | | >0·99 | | | |  |  |  |  |  |  |  |  |  |  |  |  |  |  |  |
| Initial serum sodium, mmol/L, mean | | 136·4 (6·26) | | 138·2 (5·33) | | | | 135·3 (6·64) | | | - | | | - | | | | | - | | | | | | 0·10 | | | | >0·99 | | | |  |  |  |  |  |  |  |  |  |  |  |  |  |  |  |
| High initial serum lactate, n/total | | 1/18 | | 0/2 | | | | 1/16 | | | 0 | | | 88·2 | | | | | - | | | | | | >0·99 | | | | >0·99 | | | |  |  |  |  |  |  |  |  |  |  |  |  |  |  |  |
| Initial serum lactate, mmol/L, mean | | 2·37 (0·80) | | 1·90 (-) | | | | 2·42 (2·75) | | | **-** | | | **-** | | | | | **-** | | | | | | 0·40 | | | | >0·99 | | | |  |  |  |  |  |  |  |  |  |  |  |  |  |  |  |
| Taking anti-seizure medication prior to admission, n/total | | 2/68 | | 2/25 | | | | 0/43 | | | 60·0 | | | 66·1 | | | | | - | | | | | | 0·20 | | | | >0·99 | | | |  |  |  |  |  |  |  |  |  |  |  |  |  |  |  |

*Table S9:* A summary of the Western Pacific region study group separated in patients that did and did not have a seizure with univariate analysis.

Analysis completed using student’s t-test or fisher’s exact test.

Bold font highlight significant differences (* p<0·05; ** p<0·01; *** p<0·001) after Bonferroni correction for multiple analyses, shown as adjusted p-value.

*Abbreviations: acute disseminating encephalomyelitis (ADEM), cerebrospinal fluid (CSF), electroencephalogram (EEG), Glasgow Coma Scale (GCS), Herpes simplex virus (HSV), interquartile range (IQR), magnetic resonance imaging (MRI), negative predictive value (NPV), odds ratio (OR), positive predictive value (PPV), standard deviation (SD), tuberculosis (TB), Varicella zoster virus (VZV).*

| **Variable** | **All (n=525)** | **Seizure (n=201)** | **No seizure (n=324)** | **PPV** | **NPV** | **OR (95% CI)** | **p-value** | **Adjusted p-value** |  |  |  |  |
| --- | --- | --- | --- | --- | --- | --- | --- | --- | --- | --- | --- | --- |
| **Demographic**  Age, years, median (IQR) | 55·5 (37-70) | 60 (43-70) | 58 (42-72) |  |  |  | 0·66 | >0·99 |  |  |  |  |
| Age, years, n (%)  ≤ 5 | 3(0·5) | 3 (1·5) | 0 (0) | 100 | 62·1 | - | 0·10 | >0·99 |  |  |  |  |
| >5 to ≤ 18 | 10 (1·9) | 6 (3) | 4 (1·2) | 60 | 62·1 | 2·46 (0·69 to 8·83) |  |  |  |  |  |  |
| >18 to ≤ 40 | 101 (17·2) | 33 (16·4) | 66 (20·4) | 33·3 | 60·6 | 0·77 (0·48 to 1·22) |  |  |  |  |  |  |
| >40 to ≤ 60 | 164 (28) | 61 (30·3) | 102 (31·5) | 37·4 | 61·3 | 0·95 (0·65 to 1·39) |  |  |  |  |  |  |
| >60 | 252 (43) | 98 (48·8) | 152 (46·9) | 39·2 | 62·5 | 1·08 (0·76 to 1·53) |  |  |  |  |  |  |
| Sex, n (%)  Male | 301 (51·4) | 109 (54·2) | 190 (58·6) | 36·5 | 59·3 | 0·84 (0·59 to 1·19) | 0·36 | >0·99 |  |  |  |  |
| **Aetiology, n (%)**  Autoantibody-associated | **57 (9·7)** | **31 (15·4)** | **23 (7·1)** | **57·4** | **63·9** | **2·39 (1·35 to 4·23)** | *****<0·0001** | ****0·0032** |  |  |  |  |
| HSV | **46 (7·8)** | **26 (12·9)** | **19 (5·9)** | **57·8** | **63·5** | **2·38 (1·28 to 4·43)** |  |  |  |  |  |  |
| VZV | **19 (3·2)** | **3 (1·5)** | **16 (4·9)** | **15·8** | **60·9** | **0·29 (0·08 to 1·01)** |  |  |  |  |  |  |
| Bacterial | **36 (6·1)** | **7 (3·5)** | **29 (9)** | **19·4** | **60·3** | **0·37 (0·16 to 0·85)** |  |  |  |  |  |  |
| ADEM/immune | **72 (12·3)** | **31 (15·4)** | **41 (12·7)** | **43·1** | **62·5** | **1·26 (0·76 to 2·08)** |  |  |  |  |  |  |
| Mycobacterium TB | **3 (0·5)** | **1 (0·5)** | **2 (0·6)** | **33·3** | **61·7** | **0·81 (0·07 to 8·94)** |  |  | |  | | |
| Infection (other) | **240 (41)** | **76 (37·8)** | **164 (50·6)** | **31·7** | **56·1** | **0·59 (0·41 to 0·85)** |  |  | |  |  |  |
| Unknown | **53 (9)** | **24 (11·9)** | **28 (8·6)** | **46·2** | **62·6** | **1·43 (0·81 to 2·55)** |  |  | |  |  |  |
| Immunosuppressed, n (%) | 61 (10·4) | 20 (10) | 41 (12·7) | 32·8 | 61·0 | 0·76 (0·43 to 1·34) | 0·40 | >0·99 | |  |  |  |
| HIV positive, n (%) | 3 (0·5) | 0 (0·0) | 3 (0·9) | 0·0 | 61·5 | - | 0·29 | >0·99 | |  |  |  |
|  |  |  |  |  |  |  |  |  | |  |  |  |
| **Symptoms**  Median symptom duration prior to admission, days (IQR, range) | 2 (1-5, 1-4362) | 2 (1-5·75, 1-4362) | 2 (1-4, 1-360) |  |  |  | 0·12 | >0·99 | |  |  |  |
| GCS at presentation, n/total |  |  |  |  |  |  |  |  | |  |  |  |
| ≤ 8 | **83/525** | **48/198** | **34/324** | **58·5** | **65·5** | **3·18 (1·84 to 5·46)** | ***** <0·0001** | ****0·0032** | |  |  |  |
| 9-12. | 98/525 | 41/198 | 56/324 | 42·3 | 62·6 | 1·65 (0·99 to 2·72) | 0·067 | >0·99 | |  |  |  |
| 13-14 | 151/525 | 50/198 | 101/324 | 33·1 | 59·6 | 1·12 (0·70 to 1·77) | 0·64 | >0·99 | |  |  |  |
| 15 (ref) | 195/525 | 59/198 | 133/324 | 30·7 | 57·4 | - | - | - | |  |  |  |
| Temperature over 38°C on admission, n/total | 83/499 | 27/174 | 56/275 | 32·5 | 60·8 | 0·75 (0·45 to 1·23) | 0·27 | >0·99 | |  |  |  |
| **Presenting symptoms, n (%)**  Fever | 189 (36·1) | 62 (30·8) | 125 (38·7) | 33·2 | 58·8 | 0·71 (0·49 to 1·03) | 0·075 | >0·99 | |  |  |  |
| Headache | 188 (36·6) | 57 (29·8) | 130 (40·4) | 30·5 | 58·9 | 0·63 (0·43 to 0·92) | * 0·018 | 0·58 | |  |  |  |
| Lethargy | 251 (47·8) | 103 (51·2) | 147 (45·4) | 41·2 | 64·4 | 2·99 (0·81 to 11·10) | 0·21 | >0·99 | |  |  |  |
| Seizure | **194 (38·1)** | **172 (90·5)** | **17 (5·3)** | **91·0** | **94·4** | **169·75 (82·24 to 338·03)** | *****<0·0001** | ****0·0032** | |  |  |  |
| Behavioural change | 359 (69·0) | 128 (65·3) | 229 (70·7) | 35·9 | 58·3 | 0·78 (0·53 to 1·14) | 0·21 | >0·99 | |  |  |  |
| Focal neurology | 153 (29·5) | 56 (28·7) | 97 (29·9) | 36·6 | 62·0 | 0·94 (0·64 to 1·39) | 0·84 | >0·99 | |  |  |  |
| Irritability | 200 (38·9) | 67 (34·9) | 132 (41·0) | 33·7 | 60·3 | 0·77 (0·53 to 1·12) | 0·19 | >0·99 | |  |  |  |
| Neck stiffness | 146 (28·9) | 46 (24·2) | 100 (31·6) | 31·5 | 60·0 | 0·69 (0·46 to 1·04) | 0·085 | >0·99 | |  |  |  |
| Photophobia | 45 (11·5) | 20 (13·3) | 25 (10·3) | 44·4 | 62·5 | 1·34 (0·71 to 2·50) | 0·42 | >0·99 | |  |  |  |
| Gastrointestinal | 47 (9·12) | 18 (9·13) | 29 (9·12) | 38·3 | 61·8 | 1·00 (0·54 to 1·86) | >0·99 | >0·99 | |  |  |  |
| Respiratory | 40 (7·66) | 12 (6·00) | 28 (8·70) | 30·0 | 61·0 | 0·67 (0·33 to 1·35) | 0·31 | >0·99 | |  |  |  |
| **Diagnostic tests** |  |  |  |  |  |  |  |  | |  |  |  |
| First MRI, n/total |  |  |  |  |  |  |  |  | |  |  |  |
| Abnormal | 223/500 | 99/191 | 121/309 | 45·0 | 67·1 | 1·67 (1·16 to 2·41) | 0·64 | >0·99 | |  |  |  |
| First EEG, n/total |  |  |  |  |  |  |  |  | |  |  |  |
| Focal abnormality | **154/466** | **121/188** | **31/278** | **79·6** | **78·7** | **96·38 (44·77 to 217·10)** | *****<0·0001** | ****0·0032** | |  |  |  |
| Consistent with encephalitis | **126/466** | **60/188** | **64/278** | **48·4** | **62·6** | **24·51 (10·51 to 58·05)** | *****<0·0001** | ****0·0032** | |  |  |  |
| Low CSF:serum glucose ratio, n/total | 158/493 | 48/187 | 109/306 | 30·6 | 58·6 | 0·62 (0·42 to 0·93) | * 0·022 | 0·70 | |  |  |  |
| CSF:serum glucose ratio, mean (SD) | 0·55 (0·15) | 0·58 (0·13) | 0·53 (0·15) | - | - | - | **0·0020 | 0·064 | |  |  |  |
| Low initial serum sodium, n/total | 4/465 | 3/169 | 1/296 | 75·0 | 64·0 | 5·33 (0·55 to 51·67) | 0·14 | >0·99 | |  |  |  |
| Initial serum sodium, mmol/L, mean (SD) | 138·7 (8·41) | 137·6 (11·55) | 139·4 (5·87) | - | - | - | *0·023 | 0·66 | |  |  |  |
| High initial serum lactate, n/total | **10/292** | **8/86** | **2/206** | **80·0** | **72·3** | **10·46 (2·17 to 50·35)** | ****0·0011** | ***0·035** | |  |  |  |
| Initial serum lactate, mmol/L, mean (SD) | **1·56 (1·33)** | **2·16 (2·03)** | **1·32 (0·78)** | **-** | **-** | **-** | *****<0·0001** | ****0·0032** | |  |  |  |
| Taking anti-seizure medication prior to admission, n/total | 8/252 | 5/110 | 3/142 | 62·5 | 57·0 | 2·21 (0·52 to 9·44) | 0·30 | >0·99 | |  |  |  |

*Table S10:* A summary of the Europe study group separated in patients that did and did not have a seizure with univariate analysis.

Analysis completed using student’s t-test or fisher’s exact test.

Bold font highlight significant differences (* p<0·05; ** p<0·01; *** p<0·001) after Bonferroni correction for multiple analyses, shown as adjusted p-value.

*Abbreviations: acute disseminating encephalomyelitis (ADEM), cerebrospinal fluid (CSF), electroencephalogram (EEG), Glasgow Coma Scale (GCS), Herpes simplex virus (HSV), interquartile range (IQR), magnetic resonance imaging (MRI), negative predictive value (NPV), odds ratio (OR), positive predictive value (PPV), standard deviation (SD), tuberculosis (TB), Varicella zoster virus (VZV).*

Figure S1- Receiver operating curves (ROC) for the performance of the SEIZURE score for study groups within Europe with area under the curve calculated and sample sizes documented.

Abbreviations- area under the receiver operating curve (AUROC)

| **Variable** | **All (n=151)** | **Seizure (n=83)** | | **No seizure (n=68)** | **PPV** | **NPV** | **OR (95% CI)** | **p-value** | **Adjusted p-value** |
| --- | --- | --- | --- | --- | --- | --- | --- | --- | --- |
| **Demographic**  Age, years, median (IQR) | 61 (43-71) | 63 (43-69·5) | | 56·5 (44·25-71) |  |  |  | 0·97 | >0·99 |
| Age, years, n (%)  ≤ 5 | 3 (2·0) | 3 (3·6) | | 0 (0·0) | 100 | 45·6 | - | 0·35 | >0·99 |
| >5 to ≤ 18 | 4 (2·7) | 2 (2·4) | | 2 (3·0) | 50·0 | 44·5 | 0·80 (0·11 to 5·85) |  |  |
| >18 to ≤ 40 | 22 (14·7) | 10 (12·0) | | 12 (17·9) | 45·5 | 43·0 | 0·50 (0·16 to 1·54) |  |  |
| >40 to ≤ 60 | 44 (29·3) | 22 (26·5) | | 22 (32·8) | 50·0 | 42·5 | 0·74 (0·36 to 1·49) |  |  |
| >60 | 76 (50·7) | 46 (55·4) | | 30 (44·8) | 60·5 | 50·0 | 1·53 (0·80 to 2·93) |  |  |
| Sex, n (%)  Male | 78 (50·3) | 39 (47·0) | | 39 (58·2) | 50·0 | 38·9 | 0·64 (0·33 to 1·22) | 0·19 | >0·99 |
| **Aetiology, n (%)**  Autoantibody-associated | **29 (19·3)** | **22 (26·5)** | | **7 (10·4)** | **75·9** | **49·6** | **3·09 (1·23 to 7·77)** | *****<0·0001** | ****<0·0032** |
| HSV | **13 (8·7)** | **13 (15·7)** | | **0 (0·0)** | **100·0** | **48·9** | **-** |  |  |
| VZV | **5 (3·3)** | **1 (1·2)** | | **4 (6·0)** | **20·0** | **43·4** | **0·19 (0·02 to 1·76)** |  |  |
| Bacterial | **17 (11·3)** | **3 (3·6)** | | **14 (20·9)** | **17·6** | **39·8** | **0·14 (0·04 to 0·52)** |  |  |
| ADEM/immune | **32 (20·6)** | **18 (21·7)** | | **14 (20·9)** | **56·3** | **44·9** | **1·05 (0·48 to 2·30)** |  |  |
| Mycobacterium TB | **0 (0·0)** | **0 (0·0)** | | **0 (0·0)** | **-** | **44·7** | **-** |  |  |
| Infection (other) | **27 (17·4)** | **8 (9·6)** | | **19 (28·4)** | **29·6** | **39·0** | **0·27 (0·11 to 0·66)** |  |  |
| Unknown | **26 (17·3)** | **18·0 (21·7)** | | **8·0 (11·9)** | **69·2** | **47·6** | **2·04 (0·82 to 5·05)** |  |  |
| Immunosuppressed, n (%) | 21 (13·5) | 7 (8·4) | | 14 (20·9) | 33·3 | 41·1 | 0·35 (0·13 to 0·92) | *0·036 | >0·99 |
| HIV positive, n (%) | 1 (0·6) | 0 (0·0) | | 1 (1·5) | 0·0 | 44·3 | - | 0·45 | >0·99 |
|  |  |  | |  |  |  |  |  |  |
| **Symptoms**  Median symptom duration prior to admission, days (IQR, range) | 14 (5·25-32, 1-4362) | | 15 (3·5-40, 1-4362) | 12 (1-30, 1-360) |  |  |  | 0·32 | >0·99 |
| GCS at presentation, n/total |  | |  |  |  |  |  |  |  |
| ≤ 8 | 29/151 | | 27/83 | 2/68 | 93·1 | **53·7** | **22·78 (5·346 to 100·1)** | *****<0·0001** | ****<0·0032** |
| 9-12. | 15/151 | | 11/83 | 4/68 | 73·3 | 46·7 | 4·64 (1·41 to 13·96) | *0·012 | 0·38 |
| 13-14 | 21/151 | | 13/83 | 8/68 | 68·4 | 46·6 | 3·66 (1·25 to 10·70) | 0·050 | >0·99 |
| 15 (ref) | 86/151 | | 32/83 | 54/68 | 37·2 | 20·3 | - | - | - |
| Temperature over 38°C on admission, n/total | 24/101 | | 12/67 | 12/34 | 50·0 | 43·1 | 0·76 (0·32 to 1·82) | 0·65 | >0·99 |
| **Presenting symptoms, n (%)**  Fever | **68 (45·9)** | **27 (32·9)** | | **39 (59·1)** | **40·9** | **32·9** | **0·34 (0·17 to 0·67)** | ****0·0010** | *** 0·032** |
| Headache | 40 (28·6) | 12 (16·2) | | 27 (40·9) | 30·8 | 38·6 | 0·28 (0·13 to 0·62) | **0·0024 | 0·079 |
| Lethargy | 73 (49·3) | 48 (58·5) | | 24 (36·4) | 66·7 | 55·3 | 2·47 (1·27 to 4·81) | *0·022 | 0·70 |
| Seizure | **81 (55·1)** | **71 (87·7(** | | **5 (7·6)** | **93·4** | **85·9** | **86·72 (28·07 to 267·28)** | *****<0·001** | ****<0·01** |
| Behavioural change | **87 (57·6)** | **51 (65·4)** | | **32 (48·5)** | **60·0** | **54·2** | **1·78 (0·89 to 3·43)** | **0·13** | >0·99 |
| Focal neurology | 61 (42·2) | 33 (42·3) | | 26 (39·4) | 55·9 | 47·1 | 1·13 (0·58 to 2·20) | 0·74 | >0·99 |
| Irritability | **32 (23·0)** | **23 (31·1)** | | **8 (12·3)** | **74·2** | **52·8** | **3·21 (1·32 to 7·82)** | ****0·0085** | 0·27 |
| Neck stiffness | 15 (11·3) | 8 (11·0) | | 7 (13·6) | 53·3 | 44·9 | 0·93 (0·32 to 2·74) | >0·99 | >0·99 |
| Photophobia | 3 (3·5) | 2 (3·8) | | 1 (2·9) | 66·7 | 39·8 | 1·32 (0·12 to 15·15) | >0·99 | >0·99 |
| Gastrointestinal | 18 (12·2) | 9 (11·0) | | 9 (13·6) | 50·0 | 43·8 | 0·78 (0·29 to 2·09) | 0·62 | >0·99 |
| Respiratory | 7 (4·7) | 2 (2·4) | | 5 (7·6) | 28·6 | 43·3 | 0·31 (0·06 to 1·63) | 0·24 | >0·99 |
| **Diagnostic tests** |  |  | |  |  |  |  |  |  |
| First MRI, n/total |  |  | |  |  |  |  |  |  |
| Abnormal | 106/144 | 64/80 | | 39/64 | 62·1 | 61·0 | 2·56 (1·22 to 5·39) | *0·028 | 0·90 |
| First EEG, n/total |  |  | |  |  |  |  |  |  |
| Focal abnormality | **44/125** | **34/77** | | **8/48** | **81·0** | **48·2** | **32·83 (19·28 to 55·91)** | ****0·0011** | *** 0·035** |
| Consistent with encephalitis | 66/125 | 40/77 | | 24/48 | 62·5 | 39·3 | 1·08 (0·51 to 2·21) | 0·86 | >0·99 |
| Low CSF:serum glucose ratio, n/total | 31/132 | 9/73 | | 21/59 | 30·0 | 37·3 | 0·25 (0·11 to 0·61) | **0·0032 | 0·10 |
| CSF:serum glucose ratio, mean (SD) | **0·59 (0·15)** | **0·63 (0·11)** | | **0·53 (0·18)** |  |  |  | *****0·0005** | ****0·0015** |
| Low initial serum sodium, n/total | 1/147 | 1/81 | | 0/66 | 100·0 | 45·2 | - | >0·99 | >0·99 |
| Initial serum sodium, mmol/L, mean (SD) | 136·6 (6·25) | 136·4 (16·61) | | 136·9 (4·99) | - | - | - | 0·28 | >0·99 |
| High initial serum lactate, n/total | 4/47 | 4/28 | | 0/19 | 100·0 | 44·2 | - | 0·14 | >0·99 |
| Initial serum lactate, mmol/L, mean (SD) | 2·21 (2·39) | 2·68 (2·97) | | 1·53 (0·42) | - | - | - | 0·11 | >0·99 |
| Taking anti-seizure medication prior to admission, n/total | 8/148 | 5/82 | | 3/66 | 62·5 | 45·0 | 1·36 (0·31 to 5·93) | >0·99 | >0·99 |
|  |  |  | |  |  |  |  |  |  |

*Table S11:* A summary of the Italy study group separated into patients that did and did not have a seizure with univariate analysis.

Analysis completed using student’s t-test or fisher’s exact test.

Bold font highlight significant differences (* p<0·05, **p<0·01, ***p<0·001)

*Abbreviations: acute disseminating encephalomyelitis (ADEM), cerebrospinal fluid (CSF), electroencephalogram (EEG), Herpes simplex virus (HSV), interquartile range (IQR), magnetic resonance imaging (MRI), negative predictive value (NPV), odds ratio (OR), positive predictive value (PPV),standard deviation (SD), tuberculosis (TB), Varicella zoster virus (VZV)*

| **Variable** | **All (n=52)** | **Seizure (n=12)** | | **No seizure (n=40)** | **PPV** | **NPV** | **OR (95% CI)** | **p-value** | | **Adjusted p-value** | |  |  |  |  |
| --- | --- | --- | --- | --- | --- | --- | --- | --- | --- | --- | --- | --- | --- | --- | --- |
| **Demographic**  Age, years, median (IQR) | 69 (51·5-75) | | 64 (49·5 to 68) | 72 (51·5 to 75·25) |  |  |  | | 0·61 | >0·99 | |  |  |  |  |
| Age, years, n (%)  ≤ 5 | 0 (0·0) | 0 (0·0) | | 0 (0·0) | 0·0 | 76·9 | - | | 0·25 | >0·99 | |  |  |  |  |
| >5 to ≤ 18 | 1 (1·9) | 1 (8·3) | | 0 (0) | 100·0 | 78·4 | - | |  |  | |  |  |  |  |
| >18 to ≤ 40 | 8 (15·4) | 2 (16·7) | | 6 (15·0) | 25·0 | 77·3 | 1·13 (0·20 to 6·51) | |  |  | |  |  |  |  |
| >40 to ≤ 60 | 11 (21·2) | 1 (8·3) | | 10 (25·0) | 9·1 | 73·2 | 0·27 (0·03 to 2·39) | |  |  | |  |  |  |  |
| >60 | 32 (61·5) | 8 (66·7) | | 24 (60·0) | 25·0 | 80·0 | 1·33 (0·34 to 5·18) | |  |  | |  |  |  |  |
| Sex, n (%)  Male | 31 (59·6) | 7 (58·3) | | 24 (60·0) | 25·0 | 80·0 | 0·93 (0·25 to 3·46) | | >0·99 | >0·99 | |  |  |  |  |
| **Aetiology, n (%)**  Autoantibody-associated | 0 (0·0) | 0 (0·0) | | 0 (0·0) | 0·0 | 76·9 | - | | **0·009 | 0·28 | |  |  |  |  |
| HSV | 7 (13·5) | 5 (41·7) | | 2 (5·0) | 71·4 | 84·4 | 13·57 (2·18 to 84·37) | |  |  | |  |  |  |  |
| VZV | 8 (15·4) | 1 (8·3) | | 7 (17·5) | 12·5 | 75·0 | 0·43 (0·05 to 3·88) | |  | |  |  |  |  |  |
| Bacterial | 16 (30·8) | 3 (25·0) | | 13 (32·5) | 18·8 | 75·0 | 0·69 (0·16 to 2·99) | |  | |  |  |  |  |  |
| ADEM/immune | 7 (13·5) | 2 (16·7) | | 5 (12·5) | 28·6 | 77·8 | 1·40 (0·24 to 8·34) | |  | |  |  |  |  |  |
| Mycobacterium TB | 1 (1·9) | 1 (8·3) | | 0 (0·0) | 100·0 | 78·4 | - | |  | |  | |  | | |
| Infection (other) | 7 (13·5) | 0 (0·0) | | 7 (17·5) | 0·0 | 73·3 | - | |  | |  | |  |  |  |
| Unknown | 6 (11·5) | 0 (0·0) | | 6 (15·0) | 0·0 | 73·9 | - | |  | |  | |  |  |  |
| Immunosuppressed, n (%) | 11 (21·2) | 3 (25·0) | | 8 (20·0) | 27·3 | 78·0 | 1·33 (0·29 to 6·09) | | 0·70 | | >0·99 | |  |  |  |
| HIV positive, n (%) | 2 (3·8) | 0 (0·0) | | 2 (5·0) | 0·0 | 76·0 | - | | >0·99 | | >0·99 | |  |  |  |
|  |  |  | |  |  |  |  | |  | |  | |  |  |  |
| **Symptoms**  Median symptom duration prior to admission, days (IQR, range) | 3 (2-5, 1-30) | 2 (1-5·5, 1-21) | | 3 (1-5, 1-30) |  |  |  | | 0·97 | | >0·99 | |  |  |  |
| GCS at presentation, n/total |  |  | |  |  |  |  | |  | |  | |  |  |  |
| ≤ 8 | 3/52 | 2/12 | | 1/40 | 66·7 | 79·6 | 3·00 (0·19 to 56·04) | | >0·99 | | >0·99 | |  |  |  |
| 9-12. | 12/52 | 3/12 | | 9/40 | 25·0 | 77·5 | 0·50 (0·07 to 4·04) | | 0·60 | | >0·99 | |  |  |  |
| 13-14 | 32/52 | 5/12 | | 27/40 | 15·6 | 65·0 | 0·28 (0·04 to 1·96) | | 0·23 | | >0·99 | |  |  |  |
| 15 (ref) | 5/52 | 2/12 | | 3/40 | 40·0 | 78·7 | - | | - | |  | |  |  |  |
| Temperature over 38°C on admission, n/total | 17/52 | 3/12 | | 14/40 | 17·6 | 41·3 | 0·62 (0·14 to 2·66) | | 0·73 | | >0·99 | |  |  |  |
| **Presenting symptoms, n (%)**  Fever | 37 (71·2) | 9 (75·0) | | 28 (70·0) | 24·3 | 80·0 | 1·29 (0·30 to 5·60) | | >0·99 | | >0·99 | |  |  |  |
| Headache | 24 (48·0) | 5 (41·7) | | 19 (50·0) | 20·8 | 73·1 | 0·71 (0·19 to 2·65) | | 0·74 | | >0·99 | |  |  |  |
| Lethargy | 37 (71·2) | 9 (75·0) | | 28 (70·0) | 24·3 | 80·0 | 1·29 (0·30 to 5·60) | | >0·99 | | >0·99 | |  |  |  |
| Seizure | **7 (13·5)** | **6 (50·0)** | | **1 (2·5)** | **85·7** | 86·7 | 39·00 (3·97 to 383·15) | | *****0·0003** | | ****0·0093** | |  |  |  |
| Behavioural change | 39 (75·0) | 7 (58·3) | | 32 (80·0) | 17·9 | 61·5 | 0·35 (0·09 to 1·40) | | 0·10 | | >0·99 | |  |  |  |
| Focal neurology | 17 (32·7) | 3 (25·0) | | 14 (35·0) | 17·6 | 74·3 | 0·62 (0·14 to 2·66) | | 0·51 | | >0·99 | |  |  |  |
| Irritability | **12 (23·1)** | **0 (0·0)** | | **12 (30·0)** | **0·0** | 70·0 | **-** | | ***0·047** | | >0·99 | |  |  |  |
| Neck stiffness | 15 (29·4) | 2 (18·2) | | 13 (32·5) | 13·3 | 75·0 | 0·46 (0·09 to 2·45) | | 0·46 | | >0·99 | |  |  |  |
| Photophobia | 2 (9·1) | 1 (25·0) | | 1 (5·6) | 50·0 | 85·0 | 5·67 (0·27 to 117·45) | | 0·35 | | >0·99 | |  |  |  |
| Gastrointestinal | 6 (11·5) | 2 (16·7) | | 4 (10·0) | 33·3 | 78·3 | 1·80 (0·29 to 11·29) | | 0·62 | | >0·99 | |  |  |  |
| Respiratory | 13 (25·0) | 2 (16·7) | | 11 (27·5) | 15·4 | 74·4 | 0·53 (0·10 to 2·80) | | 0·48 | | >0·99 | |  |  |  |
| **Diagnostic tests** |  |  | |  |  |  |  | |  | |  | |  |  |  |
| First MRI, n/total |  |  | |  |  |  |  | |  | |  | |  |  |  |
| Abnormal | 31/40 | 6/8 | | 25/32 | 19·4 | 77·8 | 0·84 (0·14 to 5·11) | | 0·62 | | >0·99 | |  |  |  |
| First EEG, n/total |  |  | |  |  |  |  | |  | |  | |  |  |  |
| Focal abnormality | 6/31 | 4/10 | | 2/21 | 66·7 | 76·0 | 32·83 (19·28 to 55·91) | | 0·14 | | >0·99 | |  |  |  |
| Consistent with encephalitis | 16/31 | 5/10 | | 11/21 | 31·3 | 66·7 | 6·96 (4·23 to 11·44) | | 0·71 | | >0·99 | |  |  |  |
| Low CSF:serum glucose ratio, n/total | 27/51 | 6/12 | | 21/39 | 22·2 | 75·0 | 0·86 (0·23 to 3·13) | | 0·75 | | >0·99 | |  |  |  |
| CSF:serum glucose ratio, mean (SD) | 0·41 (0·22) | 0·43 (0·16) | | 0·40 (0·23) |  |  |  | | 0·84 | | >0·99 | |  |  |  |
| Low initial serum sodium, n/total | 0/52 | 0/12 | | 0/40 | 0·0 | 76·9 | - | | - | | - | |  |  |  |
| Initial serum sodium, mmol/L, mean (SD) | 136·5 (4·50) | 136·4 (4·56) | | 136·5 (4·56) |  |  |  | | 0·90 | | >0·99 | |  |  |  |
| High initial serum lactate, n/total | 4/44 | 2/11 | | 2/33 | 50·0 | 77·5 | 3·44 (0·42 to 28·01) | | 0·27 | | >0·99 | |  |  |  |
| Initial serum lactate, mmol/L, mean (SD) | 2·12 (1·67) | 2·98 (2·25) | | 1·83 (1·38) |  |  |  | | 0·051 | | >0·99 | |  |  |  |
| Taking anti-seizure medication prior to admission, n/total | 2/51 | 1/12 | | 1/40 | 50·0 | 78·0 | 3·55 (0·20 to 61·38) | | 0·41 | | >0·99 | |  |  |  |
|  |  |  | |  |  |  |  | |  | |  | |  |  |  |
|  |  |  | |  |  |  |  | |  | |  | |  |  |  |

*Table S12:* A summary of the Spain study group separated into patients that did and did not have a seizure with univariate analysis.

Analysis completed using student’s t-test or fisher’s exact test.

Bold font highlight significant differences (* p<0·05; ** p<0·01; *** p<0·001) after Bonferroni correction for multiple analyses, shown as adjusted p-value.

*Abbreviations: acute disseminating encephalomyelitis (ADEM), cerebrospinal fluid (CSF), electroencephalogram (EEG), Herpes simplex virus (HSV), interquartile range (IQR), magnetic resonance imaging (MRI), negative predictive value (NPV), odds ratio (OR), positive predictive value (PPV), standard deviation (SD), tuberculosis (TB), Varicella zoster virus (VZV)*

| **Variable** | **All (n=50)** | | **Seizure (n=15)** | **No seizure (n=35)** | **PPV** | **NPV** | **OR (95% CI)** | **p-value** | **Adjusted p-value** |
| --- | --- | --- | --- | --- | --- | --- | --- | --- | --- |
| **Demographic**  Age, years, median (IQR) | 63 (48-71) | 52 (36-66) | | 67 (52·5-76) | - | - | - | *0·031 | 0·93 |
| Age, years, n (%)  ≤ 5 | 0 (0) | 0 (0) | | 0 (0) | 0 | 70·0 |  | 0·27 | >0·99 |
| >5 to ≤ 18 | 2 (4·0) | 1 (6·7) | | 1 (2·9) | 50·0 | 70·8 | 2·43 (0·14 to 41·60) |  |  |
| >18 to ≤ 40 | 6 (12·0) | 3 (20·0) | | 3 (8·6) | 50·0 | 72·7 | 2·67 (0..47 to 15·08) |  |  |
| >40 to ≤ 60 | 16 (32·0) | 6 (40·0) | | 10 (28·6) | 37·5 | 73·5 | 1·67 (0·47 to 5·92) |  |  |
| >60 | 25 (52·0) | 5 (33·3) | | 21 (60·0) | 19·2 | 58·3 | 0·33 (0·09 to 1·19) |  |  |
| Sex, n (%)  Male | 25 (50·0) | 6 (40·0) | | 19 (54·3) | 24·0 | 64·0 | 0·56 (0·16 to 1·92) | 0·54 | >0·99 |
| **Aetiology, n (%)**  Autoantibody-associated | 0 (0) | 0 (0) | | 0 (0) | 0 | 69·4 | - | 0·95 | >0·99 |
| HSV | 6 (12·0) | 1 (6·7) | | 5 (14·3) | 16·7 | 68·2 | 0·43 (0·05 to 4·02) |  |  |
| VZV | 1 (2·0) | 0 (0·0) | | 1 (2·9) | 0·0 | 69·4 | **-** |  |  |
| Bacterial | 2 (4·0) | 1 (6·7) | | 1 (2·9) | 50·0 | 70·8 | 2·43 (0·14 to 41·60) |  |  |
| ADEM/immune | 16 (32·0) | 6 (40·0) | | 10 (28·6) | 37·5 | 73·5 | 1·67 (0·47 to 5·92) |  |  |
| Mycobacterium TB | 2 (4·0) | 0 (0) | | 2 (5·7) | 0 | 68·8 | - |  |  |
| Infection (other) | 7 (14·0) | 2 (13·3) | | 5 (14·3) | 28·6 | 69·8 | 0·92 (0·16 to 5·39) |  |  |
| Unknown | 16 (32·0) | 5 (33·3) | | 11 (31·4) | 31·3 | 70·6 | 1·09 (0·30 to 3·96) |  |  |
| Immunosuppressed, n (%) | 4 (8·0) | 1 (6·7) | | 3 (8·6) | 25·0 | 69·6 | 0·76 (0·07 to 7·98) | >0·99 | >0·99 |
| HIV positive, n (%) | 0 (0) | 0 (0) | | 0 (0) | 0 | 69·4 | - | - | - |
|  |  |  | |  |  |  |  |  |  |
| **Symptoms**  Median symptom duration prior to admission, days (IQR, range) | 4 (2-12, 1-120) | 2 (1·5-3·5, 1-8) | | 6·5 (2·75-14·25, 2-120) |  |  |  | 0·19 | >0·99 |
| GCS at presentation, n/total |  |  | |  |  |  |  |  |  |
| ≤ 8 | **2/47** | **2/12** | | **0/35** | **100·0** | **72·9** | - | ***0·035** | >0·99 |
| 9-12. | 5/47 | 3/12 | | 2/35 | 60·0 | 73·3 | 11·25 (1·33 to 82·58) | 0·055 | >0·99 |
| 13-14 | 23/47 | 5/12 | | 18/35 | 21·7 | 63·0 | 2·08 (0·34 to 11·45) | 0·68 | >0·99 |
| 15 (ref) | 17/47 | 2/12 | | 15/35 | 11·8 | 60·6 | - | - | - |
| Temperature over 38°C on admission, n/total | 13/31 | 4/8 | | 9/23 | 30·8 | 77·8 | 1·56 (0·31 to 7·85) | 0·69 | >0·99 |
| **Presenting symptoms, n (%)**  Fever | 21 (42·0) | 7 (46·7) | | 14 (40·0) | 33·3 | 72·4 | 1·31 (0·39 to 4·44) | 0·76 | >0·99 |
| Headache | 23 (47·9) | 6 (46·2) | | 17 (48·6) | 26·1 | 72·0 | 0·91 (0·25 to 3·25) | >0·99 | >0·99 |
| Lethargy | 44 (88·0) | 12 (80·0) | | 32 (91·4) | 27·3 | 50·0 | 0·38 (0·07 to 2·12) | 0·35 | >0·99 |
| Seizure | **23 (46·0)** | **15 (100·0)** | | **8 (22·9)** | **65·2** | **100·0** | **-** | ***** <0·0001** | ****0·003** |
| Behavioural change | 43 (86·0) | 14 (93·3) | | 29 (82·9) | 32·6 | 85·7 | 2·90 (0·32 to 26·43) | 0·66 | >0·99 |
| Focal neurology | 41 (83·7) | 12 (85·7) | | 29 (82·9) | 29·3 | 75·0 | 1·24 (0·22 to 3·41) | >0·99 | >0·99 |
| Irritability | 14 (28·6) | 4 (26·7) | | 10 (29·4) | 28·6 | 68·6 | 0·87 (0·22 to 3·41) | >0·99 | >0·99 |
| Neck stiffness | 11 (22·9) | 4 (26·7) | | 7 (21·2) | 36·4 | 70·3 | 1·35 (0·33 to 5·57) | 0·71 | >0·99 |
| Photophobia | 5 (10·9) | 1 (7·7) | | 4 (12·1) | 20·0 | 70·7 | 0·60 (0·06 to 5·98) | >0·99 | >0·99 |
| Gastrointestinal | 15 (10·9) | 5 (33·3) | | 10 (29·4) | 33·3 | 70·6 | 1·20 (0·33 to 4·41) | >0·99 | >0·99 |
| Respiratory | 14 (29·2) | 6 (40·0) | | 8 (24·2) | 42·9 | 73·5 | 2·08 (0·57 to 7·68) | 0·32 | >0·99 |
| **Diagnostic tests** |  |  | |  |  |  |  |  |  |
| First MRI, n/total |  |  | |  |  |  |  |  |  |
| Abnormal | 31/46 | 10/14 | | 21/32 | 32·3 | 73·3 | 1·31 (0·33 to 5·15) | >0·99 | >0·99 |
| First EEG, n/total |  |  | |  |  |  |  |  |  |
| Focal abnormality | 12/42 | 5/12 | | 7/30 | 41·7 | 76·7 | 2·34 (0·56 to 8·91) | 0·27 | >0·99 |
| Consistent with encephalitis | 27/42 | 7/12 | | 20/30 | 25·9 | 66·7 | 0·70 (0·19 to 2·73) | 0·73 | >0·99 |
| Low CSF:serum glucose ratio, n/total | 9/40 | 2/11 | | 7/29 | 22·2 | 71·0 | 0·70 (0·12 to 4·03) | >0·99 | >0·99 |
| CSF:serum glucose ratio, mean (SD) | 0·56 (0·15) | 0·55 (0·13) | | 0·56 (0·15) |  |  |  | 0·47 | >0·99 |
| Low initial serum sodium, n/total | 2/46 | 1/12 | | 1/34 | 50·0 | 75·0 | 3·00 (0·17 to 52·10) | 0·46 | >0·99 |
| Initial serum sodium, mmol/L, mean (SD) | 137·1 (6·31) | 137·3 (7·25) | | 137·1 (6·06) |  |  |  | 0·96 | >0·99 |
| High initial serum lactate, n/total | 2/21 | 2/8 | | 0/13 | 100·0 | 68·4 | - | 0·13 | >0·99 |
| Initial serum lactate, mmol/L, mean (SD) | 1·44 (1·26) | 2·01 (1·80) | | 1·09 (0·63) |  |  |  | 0·11 | >0·99 |
| Taking anti-seizure medication prior to admission, n/total | 0/49 | 0/15 | | 0/34 | - | 69·4 | - | **-** | - |
|  |  |  | |  |  |  |  |  |  |
| *Table S13:* A summary of the Portugal study group separated into patients that did and did not have a seizure with univariate analysis.  Analysis completed using student’s t-test or fisher’s exact test.  Bold font highlight significant differences (* p<0·05; ** p<0·01; *** p<0·001) after Bonferroni correction for multiple analyses, shown as adjusted p-value.  *Abbreviations: acute disseminating encephalomyelitis (ADEM), cerebrospinal fluid (CSF), electroencephalogram (EEG), Herpes simplex virus (HSV), interquartile range (IQR), magnetic resonance imaging (MRI), negative predictive value (NPV), odds ratio (OR), positive predictive value (PPV), standard deviation (SD), tuberculosis (TB), Varicella zoster virus (VZV)* |  |  | |  |  |  |  |  |  |

| **Variable** | **All (n=262)** | | **Seizure (n=86)** | **No seizure (n=176)** | **PPV** | **NPV** | **OR (95% CI)** | **p-value** | **Adjusted p-value** |
| --- | --- | --- | --- | --- | --- | --- | --- | --- | --- |
| **Demographic**  Age, years, median (IQR) | 57 (42-70) | 59 (45-70·75) | | 55 (41-68·25) |  |  |  | 0·22 | >0·99 |
| Age, years, n (%)  ≤ 5 | 0 (0·0) | | 0 (0·0) | 0 (0·0) | 0·0 | 67·2 | - | 0·80 | >0·99 |
| >5 to ≤ 18 | 1 (0·4) | | 0 (0·0) | 1 (0·6) | 0·0 | 67·0 | - |  |  |
| >18 to ≤ 40 | 57 (21·8) | | 16 (18·6) | 41 (23·3) | 28·1 | 65·9 | 0·75 (0·39 to 1·44) |  |  |
| >40 to ≤ 60 | 90 (34·4) | | 31 (36·0) | 59 (33·5) | 34·4 | 68·0 | 1·12 (0·65 to 1·92) |  |  |
| >60 | 114 (43·5) | | 39 (45·3) | 75 (42·6) | 34·2 | 68·2 | 1·12 (0·66 to 1·88) |  |  |
| Sex, n (%)  Male | 164 (62·6) | | 57 (66·3) | 107 (60·8) | 34·8 | 70·4 | 1·27 (0·74 to 2·17) | 0·42 | >0·99 |
| **Aetiology, n (%)**  Autoantibody-associated | 21 (8·0) | | 8 (9·3) | 13 (7·4) | 38·1 | 67·6 | 1·29 (0·51 to 3·23) | 0·99 | >0·99 |
| HSV | 19 (7·3) | | 7 (8·1) | 12 (6·8) | 36·8 | 67·5 | 1·21 (0·46 to 3·19) |  |  |
| VZV | 4 (1·5) | | 1 (1·2) | 3 (1·7) | 25·0 | 67·1 | 0·68 (0·07 to 6·62) |  |  |
| Bacterial | 1 (0·4) | | 0 (0·0) | 1 (0·6) | 0·0 | 67·0 | - |  |  |
| ADEM/immune | 15 (5·7) | | 4 (4·7) | 11 (6·3) | 26·7 | 66·8 | 0·73 (0·23 to 2·37) |  |  |
| Mycobacterium TB | 0 (0·0) | | 0 (0·0) | 0 (0·0) | 0·0 | 67·2 | - |  |  |
| Infection (other) | 198 (75·6) | | 65 (75·6) | 133 (75·6) | 32·8 | 67·2 | 1·00 (0·55 to 1·82) |  |  |
| Unknown | 4 (1·5) | | 1 (1·2) | 3 (1·7) | 25·0 | 67·1 | 0·68 (0·07 to 6·62) |  |  |
| Immunosuppressed, n (%) | 23 (8·8) | | 8 (9·3) | 15 (8·5) | 34·8 | 67·4 | 1·10 (0·45 to 2·71) | 0·82 | >0·99 |
| HIV positive, n (%) | 0 (0·0) | | 0 (0·0) | 0 (0·0) | 0·0 | 67·2 | - | - | - |
|  |  | |  |  |  |  |  |  |  |
| **Symptoms**  Median symptom duration prior to admission, days (IQR, range) | 2 (1-3, 1-15) | | 1 (1-2, 1-5) | 2 (1-3, 1-15) |  |  |  | 0·70 | >0·99 |
| GCS at presentation, n/total |  | |  |  |  |  |  |  |  |
| ≤ 8 | 48/262 | | 17/86 | 31/176 | 35·4 | 67·8 | 1·67 (0·73 to 3·78) | 0·23 | >0·99 |
| 9-12. | 64/262 | | 24/86 | 40/176 | 37·5 | 68·7 | 1·83 (0·87 to 3·71) | 0·14 | >0·99 |
| 13-14 | 73/262 | | 26/86 | 47/176 | 35·6 | 68·3 | 1·62 (0·80 to 3·20) | 0·21 | >0·99 |
| 15 (ref) | 77/262 | | 19/86 | 58/176 | 24·7 | 63·8 |  |  |  |
| Temperature over 38°C on admission, n/total | 28/262 | | 8/86 | 20/176 | 28·6 | 66·7 | 0·80 | 0·68 | >0·99 |
| **Presenting symptoms, n (%)**  Fever | 61 (23·3) | | 19 (22·1) | 42 (23·9) | 31·1 | 66·7 | 0·90 (0·49 to 1·68) | 0·88 | >0·99 |
| Headache | 98 (37·4) | | 32 (37·2) | 66 (37·5) | 32·7 | 67·1 | 0·99 (0·58 to 1·68) | >0·99 | >0·99 |
| Lethargy | 95 (36·3) | | 34 (39·5) | 61 (34·7) | 35·8 | 68·9 | 1·23 (0·72 to 2·10) | 0·49 | >0·99 |
| Seizure | **81 (31·5)** | | **80 (98·8)** | **1 (0·6)** | **98·8** | **99·4** | **14000·00 (864·68 to 226673·16)** | *****<0·0001** | ****0·0026** |
| Behavioural change | 183 (69·8) | | 52 (60·5) | 131 (74·4) | 28·4 | 57·0 | 0·53 (0·30 to 0·91) | *0·023 | 0·60 |
| Focal neurology | 28 (10·7) | | 5 (5·8) | 23 (13·1) | 17·9 | 65·4 | 0·41 (0·15 to 1·12) | 0·089 | >0·99 |
| Irritability | 139 (53·1) | | 39 (45·3) | 100 (56·8) | 28·1 | 61·8 | 0·63 (0·38 to 1·06) | 0·088 | >0·99 |
| Neck stiffness | 103 (39·3) | | 32 (37·2) | 71 (40·3) | 31·1 | 66·0 | 0·88 (0·52 to 1·49) | 0·69 | >0·99 |
| Photophobia | 35 (15·4) | | 16 (21·1) | 19 (12·5) | 45·7 | 68·9 | 1·87 (0·90 to 3·88) | 0·12 | >0·99 |
| Gastrointestinal | 8 (3·1) | | 2 (2·4) | 6 (3·5) | 25·0 | 67·1 | 0·68 (0·13 to 3·44) | >0·99 | >0·99 |
| Respiratory | 4 (1·5) | | 1 (1·2) | 3 (1·7) | 25·0 | 67·1 | 0·68 (0·07 to 6·62) | >0·99 | >0·99 |
| **Diagnostic tests** |  | |  |  |  |  |  |  |  |
| First MRI, n/total |  | |  |  |  |  |  |  |  |
| Abnormal | 51/262 | | 18/86 | 33/176 | 35·3 | 67·8 | 1·15 (0·60 to 2·18) | 0·74 | >0·99 |
| First EEG, n/total |  | |  |  |  |  |  |  |  |
| Focal abnormality | 91/262 | | 77/86 | 14/176 | 84·6 | 94·7 | 32·83 (19·28 to 55·91) | *****<0·0001** | ****0·0026** |
| Consistent with encephalitis | 15/262 | | 8/86 | 7/176 | 53·3 | 68·4 | 6·96 (4·23 to 11·44) | 0·094 | >0·99 |
| Low CSF:serum glucose ratio, n/total | 91/261 | | 30/86 | 61/175 | 33·0 | 67·1 | 1·00 (0·58 to 1·72) | >0·99 | >0·99 |
| CSF:serum glucose ratio, mean (SD) | 0·55 (0·11) | | 0·55 (0·11) | 0·55 (0·10) |  |  |  | 0·81 | >0·99 |
| Low initial serum sodium, n/total | - | | - | - | - | - | - | - |  |
| Initial serum sodium, mmol/L, mean (SD) | - | | - | - | - | - | - | - |  |
| High initial serum lactate, n/total | - | | - | - | - | - | - | - |  |
| Initial serum lactate, mmol/L, mean (SD) | - | | - | - | - | - | - | - |  |
| Taking anti-seizure medication prior to admission, n/total | - | | - | - | - | - | - | **-** |  |

*Table S14:* A summary of Türkiye study group separated into patients that did and did not have a seizure with univariate analysis. Hyphens indicate missing data or an inability to complete statistical analysis

Analysis completed using student’s t-test or fisher’s exact test.

Bold font highlight significant differences (* p<0·05; ** p<0·01; *** p<0·001) after Bonferroni correction for multiple analyses, shown as adjusted p-value.

*Abbreviations: acute disseminating encephalomyelitis (ADEM), cerebrospinal fluid (CSF), electroencephalogram (EEG), Glasgow Coma Scale (GCS), Herpes*  *simplex virus (HSV), interquartile range (IQR), magnetic resonance imaging (MRI), negative predictive value (NPV), odds ratio (OR), positive predictive value (PPV), standard deviation (SD), tuberculosis (TB), Varicella zoster virus (VZV).*

| Variable | All (n=55) | Seizure (n=14) | No seizure (n=41) | P value | Adjusted p-value |
| --- | --- | --- | --- | --- | --- |
| Age, years, median (IQR)^+^ | 65 (45–73) | 60·5 (45–72) | 65 (44–72·5) | 0·84 | >0·99 |
| Age, years n (%) |  |  |  | 0·052 | 0·99 |
| ≤5 | 0 | 0 | 0 |  |  |
| >5 to ≤18 | 1 (2) | 1 (7) | 0 (0) |  |  |
| >18 to ≤40 | 9 (16) | 0 (0) | 9 (22) |  |  |
| >40 to ≤60 | 15 (27) | 6 (43) | 9 (22) |  |  |
| >60 | 30 (55) | 7 (50) | 23 (56) |  |  |
| Sex, n (%) |  |  |  |  |  |
| Male | 31 (56) | 67 (55) | 42 (51) | 0·560 | >0·99 |
| **Aetiology, n (%)** |  |  |  | *****0·0006** | ***0·012** |
| Autoantibody-associated | **4 (7)** | **0 (0)** | **4 (10)** |  |  |
| HSV | **8 (15)** | **6 (43)** | **2 (5)** |  |  |
| Other immune | **8 (15)** | **0 (0)** | **8 (20)** |  |  |
| Other infectious | **15 (27)** | **2 (14)** | **13 (32)** |  |  |
| Unknown | **12 (22)** | **5 (36)** | **7 (17)** |  |  |
| VZV | **7 (13)** | **0 (0)** | **7 (17)** |  |  |
| Syphilis | **1 (1)** | **1 (7)** | **0 (0)** |  |  |
| Ethnicity, n (%) |  |  |  |  |  |
| White | 55 (100) | (100) | (100) |  |  |
| Immunosuppressed, n (%) | 8 (15) | 2 (14) | 6 (14) | 0·67 | >0·99 |
| HIV positive, n (%) | 0 | 0 | 0 |  |  |
| **Symptoms** |  |  |  |  |  |
| Median symptom duration, days (IQR, range)^+^ | 2 (0–4, 0–90) | 1 (0–2, 0-5) | 2·5 (1–5·5, 0–90) | *0·020 | 0·30 |
| GCS at presentation, n/total^+^ |  |  |  |  |  |
| ≤8 | 5/55 | 2/14 | 3/41 | 0·19 | >0·99 |
| 9–12 | 2/55 | 1/14 | 1/41 | 0·28 | >0·99 |
| 13–14 | 29/55 | 8/14 | 21/41 | 0·28 | >0·99 |
| 15 (ref) | 18/55 | 2/14 | 16/41 | - | - |
| Symptoms, n (%) |  |  |  |  |  |
| Lethargy | 19 (35) | 5 (36) | 14 (34) | 0·47 | >0·99 |
| Personality/ behavioural change | 29 (53) | 5 (36) | 24 (59) | 0·24 | >0·99 |
| Stiff neck | 1 (2) | 0 (0) | 1 (2) | 0·70 | >0·99 |
| Headache | 16 (29) | 1 (7) | 15 (37) | *0·020 | 0·46 |
| Irritability | 1 (2) | 0 (0) | 1 (2) | 0·61 | >0·99 |
| Fever | 34 (62) | 9 (64) | 25 (61) | 0·83 | >0·99 |
| Any focal deficit on neurological examination^+^ | 15 (27) | 4 (29) | 11 (27) | >0·99 | >0·99 |
| Gastrointestinal | 4 (7) | 1 (7) | 3 (7) | 0·84 | >0·99 |
| Respiratory | 3 (6) | 0 (0) | 3 (7) | 0·48 | >0·99 |
| Photophobia | 5 (9) | 0 (0) | 5 (12) | 0·32 | >0·99 |
| Investigations and treatment |  |  |  |  |  |
| MRI, n/total |  |  |  |  |  |
| Abnormal | 21/38 | 7/14 | 14/14 | 0·15 | >0·99 |
| EEG, n/total |  |  |  |  |  |
| Abnormal | 37/46 | 13/14 | 24/32 | 0·24 | >0·99 |
| Consistent with encephalitis | 19/46 | 5/14 | 14/32 | 0·75 | >0·99 |
| Focal changes | 18/46 | 8/14 | 10/32 | 0·12 | >0·99 |
| CSF, n/total |  |  |  |  |  |
| Low glucose (<2·2mmol/l)  Table S15: A summary of Finland study group separated into patients that did and did not have a seizure with univariate analysis.  Analysis completed using student’s t-test or fisher’s exact test.  Bold font highlight significant differences (* p<0·05; ** p<0·01; *** p<0·001) after Bonferroni correction for multiple analyses, shown as adjusted p-value.  *Abbreviations: cerebral spinal fluid(CSF), electroencephalogram (EEG), Glasgow Coma Score (GCS), Herpes simplex virus (HSV), negative predictive value (NPV), positive predictive value (PPV).* | 0/55 | 0/13 | 0/39 | - | - |

| **Variable** | **All (n=10)** | | **Seizure (n=5)** | **No seizure (n=5)** | **PPV** | **NPV** | **OR (95% CI)** | **p-value** |  |  |  |  |
| --- | --- | --- | --- | --- | --- | --- | --- | --- | --- | --- | --- | --- |
| **Demographic**  Age, years, median (IQR) | 23·5 (19-36·25) | **34 (18-37)** | | **21 (19-26)** |  |  | *Not calculated due to low n* | *Not calculated due to low n* |  |  |  |  |
| Age, years, n (%)  ≤ 5 | 0 (0·0) | | 0 (0·0) | 0 (0·0) | - | 50·0 |  |  |  |  |  |  |
| >5 to ≤ 18 | 2 (20·0) | | 2 (40·0) | 0 (0·0) | 100·0 | 62·5 |  |  |  |  |  |  |
| >18 to ≤ 40 | 6 (60·0) | | 2 (40·0) | 4 (80·0) | 33·3 | 25·0 |  |  |  |  |  |  |
| >40 to ≤ 60 | 2 (20·0) | | 1 (20·0) | 1 (20·0) | 50·0 | 50·0 |  |  |  |  |  |  |
| >60 | 0 (0·0) | | 0 (0·0) | 0 (0·0) | 0·0 | 50·0 |  |  |  |  |  |  |
| Sex, n (%)  Male | 1 (10·0) | | 0 (0·0) | 1 (20·0) | 0·0 | 44·4 |  |  |  |  |  |  |
| **Aetiology, n (%)**  Autoantibody-associated | 7 (70·0) | | 3 (60·0) | 4 (80·0) | 42·9 | 33·3 |  |  |  |  |  |  |
| HSV | 0 (0·0) | | 0 (0·0) | 0 (0·0) | 0·0 | 50·0 |  |  |  |  |  |  |
| VZV | 0 (0·0) | | 0 (0·0) | 0 (0·0) | 0·0 | 50·0 |  |  |  |  |  |  |
| Bacterial | 0 (0·0) | | 0 (0·0) | 0 (0·0) | 0·0 | 50·0 |  |  |  |  |  |  |
| ADEM/immune | 2 (20·0) | | 1 (20·0) | 1 (20·0) | 50·0 | 50·0 |  |  |  |  |  |  |
| Mycobacterium TB | 0 (0·0) | | 0 (0·0) | 0 (0·0) | 0·0 | 50·0 |  |  | |  | | |
| Infection (other) | 1 (10·0) | | 1 (20·0) | 0 (0·0) | 100·0 | 55·6 |  |  | |  |  |  |
| Unknown | 0 (0·0) | | 0 (0·0) | 0 (0·0) | 0·0 | 50·0 |  |  | |  |  |  |
| Immunosuppressed, n (%) | 2 (20·0) | | 1 (20·0) | 1 (20·0) | 50·0 | 50·0 |  |  | |  |  |  |
| HIV positive, n (%) | 0 (0·0) | | 0 (0·0) | 0 (0·0) | 0·0 | 50·0 |  |  | |  |  |  |
|  |  | |  |  |  |  |  |  | |  |  |  |
| **Symptoms**  Median symptom duration prior to admission, days (IQR, range) | **-** | | **-** | **-** |  |  |  | **-** | |  |  |  |
| GCS at presentation, n/total |  | |  |  |  |  |  |  | |  |  |  |
| ≤ 8 | 0/10 | | 0/5 | 0/5 | 0·0 | 50·0 |  |  | |  |  |  |
| 9-12. | 1/10 | | 0/5 | 1/5 | 0·0 | 44·4 |  |  | |  |  |  |
| 13-14 | 2/10 | | 1/5 | 1/5 | 50·0 | 50·0 |  |  | |  |  |  |
| 15 (ref) | 3/10 | | 4/5 | 3/5 | 57·1 | 66·7 |  |  | |  |  |  |
| Temperature over 38°C on admission, n/total | 0/10 | | 0/5 | 0/5 | 0·0 | 50·0 |  |  | |  |  |  |
| **Presenting symptoms, n (%)**  Fever | 1 (10) | | 0 (0·0) | 1 (20·0) | 0·0 | 44·4 |  |  | |  |  |  |
| Headache | 3 (30·0) | | 2 (40·0) | 1 (10·0) | 66·7 | 57·1 |  |  | |  |  |  |
| Lethargy | 0 (0·0) | | 0 (0·0) | 0 (0·0) | 0·0 | 50·0 |  |  | |  |  |  |
| Seizure | 10 (100·0) | | 5 (100·0) | 5 (100·0) | 50·0 | 0·0 |  |  | |  |  |  |
| Behavioural change | 7 (70·0) | | 4 (80·0) | 3 (60·0) | 57·1 | 66·7 |  |  | |  |  |  |
| Focal neurology | 7 (70·0) | | 3 (60·0) | 4 (80·0) | 42·9 | 33·3 |  |  | |  |  |  |
| Irritability | 3 (30·0) | | 1 (20·0) | 2 (40·0) | 33·3 | 42·9 |  |  | |  |  |  |
| Neck stiffness | 0 (0·0) | | 0 (0·0) | 0 (0·0) | 0·0 | 50·0 |  |  | |  |  |  |
| Photophobia | 0 (0·0) | | 0 (0·0) | 0 (0·0) | 0·0 | 50·0 |  |  | |  |  |  |
| Gastrointestinal | 0 (0·0) | | 0 (0·0) | 0 (0·0) | 0·0 | 50·0 |  |  | |  |  |  |
| Respiratory | 1 (10·0) | | 1 (20·0) | 0 (0·0) | 100·0 | 55·6 |  |  | |  |  |  |
| **Diagnostic tests** |  | |  |  |  |  |  |  | |  |  |  |
| First MRI, n/total |  | |  |  |  |  |  |  | |  |  |  |
| Abnormal | 4 (40·0) | | 2 (40·0) | 2 (40·0) | 50·0 | 60·0 |  |  | |  |  |  |
| First EEG, n/total |  | |  |  |  |  |  |  | |  |  |  |
| Focal abnormality | **-** | | **-** | **-** | **-** | **-** |  |  | |  |  |  |
| Consistent with encephalitis | **-** | | **-** | **-** | **-** | **-** |  |  | |  |  |  |
| Low CSF:serum glucose ratio, n/total | **-** | | **-** | **-** | **-** | **-** |  |  | |  |  |  |
| CSF:serum glucose ratio, mean (SD) | **-** | | **-** | **-** | **-** | **-** |  |  | |  |  |  |
| Low initial serum sodium, n/total | 0 (0·0) | | 0 (0·0) | 0 (0·0) | 0·0 | 55·6 |  |  | |  |  |  |
| Initial serum sodium, mmol/L, mean (SD) | 139·9 (3·30) | | 138·8 (2·22) | 140·8 (3·96) |  |  |  |  | |  |  |  |
| High initial serum lactate, n/total | - | | - | - | - | - |  |  | |  |  |  |
| Initial serum lactate, mmol/L, mean (SD) | - | | - | - | - | - |  |  | |  |  |  |
| Taking anti-seizure medication prior to admission, n/total | - | | - | - | - | - |  |  | |  |  |  |

*Table S16:* A summary of the Russia study group separated in patients that did and did not have a seizure.

No statistical analysis was completed due to the low sample size. Hyphens indicate missing data.

*Abbreviations: acute disseminating encephalomyelitis (ADEM), cerebrospinal fluid (CSF), electroencephalogram (EEG), Herpes simplex virus (HSV), interquartile range (IQR), magnetic resonance imaging (MRI), negative predictive value (NPV), odds ratio (OR), positive predictive value (PPV), standard deviation (SD), tuberculosis (TB), Varicella zoster virus (VZV)*

| **Variable** | **Inpatient Seizure**  **N=210** | **Absent inpatient Seizure N=410** | **P-value** | **Adjusted p-value** |
| --- | --- | --- | --- | --- |
| Age median  Age >60 | 46  29% (61/210) | 50  34.4% (141/410) | 0.10  0.18 | >0.99  >0.99 |
| Gender (Female) | 48.1% (101/210) | 50.2% (206/410) | 0.61 | >0.99 |
| Race:  White  African American  Hispanic  Asian  Other | 45.7% (96)  29% (61)  11.9% (25)  1.9% (4)  11.5% (24) | 46.1% (189)  22.7% (93)  16.8% (69)  4.1% (17)  10.3% (42) | *0.036 | 0.76 |
| Etiology:  **Viral**  **Autoimmune**  Unclear  **Other (ex: bacterial, fungal..)** | **23.8% (50/210)**  **26.9% (56/208)**  42.8% (89/208)  6.2% (13/208) | **37.5% (150/400)**  **15.4% (61/396)**  35.8% (141/396)  **11.9% (47/396)** | *****<0.0010**  *****<0.0010**  0.084  *0.028 | *** <0.021**  *** <0.021**  >0.99  0.59 |
| Immunocompromised | 24.3% (51/210) | 21.7% (89/410) | 0.47 | >0.99 |
| Charlson comorbidity index <2 | 55.2% (116/210) | 52.7% (216/410) | 0.55 | >0.99 |
| GCS < or = 8  GCS > or = 14 | 11.2%  49.5% | 16.1%  57.4% | 0.10  0.07 | >0.99  >0.99 |
| Presenting report:  Fever  Focal neuro signs  **Seizure at presentation**  **Headache**  **Confusion**  **Psychiatric symptoms**  Memory deficits  Sleep disturbances | 52.7% (107/203)  43.3% (91/210)  **70.7% (147/208)**  **46.9% (82/175)**  **85.2% (179/210)**  **66.2% (139/210)**  33.7% (67/199)  10.5% (22/209) | 57.1% (224/392)  36.9% (150/407)  **17.9% (73/407)**  **56.6% (210/371)**  **74% (304/409)**  **56.3% (231/410)**  30.4% (122/401)  12% (49/409) | 0.30  0.12  *****<0.0010**  *0.033  **0.0060  *0.018  0.42  0.59 | >0.99  >0.99  ***<0.021**  0.63  0.13  0.38  >0.99  >0.99 |
| Abnormal MRI at admission | 57.9% (111/195) | 56.9% (189/332) | 0.46 | >0.99 |

*Table S17:* A summary of the pre-analysed ‘USA group’, stratified into seizing and non-seizing patients with univariate analysis.

Bold font highlight significant differences (* p<0.05; ** p<0.01; *** p<0.001) after Bonferroni correction for multiple analyses, shown as adjusted p-value.

*Abbreviations: acute disseminating encephalomyelitis (ADEM), cerebrospinal fluid (CSF), electroencephalogram (EEG), Glasgow Coma Scale (GCS), Herpes simplex virus (HSV), interquartile range (IQR), magnetic resonance imaging (MRI), negative predictive value (NPV), odds ratio (OR), positive predictive value (PPV), tuberculosis (TB), United States of America (USA), Varicella zoster virus (VZV).*

| **Variable** | **All (n=455)** | **Seizure (n=152)** | | **No seizure (n=303)** | **PPV** | **NPV** | **OR (95% CI)** | **p-value** | **Adjusted p-value** |  |
| --- | --- | --- | --- | --- | --- | --- | --- | --- | --- | --- |
| Age, years, median (IQR) | 44 (25-63) | 37 (24-60·25) | | 47 (26·5-64) |  |  |  | 0·10 | >0·99 |  |
| Age, years, n (%)  ≤ 5 | 19 (4·2) | 4 (2·6) | | 15 (5) | 21·1 | 49·1 | 0·26 (0·08 to 0·78) | 0·067 | >0·99 |  |
| >5 to ≤ 18 | 50 (10·9) | 17 (11·2) | | 33 (10·9) | 34·0 | 48·6 | 0·49 (0·26 to 0·89) |  |  |  |
| >18 to ≤ 40 | 147 (32·2) | 62 (40·8) | | 84 (27·7) | 42·5 | 47·6 | 0·67 (0·46 to 0·98) |  |  |  |
| >40 to ≤ 60 | 107 (23·4) | 31 (20·4) | | 75 (24·8) | 29·2 | 45·6 | 0·35 (0·22 to 0·55) |  |  |  |
| >60 | 134 (29·3) | 38 (25·0) | | 96 (31·7) | 28·4 | 43·9 | 0·31 (0·20 to 0·47) |  |  |  |
| Sex, n (%)  Male | **173 (37·9)** | **41 (27)** | | **132 (43·6)** | **23·7** | **39·5** | **0·20 (0·14 to 0·30)** | ***** <0·0001** | ****0·0031** |  |
| **Aetiology, n (%)**  Autoantibody-associated | **118 (25·8)** | **83 (54·6)** | | **34 (11·2)** | **70·9** | **55·0** | **2·98 (1·93 to 4·62)** | *****<0·0001** | ****0·0031** |  |
| HSV | **20 (4·4)** | **3 (2)** | | **17 (5·6)** | **15·0** | **48·8** | **0·17 (0·05 to 0·58)** |  |  |  |
| VZV | **9 (2)** | **0 (0)** | | **9 (3)** | **0·0** | **49·2** | **-** |  |  |  |
| Bacterial | **13 (2·8)** | **0 (0)** | | **16 (5·3)** | **0·0** | **48·9** | **-** |  |  |  |
| ADEM/immune | **38 (8·3)** | **17 (11·2)** | | **20 (6·6)** | **45·9** | **49·7** | **0·84 (0·43 to 1·64)** |  |  |  |
| Mycobacterium TB | **3 (0·7)** | **1 (0·7)** | | **2 (0·7)** | **33·3** | **49·9** | **0·50 (0·04 to 5·53)** |  |  |  |
| Infection (other) | **38 (8·3)** | **5 (3·3)** | | **33 (10·9)** | **13·2** | **47·5** | **0·14 (0·04 to 0·36)** |  |  |  |
| Unknown | **215 (47)** | **43 (28·3)** | | **172 (54·6)** | **20·0** | **54·6** | **0·30 (0·20 to 0·46)** |  |  |  |
| Immunosuppressed, n (%) | 8 (1·8) | 7 (4·6) | | 1 (0·3) | 87·5 | 50·5 | 7·14 (0·87 to 58·41) | 0·069 | >0·99 |  |
| HIV positive, n (%) | 2 (0·4) | 2 (1·3) | | 0 (0) | 100·0 | 50·2 | - | 0·50 | >0·99 |  |
|  |  |  | |  |  |  |  |  |  |  |
| **Symptoms**  Median symptom duration prior to admission, days (IQR, range) | 44·5 (10-266·25, 2-10024) | 21 (6·5-277, 2-10024) | 88 (44·5-228·5, 2-4988) | | - | - | - | 0·65 | >0·99 |  |
| GCS at presentation, n/total |  |  | |  |  |  |  |  |  |  |
| ≤ 8 | 66/455 | 21/152 | | 45/303 | 31·8 | 47·8 | 1·02 (0·56 to 1·82) | >0·99 | >0·99 |  |
| 9-12. | 84/455 | 31/152 | | 53/303 | 36·9 | 47·9 | 1·28 (0·75 to 2·20) | 0·41 | >0·99 |  |
| 13-14 | 101/455 | 36/152 | | 65/303 | 35·6 | 47·1 | 1·21 (0·73 to 1·97) | 0·52 | >0·99 |  |
| 15 (ref) | 204/455 | 64/152 | | 140/303 | 31·4 | 40·5 | - | - |  |  |
| Temperature over 38°C on admission, n/total | 57/423 | 16/134 | | 41/289 | 28·1 | 67·8 | 0·82 (0·44 to 1·52) | 0·65 | >0·99 |  |
| **Presenting symptoms, n (%)**  Fever | **190 (41·8)** | **45 (29·6)** | | **145 (47·9)** | **23·7** | **59·6** | **0·46 (0·30 to 0·69)** | *****0·0002** | ****0·0062** |  |
| Headache | 36 (35·6) | 26 (37·1) | | 9 (29·0) | 74·3 | 33·3 | 1·44 (0·58 to 3·61) | 0·50 | >0·99 |  |
| Lethargy | 20 (19·8) | 17 (24·3) | | 3 (9·67) | 85·0 | 34·6 | 2·99 (0·81 to 11·10) | 0·11 | >0·99 |  |
| Seizure | 76 (75·2) | 56 (80·0) | | 18 (58·1) | 75·7 | 48·1 | 2·89 (1·15 to 7·27) | * 0·029 | 0·93 |  |
| Behavioural change | 50 (49·5) | 31 (44·3) | | 19 (61·3) | 62·0 | 23·5 | 0·50 (0·21 to 1·19) | 0·13 | >0·99 |  |
| Focal neurology | 19 (19·0) | 14 (20·0) | | 5 (16·7) | 73·7 | 30·9 | 1·25 (0·41 to 3·85) | 0·78 | >0·99 |  |
| Irritability | 23 (22·8) | 15/70 (21·4) | | 7(22·5) | 68·2 | 30·4 | 0·94 (0·34 to 2·59) | >0·99 | >0·99 |  |
| Neck stiffness | 13 (12·9) | 12 (17·1) | | 1 (3·22) | 92·3 | 34·1 | 6·21 (0·77 to 50·04) | 0·061 | >0·99 |  |
| Photophobia | 10 (10·1) | 10 (14·5) | | 0 (0·0) | 100·0 | 33·7 | - | * 0·030 | 0·93 |  |
| Gastrointestinal | 25 (25·0) | 15 (21·7) | | 10 (32·3) | 60·0 | 28·0 | 0·58 (0·23 to 1·50) | 0·32 | >0·99 |  |
| Respiratory | 5 (4·95) | 4 (5·71) | | 1 (3·23) | 80·0 | 31·3 | 1·82 (0·19 to 16·97) | >0·99 | >0·99 |  |
| **Diagnostic tests** |  |  | |  |  |  |  |  |  |  |
| First MRI, n/total |  |  | |  |  |  |  |  |  |  |
| Abnormal | 72/100 | 50/69 | | 21/31 | 70·4 | 34·5 | 1·25 (0·50 to 3·14) | 0·64 | >0·99 |  |
| First EEG, n/total |  |  | |  |  |  |  |  |  |  |
| Focal abnormality | 61/97 | 47/70 | | 14/27 | 77·0 | 36·1 | 2·93 (0·93 to 8·81) | 0·10 | >0·99 |  |
| Consistent with encephalitis | 21/97 | 15/70 | | 6/27 | 71·4 | 27·6 | 2·19 (0·55 to 8·18) | 0·31 | >0·99 |  |
| Low CSF:serum glucose ratio, n/total | 24/73 | 18/47 | | 6/26 | 75·0 | 40·8 | 2·07 (0·70 to 6·13) | 0·21 | >0·99 |  |
| CSF:serum glucose ratio, mean (SD) | 0·58 (0·19) | 0·56 (0·20) | | 0·64 (0·14) | **-** | **-** | **-** | 0·10 | >0·99 |  |
| Low initial serum sodium, n/total | 0/99 | 0/69 | | 0/30 | - | - | - | - | - |  |
| Initial serum sodium, mmol/L, mean (SD) | 137·9 (4·74) | 138·4 (3·90) | | 136·7 (6·29) | - | - | - | 0·10 | >0·99 |  |
| High initial serum lactate, n/total | 12/60 | 12/44 | | 0/16 | - | - | - | *0·025 | 0·93 |  |
| Initial serum lactate, mmol/L, mean (SD) | 2·69 (2·76) | 3·12 (3·12) | | 1·60 (0·77) | **-** | **-** | **-** | 0·060 | >0·99 |  |
| Taking anti-seizure medication prior to admission , n/total | 22/101 | 17/70 | | 5/31 | 77·3 | 32·9 | 1·67 (0·55 to 5·02) | 0·44 | >0·99 |  |
| *Table S18:* A summary of the region of the Americas study group separated in patients that did and did not have a seizure with univariate analysis.  Analysis was completed using student’s t-test or fisher’s exact test.  Bold font highlight significant differences (* p<0·05; ** p<0·01; *** p<0·001) after Bonferroni correction for multiple analyses, shown as adjusted p-value.  *Abbreviations: acute disseminating encephalomyelitis (ADEM), cerebrospinal fluid (CSF), electroencephalogram (EEG), Glasgow Coma Scale (GCS), Herpes simplex virus (HSV), interquartile range (IQR), magnetic resonance imaging (MRI), negative predictive value (NPV), odds ratio (OR), positive predictive value (PPV), standard deviation (SD), tuberculosis (TB), Varicella zoster virus (VZV).* |  |  | |  |  |  |  |  |  |  |
