# Supplementary material for: International evaluation of the SEIZUre Risk in Encephalitis (SEIZURE) score for predicting acute seizure risk
Source: BMJ Open. 2025 Dec 7;15(12):e099451. doi: 10.1136/bmjopen-2025-099451 (PMC12699598; doi:10.1136/bmjopen-2025-099451)
Supplement: Supplementary file 2 [file bmjopen-2025-099451.reviewer_comments.pdf]

PEER REVIEW HISTORY

BMJ Open publishes all reviews undertaken for accepted manuscripts. Reviewers are asked to complete a checklist review form and are provided with free text boxes to elaborate on their assessment. These free text comments are reproduced below.

This paper was submitted to another journal from BMJ but declined for publication following peer review. The authors addressed the reviewers' comments and submitted the revised paper to BMJ Open. The paper was subsequently accepted for publication at BMJ Open.

ARTICLE DETAILS

Title (Provisional)

An international evaluation of the SEIZUre Risk in Encephalitis (SEIZURE) score for predicting acute seizure risk

Authors

hughes, thomas; Venkatesan, Arun; Hetherington, Claire; Egbe, Franklyn Nkongho; Netravathi, M; Thakur, Kiran T.; Baykan, Betul; Hui Jan, Tan; Arias, Susana; García-de Soto, Jesús; Kahwagi, Jamil; Vogrig, Alberto; Versace, Salvatore; Habis, Ralph; Sowmitran, Swathi; Husari , Khalil S; Probasco, John; Hasbun, Rodrigo; Bean, Paris; Heck, Ashley; GözübatıkÇelik, Gökçen R.; ataklı, dilek; Mayda Domac, Fusun; Ferreira, Vitor; Calado, Sofia; Sangeeth, Thuppanattumadam Ananthasubramanian; Defres, Sylviane; Romozzi, Marina; Iorio, Raffaele; Pensato, Umberto; Pleshkevich, Maria; Steriade, Claude; Sharifi-Razavi, A.; Tabrizi, Nasim; Sipila, Jussi; Kim, Carla Y.; Diaz-Ariza, Alexandra; Satish, Poorvikha; Gowda, Vinutha; Gowda, Chandrakanta; Oh, Seong-il; del Capio-Orantes, Luis; Cotelli, Mariasofia; Ferreira, Luís; Kovalchuk, Maria; Goncharova, Anna; Solomon, Tom; Winkler, Andrea; Guekht, Alla; Wood, Greta K.; NeuroResearch Coalition, The Global; Michael, Benedict Daniel

VERSION 1 - REVIEW

**Reviewer**	**1**
**Name**	**Reviewer 1**
**Affiliation**	
**Date**	**07-Dec-2024**
**COI**	**None**

Comments not published pending reviewer's request.

**Reviewer**	**2**

**Name**	**Fong, Michael**
**Affiliation**	**University of Western Sydney**
**Date**	**07-Dec-2024**
**COI**	**None**

Thank you for the opportunity to review Hughes et.al. who described the assessment of a previously reported SEIZURE score in its ability to reproducibly determine acute seizures in 2,032 patients with encephalitis from 13 countries.

The modelling and presentation of the data is sound, but I had comments that relate to the concept behind the model/ outcomes and the interpretation of the results.

The SEIZURE score was reported in 2022, but greater emphasis should be placed that the basis of the components of the score have been well described over several decades and are not at all specific to encephalitis. For example, Claassen et.al. described age, coma, prior seizure as risk factors for any seizure in the ICU two decades ago, and the Misra et.al. study quoted from 2009 demonstrates similar features. The only specific feature in this score compared to prior is "etiology" of encephalitis that was only present in the advanced score.

Regarding outcome, it was difficult to determine from the data presented the regional heterogeneity. One major limitation is that the seizure probability varied greatly between regions, e.g., SEA 12%, 38% in The America's and Europe, and 46% in Africa. It was not clear whether this was differences in etiology or just differences in definitions/ clinical practice between the regions. For example, was the seizure rate 12% in SEA because none of these patients got EEG and were discharged from hospital after 2 days, or was the rate 46% in Africa because it was clinically determined many had seizures with a low rate of EEG confirmation. Given the composite outcome of "inpatient seizures", greater detail should be disclosed such as rates of clinical seizure only, rates of EEG seizure, time of admission and durations of EEG recording for each region, length of stay etc.

I appreciate the main aim of this study was to assess the reproducibility of the SEIZURE score in different world regions. One critical limitation has to be that in this cohort the EEG seemed superior to the SEIZURE score in its determination of seizures. The EEG findings were not included in subsequent modelling but comparing the univariate analysis: Advanced SEIZURE score, age - none significant, fever – not significant, GCS ≤8 OR 1.87, etiology - only significant for antibody associated OR3.77. When taking the score overall even the highest score 12+ global had a univariate OR of 1.93. Compare this to EEG Focal abnormality OR 32.83 and EEG consistent with encephalitis OR 6.96. As it is written there is little acknowledgement that the EEG was probably the best predictor of seizures in this cohort, just not assessed in modelling.

The conclusions and proposed implications should be tempered. Even at its best the AUROC for The Americas and Europe was only 0.67, just above the pre-determined 0.65 cut off for "acceptable". Implying this score has great clinical utility in predicting seizures or even as suggested used to determine who should be administered prophylactic antiseizure medications seems premature. The conclusion that it is not valid in other regions also seems premature. Looking at the univariate, the predictive strength only comes from having a score of 12+. Africa only had 2 patients had a score of 12+ and neither had seizure, and Eastern mediterranian 1 patient with a score of 12+. Clinical features like prior seizure, coma, and age have been replicated in many settings as determinants of inpatient seizures, it is likely the current study is underpowered in particular regions to be able to adequately demonstrate these associations.

The number in the abstract is misleading, 2,032 patients were identified, but the SEIZURE score was only calculated in 1,324 patients with regression modelling only using 970 patients.

VERSION 1 - AUTHOR RESPONSE

Reviewer: 2

Thank you for the opportunity to review Hughes et.al. who described the assessment of a previously reported SEIZURE score in its ability to reproducibly determine acute seizures in 2,032 patients with encephalitis from 13 countries.

The modelling and presentation of the data is sound, but I had comments that relate to the concept behind the model/ outcomes and the interpretation of the results.

We thank the Reviewer for their time taken and their positive comments on the paper.

1) The SEIZURE score was reported in 2022, but greater emphasis should be placed that the basis of the components of the score have been well described over several decades and are not at all specific to encephalitis. For example, Claassen et.al. described age, coma, prior seizure as risk factors for any seizure in the ICU two decades ago, and the Misra et.al. study quoted from 2009 demonstrates similar features. The only specific feature in this score compared to prior is "etiology" of encephalitis that was only present in the advanced score.

Response: Thank you for the suggestion on further expansion of the prior literature in the introduction. In the revised manuscript we have expanded on the Misra et al.

study and other, non-encephalitis, studies that assess general risks of seizures. These changes can be found on page 4, lines 16-19, and are quoted directly below:

‘Prior research has established age and GCS are risk factors for seizures of any cause, and also specifically in encephalitis patients.[2,5-7] The SEIZURE score was the outcome of the first study to combine this prior knowledge into an applicable, validated, risk stratifying tool for UK encephalitis patients.’

2) Regarding outcome, it was difficult to determine from the data presented the regional heterogeneity. One major limitation is that the seizure probability varied greatly between regions, e.g., SEA 12%, 38% in The America’s and Europe, and 46% in Africa. It was not clear whether this was differences in etiology or just differences in definitions/ clinical practice between the regions. For example, was the seizure rate 12% in SEA because none of these patients got EEG and were discharged from hospital after 2 days, or was the rate 46% in Africa because it was clinically determined many had seizures with a low rate of EEG confirmation. Given the composite outcome of “inpatient seizures”, greater detail should be disclosed such as rates of clinical seizure only, rates of EEG seizure, time of admission and durations of EEG recording for each region, length of stay etc.

Response: We agree with the reviewer’s insight that the variable seizure rates made comparisons between regions difficult. In the revised manuscript we have expanded the discussion around this limitation and implications for future research.

Firstly we have explicitly stated the limitation on page 18, lines 1-4 as quoted below:

‘The ability to analyse regional heterogeneity was limited by wide inter-regional variations in seizure rates, even within the same encephalitis aetiology. This could explain the poor performance of the SEIZURE score globally. Variance may stem from symptom duration, time until management, availability of neurology expertise, and EEG access, particularly for subtle motor or non-convulsive seizures.’

Secondly, we acknowledged the need for future studies to standardise the clinical and electrographic seizure classification and the extent to which EEG was used which is included on page 18, lines 6-8, as quoted below:

‘To better analyse regional heterogeneity, future studies should specify whether inpatient seizures were diagnosed clinically or via EEG, detailing durations of EEG recording in regions with readily-available monitoring.’

3) I appreciate the main aim of this study was to assess the reproducibility of the SEIZURE score in different world regions. One critical limitation has to be that in this cohort the EEG seemed superior to the SEIZURE score in its determination of seizures. The EEG findings were not included in subsequent modelling but comparing the univariate analysis: Advanced SEIZURE score, age - none significant, fever – not significant, GCS ≤8 OR 1.87, etiology - only significant for antibody associated OR3.77. When taking the score overall even the highest score 12+ global had a univariate OR of 1.93. Compare this to EEG Focal abnormality OR 32.83 and EEG consistent with encephalitis OR 6.96. As it is written there is little acknowledgement that the EEG was probably the best predictor of seizures in this cohort, just not assessed in modelling.

Response: Thank you for highlighting the utility of EEG in seizure prediction in our study. The omission in modelling, and subsequent discussion, was due to the lack of easy access to EEG in several of the regions which could bias statistical modelling analysis, notably Senegal's complete absence of EEG. However, we appreciate that the strength of its predictive power on the global cohort should not be overlooked and have revised the manuscript to include detail on this at several points.

Firstly, we noted that variables including EEG were not included in modelling if they were not readily available in all regions (page 6, line 3).

Secondly, we noted the likely utility of EEG in seizure prediction, whilst also citing prior literature, and the potential implications of not having readily available access in the context of the global encephalitis treatment gap. This can be found on page 18, lines 35-38, as quoted below.

'Our results highlight the global treatment gap for encephalitis and seizures, with particularly poor outcomes in AFR and SEAR, where the disease burden is likely highest. These regions had fewer EEG data which limits the diagnosis of subtle seizures. However, in our global cohort EEG abnormalities carried a higher seizure risk than a high SEIZURE score, highlighting the need to improve global EEG access.'

Thirdly, we acknowledged the potential value of EEG as an added variable in future variations of the SEIZURE scoring system in countries that have EEG availability. This is noted on page 19, lines 17-20, as quoted below.

‘Additional variables could be included, particularly presenting with a seizure and EEG abnormalities for regions with reliable access, both of which carried a higher risk of having an inpatient seizure than patients in the highest category of the SEIZURE score in the global cohort’

4) The conclusions and proposed implications should be tempered. Even at its best the AUROC for The Americas and Europe was only 0.67, just above the pre-determined 0.65 cut off for “acceptable”. Implying this score has great clinical utility in predicting seizures or even as suggested used to determine who should be administered prophylactic antiseizure medications seems premature.

Response: The Reviewer’s comments on the conclusion are appreciated. In the revised manuscript, the wording has been adjusted in several areas to ensure that when referencing the good discrimination of the score this is only in relation to Western Europe and not Europe as a whole (page 17, lines 30-31; page 19, lines 13-14 and line 25).

The scores performance in the Americas is now only described as good in the context of the differentiating the highest risk patients as this cohort had significant increased odds ratios with adequate sample sizes (page 17, line 31).

Furthermore, we clarified that in many regions, including the Americas, the score only just passed as the predefined level of 'acceptable' and suggest exercising caution in spite of Western Europe’s results. This is found on page 19, lines 8-10, as quoted below.

‘Despite promising overall results within Western Europe, the poor performance in Spain warrants caution prior to widespread use and in many regions, including the Americas, the discrimination ability only just reached ‘acceptable’.’

Finally, the conclusion surrounding using the scoring system to determine who should receive primary ASM prophylaxis was reworded to ensure further studies are completed prior to this. This is noted on page 19, lines 20-23, and also on page 19, lines 25-28, as quoted below.

‘Ultimately, regions that can demonstrate a localised scoring system with good discriminatory ability in prospective trials should aim to conduct randomised trials of ASM prophylaxis in encephalitis patients deemed at high-risk of seizures.’

‘The SEIZURE score performed variably internationally, with good potential in most of Western Europe, albeit prospective trials are needed. Other regions should develop local scoring systems with larger cohorts to improve the discriminatory ability. This will facilitate future trials on the utility of ASM prophylaxis and targeting investigations to high-risk patients.’

5) The conclusion that it is not valid in other regions also seems premature. Looking at the univariate, the predictive strength only comes from having a score of 12+. Africa only had 2 patients had a score of 12+ and neither had seizure, and Eastern mediterranian 1 patient with a score of 12+. Clinical features like prior seizure, coma, and age have been replicated in many settings as determinants of inpatient seizures, it is likely the current study is underpowered in particular regions to be able to adequately demonstrate these associations.

Response: Thank you for the suggestions surrounding the conclusion. In the revised manuscript we have now included that the study may be underpowered in particular settings and needed a larger sample size whilst also acknowledging the role that different aetiologies will play.

The general instances are on page 17 line 31-33, page 19 lines 10-15, page 19 lines 26-27, and in the abstract as quoted below.

‘The poorer performance elsewhere was likely partly due to underpowered cohorts, particularly the proportion with a SEIZURE score >12, and differing epidemiology, confirming that many regions require localised scoring systems.’

‘Whilst the overall cohort number was large, the inclusion of a single centre or country is unlikely to be representative of the entire region. Therefore, some of the regional analysis was likely underpowered but the discriminatory ability may be improved with increased sample sizes. Future research should focus on prospective trials and refining the scoring system within Western Europe. Bespoke scoring systems should be created for other regions with larger regional sample sizes and standardised case ascertainment methodology.’

‘Other regions should develop local scoring systems with larger cohorts to improve the discriminatory ability.’

(Abstract) ‘Beyond Western Europe there is need for tailored, localised scoring systems, and future large-scale prospective studies with optimised aetiological testing to accurately identify high-risk patients.’

The effect of underpowering was already stated for Spain specifically regarding GCS on page 17, lines 41-42, but in the revised manuscript we have added this regarding age when discussing why fever may also not have been identified as significant. This is noted on page 18, lines 30-33, as quoted below.

'Fever is an established seizure risk factor in children. The relatively low proportion of children, compared to *Wood et al.,* may have underpowered our study.[7] This would explain why age was often not a significant risk factor despite prior literature.'

6) The number in the abstract is misleading, 2,032 patients were identified, but the SEIZURE score was only calculated in 1,324 patients with regression modelling only using 970 patients.

Response: Thank you for highlighting this oversight. This has been corrected as quoted below (page 3, lines11-12).

(Abstract) '2032 patients across 13 countries were identified, of whom 1324 were included in SEIZURE score calculations and 970 were included in regression modelling.'

VERSION 2 - REVIEW

**Reviewer**	**2**
**Name**	**Fong, Michael**
**Affiliation**	**University of Western Sydney**
**Date**	**22-Mar-2025**
**COI**	**None**

Thank you for the opportunity to re-review Hughes et.al. who described "An international evaluation of the SEIZUre Risk in Encephalitis (SEIZURE) score for predicting acute seizure risk" including considerable revisions based on comments from two independent reviewers.

All of my comments have been adequately addressed and valuable changes to the manuscript have been made. I do not have any further suggestions.

**Reviewer**	**3**
**Name**	**Ho, Lai-Ming**

Affiliation **The University of Hong Kong, School of PUblic Health**

Date **09-Jul-2025**

COI **None**

Thank you for giving me the opportunity to review this paper. This is one of the largest cohorts in encephalitis research examining the utility of Seizure score for predicting acute seizure risk. This cohort is comprised of patients from centres in different regions, resulting in data heterogeneity, which affects generalizabily of the research findings. My concerns and comments are based on the epidemiological/statistical perspectives.

1. A total of 2,032 patients were identified (Line 5 on page 9), but only 1,379 were included in the analysis due to insufficient raw data to calculate the Seizure Score. In other words, approximately 30% of patients were excluded from the present study. It would be useful to compare the characteristics of those included in the analysis with those excluded to assess potential biases in the sample and to understand how these biases might affect the utility of the Seizure Score for predicting acute seizure risk. Additionally, employing multiple imputation to fill in missing data and re-analyzing the dataset as a sensitivity analysis may provide valuable insights.

2. The PPV values were quite low, as shown in Table 3 (page 16), with most being less than 50%. This indicates a significant likelihood that a positive result could be a false positive, suggesting that the test may have limited reliability in confirming the presence of the health condition.

3. In the Statistical Analysis section (Line 44 on page 8), it would be helpful not only to mention that the cluster analysis is hierarchical but also to include additional details relevant to the cluster analysis. For example, specify the algorithm used (eg, k-means) and provide its justification, describe the similarity measure employed (eg, Euclidean distance) and its rationale, and explain how the optimal number of clusters was determined.

4. The reference categories in Table 2 appear confusing and inconsistent. For example, in lines 3-17 on page 13, there is no indication of the reference category for the odds ratio (OR), but the “1.00” in “Age > 60” suggests it is the reference for the OR in the “multivariate binary logistic regression”. Additionally, in line 48 on page 14, “(ref)” is used to indicate a reference category. Please note that it should be “multivariable binary logistic regression” instead of “multivariate binary logistic regression”.

5. In Table 3 (Lines 6-7 on pagr 16), the score category has two “(REF)”. What does it mean?

6. In Europe (Lines 22-25 on page 19), the PPV was approximately 40% in both the basic and advanced scoring systems (Table 3). The AUROC ranged from around 0.6 in Spain to 0.8 in Portugal. Additionally, it is noted that the basic score performed poorly in Europe with an AUROC of 0.62 (95% CI 0.57–0.67), and the improved, acceptable advanced score had an AUROC of 0.67 (95% CI 0.62–0.71) (Lines 18-20 on page 19). The evidence supporting the

statement that "…promising overall results within Western Europe" appears to be relatively weak, as the AUROC values are not high enough to be considered clinically useful (Çorbacioglu SK, Aksel G. Receiver operating characteristic curve analysis in diagnostic accuracy studies: A guide to interpreting the area under the curve value. Turk J Emerg Med. 2023 Oct 3;23(4):195-198). The conclusion that the score "showed good discrimination" should be tempered, especially given regional performance variability and the "acceptable" discrimination threshold.

7. Line 10 on page 22, which states that "some of the regional analysis was likely underpowered but the discriminatory ability may be improved with increased sample sizes" is not fully justified. An increase in sample size primarily affects statistical significance, and may not necessarily lead to an improvement in discriminatory ability.

VERSION 2 - AUTHOR RESPONSE

Reviewer: 2

Comments to the Author:
Thank you for the opportunity to re-review Hughes et.al. who described "An international evaluation of the SEIZUre Risk in Encephalitis (SEIZURE) score for predicting acute seizure risk" including considerable revisions based on comments from two independent reviewers. All of my comments have been adequately addressed and valuable changes to the manuscript have been made. I do not have any further suggestions.

Many thanks for taking the time to re-review this study and for your comments regarding the valuable changes we have made to the manuscript.

Reviewer: 3

Comments to the Author:
Thank you for giving me the opportunity to review this paper. This is one of the largest cohorts in encephalitis research examining the utility of Seizure score for predicting acute seizure risk. This cohort is comprised of patients from centres in different regions, resulting in data heterogeneity, which affects generalisability of the research findings. My concerns and comments are based on the epidemiological/statistical perspectives.

Thank you for taking the time to review the paper which consists of one of the largest sample sizes for any encephalitis study. We acknowledge the heterogeneity which forms one of the main conclusions of our study.

1. A total of 2,032 patients were identified (Line 5 on page 9), but only 1,379 were included in the analysis due to insufficient raw data to calculate the Seizure Score. In other words, approximately 30% of patients were excluded from the present study. It would be useful to compare the characteristics of those included in the analysis with those excluded to assess potential biases in the sample and to understand how these biases might affect the utility of the Seizure Score for predicting acute seizure risk. Additionally, employing multiple imputation to fill in missing data and re-analyzing the dataset as a sensitivity analysis may provide valuable insights.

Thank you for this comment. There were ultimately very few patients excluded from the seizure score as detailed in figure 2. Whilst 1379 patients had raw data provided to calculate the seizure score, of the remaining 654 patients, only 15 were missing the data required to be included in the study. The additional 639 were provided by a group from the USA. The data was pre-analysed in accordance with an agreed statistical approach, due to ethical approval barriers, and so we did not receive the raw data. The data was provided tabulated with statistical tests already applied which can be seen in supplementary data (Table S15).

The sentence has been amended on page6 lines 16-17 to add:

"(…), 639 patients were pre-analysed due to ethical approval reasons, and 15 were excluded due to missing required data."

Given that the 15 excluded patients makes up only 0.73% of the total cohort, multiple imputation was not completed. However, we have acknowledged that missing data could be a limitation in the study and suggested for future studies to employ this analysis when large proportions of patients are missing clinically important variables as quoted below (page 20 lines 3-5)

"Additionally, this reduced the number of participants excluded from the study due to key missing data. Whilst this negated the need for multiple imputation analysis this may be beneficial in future large scale studies with higher proportions of participants with clinically important missing data."

2. The PPV values were quite low, as shown in Table 3 (page 16), with most being less than 50%. This indicates a significant likelihood that a positive result could be a false positive, suggesting that the test may have limited reliability in confirming the presence of the health condition.

Thank you for this valuable insight. To be more transparent we have stated in the text which areas had a PPV of over 0.50 on page12 lines 7-13 as quoted below:

"A PPV of over 0.50 was observed in the basic score for Africa (scores 9-11), Eastern Mediterranean (9-11) and the highest scoring category in Western Pacific, USA group, and Europe. In the advanced scoring system, a PPV >0.50 was also calculated in Eastern Mediterranean (scores 14-16) and the highest scoring categories of Western Pacific, Region of the Americas, USA group, and Europe. After Bonferroni correction in these groups, statistical significance was observed only in Europe, USA group and Region of the Americas."

Additionally we have included the low ppv in the discussion whilst also highlighting that in the highest scoring patients of the USA and Europe, the PPV was often >0.50. We have gone on to further suggest this may highlight the use of the test as a screening tool as opposed to a confirmatory test (Page 20, lines16-21). This is then further acknowledged in the final conclusion (page 20 lines 40-41). Both quotes are below:

"Furthermore, when creating scoring categories, both globally and across many regions, the scores demonstrated a low PPV of <0.50, suggesting limited reliability in predicting the risk of an inpatient seizure. Although the PPV was often >0.50 among high scoring patients in Europe and the USA, the general trend of lower PPVs globally suggests the score may be better utilised as a screening tool to identify patients who may benefit from closer monitoring rather than as a confirmatory test of seizure risk."

"This will facilitate future trials on the utility of ASM prophylaxis and screening for high-risk patients to target further investigations."

3. In the Statistical Analysis section (Line 44 on page 8), it would be helpful not only to mention that the cluster analysis is hierarchical but also to include additional details relevant to the cluster analysis. For example, specify the algorithm used (eg, k-means) and provide its justification, describe the similarity measure employed (eg, Euclidean distance) and its rationale, and explain how the optimal number of clusters was determined.

Many thanks for the useful suggestion. The original Rstudio script was reviewed to confirm the exact methodology and has been included, alongside the rationale, on page 6 lines 6-12. It is quoted below:

“R-studio was used for creating heatmaps of group-associated variables amongst patients who had inpatient seizures and hierarchical agglomerative cluster analysis. This was performed using the average linkage method and Euclidean distance as the similarity measure. The optimal number of clusters was determined by visual inspection of the dendrogram and assessment with the silhouette plot. The rationale was to compare to the analyses done in the original paper and facilitate an exploratory analysis with flexibility, ease of interpretability and the ability to reveal natural groupings.”

4. The reference categories in Table 2 appear confusing and inconsistent. For example, in lines 3-17 on page 13, there is no indication of the reference category for the odds ratio (OR), but the “1.00” in “Age > 60” suggests it is the reference for the OR in the “multivariate binary logistic regression”. Additionally, in line 48 on page 14, “(ref)” is used to indicate a reference category. Please note that it should be “multivariable binary logistic regression” instead of “multivariate binary logistic regression”.

Many thanks for highlighting the confusion this may cause which was likely due to the results of separate analyses being combined on a single table. The table was originally two separate tables but was combined for conciseness. We have elaborated on how the odds ratios were calculated for univariate analysis and multivariate analysis in the methods section as quoted below on page 5 line 34-37 and page 6 line 5-6).

“When calculating OR for variables with multiple discrete categories, including age and aetiology, the reference value was defined as the combined sum of the remaining categories. The exceptions for this were GCS, which used 15 as a reference score (...).”

[in relation to multivariable binary logistic regression] *“The reference categories for the OR for age and aetiology were identical to those used in the Wood et al. study”*

We have also altered all wording of multivariate to multivariable throughout the paper.

5. In Table 3 (Lines 6-7 on pagr 16), the score category has two “(REF)”. What does it mean?

REF refers to the the reference values used for statistical analysis when conducting chi squared/fishers exact test and calculating odds ratios. This is in keeping with the values used in the original Wood *et al.* study. We have elaborated in our methods section to highlight this to avoid confusion as quoted below (page 5, line 34-37) and expanded on it as an abbreviation for “reference baseline score” in table 3.

“When calculating OR for variables with multiple discrete categories, including age and aetiology, the reference value was defined as the combined sum of the remaining categories. The exceptions for this were GCS, which used 15 as a reference score, and the SEIZURE score categories, which used a baseline reference score in keeping with the original Wood *et al*. study.”

6. In Europe (Lines 22-25 on page 19), the PPV was approximately 40% in both the basic and advanced scoring systems (Table 3). The AUROC ranged from around 0.6 in Spain to 0.8 in Portugal. Additionally, it is noted that the basic score performed poorly in Europe with an AUROC of 0.62 (95% CI 0.57–0.67), and the improved, acceptable advanced score had an AUROC of 0.67 (95% CI 0.62–0.71) (Lines 18-20 on page 19). The evidence supporting the statement that “…promising overall results within Western Europe” appears to be relatively weak, as the AUROC values are not high enough to be considered clinically useful (Çorbacioglu SK, Aksel G. Receiver operating characteristic curve analysis in diagnostic accuracy studies: A guide to interpreting the area under the curve value. Turk J Emerg Med. 2023 Oct 3;23(4):195-198). The conclusion that the score "showed good discrimination" should be tempered, especially given regional performance variability and the "acceptable" discrimination threshold.

Thank you for highlighting the need to adjust the conclusions made. We have altered our wording to more neutral conclusions, particularly changing from “promising” and “good” to “acceptable”. We have acknowledged even the acceptable reasons may have limited clinically utility and when discussing the good performance of Western Europe, we have done so only in comparison to other regions analysed. Importantly, we have also adjusted our final conclusion in line with the above changes. These can be found on page 3 line 17-19, page 18, line 30-32, page 20, line 14-16, page 20, line 36-39. They are quoted below, respectively:

“The SEIZURE score performed best in several countries in Western Europe but performed poorly elsewhere, partly due to differing and unknown aetiologies. In most regions, the score did not reach a threshold to be clinically useful.”

“The score showed acceptable discrimination in western Europe, excluding Spain, and identified high-risk patients in AMR. However, even in these areas, the results suggest that it has a limited clinical utility in its current format.”

“Despite many of the countries in Western Europe performing better than other regions, the poor performance in Spain warrants caution prior to widespread use and in many areas, including the Americas, the discrimination ability only just reached ‘acceptable’.”

“The SEIZURE score performed variably across international settings, with the best performance observed in several Western European countries. However, in most regions and countries, the scoring system fell below the threshold typically considered clinically useful. Even in areas where the performance was acceptable, prospective validation trials remain necessary.”

7. Line 10 on page 22, which states that “some of the regional analysis was likely underpowered but the discriminatory ability may be improved with increased sample sizes” is not fully justified. An increase in sample size primarily affects statistical significance, and may not necessarily lead to an improvement in discriminatory ability.

Thank you for highlighting this oversight. The wording has been adjusted to state a larger sample size would allow us to *more reliably* conclude there is a lack of discriminatory ability. This is quoted on line 20, page 22-24 as:

“Therefore, some of the regional analysis was likely underpowered and could be evaluated with larger sample sizes prior to reliably concluding the lack of discriminatory ability.”

VERSION 3 - REVIEW

**Reviewer**	**3**
**Name**	**Ho, Lai-Ming**
**Affiliation**	**The University of Hong Kong, School of PUblic Health**
**Date**	**20-Sep-2025**
**COI**	

Thank you for revising the manuscript. It is much clearer now. I have no further comments except for the following three points:

1) In Table 2, it is clear that age > 60 is used as the reference category, indicated by an OR of "1". Therefore, the additional explanation stating that "... age and aetiology, the reference value was defined as the combined sum of the remaining categories" may be unnecessary, as it is a usual practice to use "1" to denote the reference category.

2) In Table 2, GCS is divided into 4 categories: <=8, 9–12, 13–14, and 15. If "GCS=15" is used as the reference, it is unusual that "1" is not used to indicate that this is the reference category. Additionally, there are no "OR (95% CI)" in Column 7 or "p-value (adjusted)" in Column 8 for "GCS=15," nor are there "odds ratios (95% CI)" in Column 9 for the other GCS categories. It seems that GCS may have been treated as a continuous variable in the multivariable logistic regression, with 15 as the reference value. If that is the case, it should be clearly explained in the statistical analysis section, and a clearer table presentation would enhance readers' understanding.

3) In Table S5, the presence of two 'REF' is unclear. For example, in line 6 on page 39 of 109, the OR for Global="7-8" is 1.06. Which category is used as the reference: "0-2" or "3-6"?

VERSION 3 - AUTHOR RESPONSE

Reviewer: 3

Comments to the Author:
Thank you for revising the manuscript. It is much clearer now. I have no further comments except for the following three points:

Thank you for taking the time to re-review the paper in such detail.

1) In Table 2, it is clear that age > 60 is used as the reference category, indicated by an OR of "1". Therefore, the additional explanation stating that "... age and aetiology, the reference value was defined as the combined sum of the remaining categories" may be unnecessary, as it is a usual practice to use "1" to denote the reference category.

Thank you for this comment. The age >60 was used as the reference category for the multivariable analysis only, consistent with the Wood *et al.* study. This has been expanded on in the statistical methodology section on page 6 line 13 as quoted below:

"The reference categories for the OR for age and aetiology were identical to those used in the Wood *et al*. study and are marked as OR= 1·00 for clarification."

The univariate analysis used a reference range as the combined sum of the remaining categories. This has been highlighted in the statistical methodology section on page 5, line 40 as quoted below:

"For univariate analysis, when calculating OR for variables with multiple discrete categories, including age and aetiology, the reference value was defined as the combined sum of the remaining categories."

2) In Table 2, GCS is divided into 4 categories: <=8, 9–12, 13–14, and 15. If "GCS=15" is used as the reference, it is unusual that "1" is not used to indicate that this is the reference category. Additionally, there are no "OR (95% CI)" in Column 7 or "p-value

(adjusted)" in Column 8 for "GCS=15," nor are there "odds ratios (95% CI)" in Column 9 for the other GCS categories. It seems that GCS may have been treated as a continuous variable in the multivariable logistic regression, with 15 as the reference value. If that is the case, it should be clearly explained in the statistical analysis section, and a clearer table presentation would enhance readers' understanding.

Thank you for noting this. You are correct that GCS 15 was used as the reference range for the univariate analysis. Whilst this was noted in the methodology on page 5 line 42, the OR of 1 and p-value of 1.00 has been added to further clarify when viewing the table separately to the text.

You are correct in noting that GCS was treated as a continuous variable in the multivariable logistic regression. The clarification is in the table legend and also made on page 5, lines 21-23:

"GCS was inputted as an ordinal variable for scoring and categorical variable (normal, 15; mild impairment, 13-14; moderate impairment, 9-12; severe impairment, 3-8) for univariate analysis. For multivariable analysis it was treated as a continuous variable."

The missing OR in column 9 is due to the variable being treated as a continuous variable. The OR written in column 9 is per point, again consistent with the original Wood *et al.* study. I have added in the "per point" text and moved the OR (95%CI) value so it is in the "GCS at presentation" row rather than "15 (ref)" row.

3) In Table S5, the presence of two 'REF' is unclear. For example, in line 6 on page 39 of 109, the OR for Global="7-8" is 1.06. Which category is used as the reference: "0-2" or "3-6"?

Thank you for highlight the potential area of confusion. The sum of the two lowest-scoring categories were used as a reference range. For the basic scoring system this was (0-2) and (3-6) and for the advanced scoring system it was (0-4) and (5-11). They were not combined into one category on the table to allow visualisation of the distribution across the categories. This is consistent with the approach by Wood *et al.* in the original UK study. I have added in an explanation detailing this in the table legend and in the statistical methodology on page 5, line 43:

"The SEIZURE score categories were defined using the sum of the two lowest-scoring categories as a reference range, consistent with the original Wood *et al.* study. The categories were kept separate to allow for visualisation of the distribution of scores amongst the groups."
